# Manual of praying mantis morphology, nomenclature, and practices (Insecta, Mantodea)

**DOI:** 10.3897/zookeys.696.12542

**Published:** 2017-09-13

**Authors:** Sydney K. Brannoch, Frank Wieland, Julio Rivera, Klaus-Dieter Klass, Olivier Béthoux, Gavin J. Svenson

**Affiliations:** 1 Department of Invertebrate Zoology, Cleveland Museum of Natural History, 1 Wade Oval Drive, Cleveland, Ohio, USA; 2 Department of Biology, Case Western Reserve University, 10900 Euclid Avenue, Cleveland, Ohio, USA; 3 Pfalzmuseum für Naturkunde - POLLICHIA-Museum, Hermann-Schäfer-Str. 17, 67098 Bad Dürkheim, Germany; 4 Universidad San Ignacio de Loyola, Perú; 5 Senckenberg Natural History Collections Dresden, Königsbrücker Landstrasse 159, D-01109 Dresden, Germany; 6 Centre de Recherche sur la Paleobiodiversite et les Paleoenvironnements (CR2P, UMR 7207), Sorbonne Universites, MNHN, CNRS, UPMC-Paris6, Museum National d’Histoire Naturelle, 57 Rue Cuvier, CP 38, 75005 Paris, France

**Keywords:** Mantodea, measurement, morphology, praying mantis, terminology

## Abstract

This study provides a comprehensive review of historical morphological nomenclature used for praying mantis (Mantodea) morphology, which includes citations, original use, and assignment of homology. All referenced structures across historical works correspond to a proposed standard term for use in all subsequent works pertaining to praying mantis morphology and systematics. The new standards are presented with a verbal description in a glossary as well as indicated on illustrations and images. In the vast majority of cases, originally used terms were adopted as the new standard. In addition, historical morphological topographical homology conjectures are considered with discussion on modern interpretations. A new standardized formulation to present foreleg femoral and tibial spines is proposed for clarity based on previous works. In addition, descriptions for methods of collection, curation, genital complex dissection, and labeling are provided to aid in the proper preservation and storage of specimens for longevity and ease of study. Due to the lack of consistent linear morphometric measurement practices in the literature, we have proposed a series of measurements for taxonomic and morphological research. These measurements are presented with figures to provide visual aids with homologous landmarks to ensure compatibility and comparability across the Order. Finally, our proposed method of pinning mantises is presented with a photographical example as well as a video tutorial available at http://mantodearesearch.com.

## 1. Introduction

The central motivation for this work is to produce an updated standard for morphological nomenclature, specimen preparation, and measurement data capture. As there is currently a lack of standardization, some level of confusion exists about term use and application to features as well as the optimal method to measure features that retain highly variable or ambiguous boundaries. We believe this to be an important time to propose a set of standards due to a growth in taxonomic interest in Mantodea, the application of new technologies, and to improve lab workflow efficiency. In addition, we outline and propose new methodological standards to improve the ability to research specimens, which includes specimen preparation and pinning, genital dissections, and labeling.

**Justification to standardize**:

**Coding of morphology**: Congruence of terminology ensures accurate interpretations and future use of characters and their states in morphological analyses and deposition into morphology databases such as MorphBank. The current lack of a system ensures barriers derived from language and chosen reference material. Morphological terminology is suggested using topographical correspondances, which does not in all cases correspond to a hypothesis of homology.

**Formulaic descriptions**: Telegraph style descriptions speed taxonomic work, but term standards ensure longevity, direct comparisons with other studies, reuse of descriptions, and extracting coded characters from descriptions.

**Imaging**: Access to high resolution images of specimens are of great importance to taxonomic work by improving how we gather data, compare specimens, and identify species. However, the way a specimen is dry pinned will have great influence on how many images are needed of the same specimen in order to adequately provide access to the relevant features. Minimizing the number of images captured by standardizing the way a specimen is mounted will greatly increase digitization efficiency and access to feature information. If imaging equipment is not readily available, scientific illustration, when performed with high precision and heeding symmetry in bilaterally symmetric structures, can capture important characters for taxonomic and morphological study.

**Morphometric analysis**: Capture of measurement data requires standardization for broader future use in other analyses based on phylogenetics or species delimitation. The measurements described for suggested use are for linear morphometrics, to be used for taxonomic or morphological purposes, like distinguishing between closely related species. Advanced geometric morphometric measurement techniques might need to be employed for the purposes of functional morphology, ethology, evaluating evolutionary trends in morphological evolution, among others, but those are not proposed here due to their specificity to the hypothesis being tested.

**Data capture**: As label data is often obscured on large specimens, standards of specimen preparation will alleviate this by creating a predictable and optimized downstream approach to capture data on mounted specimens.

## 2. Methods

**Imaging**: All high resolution images of specimens and features were captured using a Passport Storm system (Visionary Digital, 2012), which includes a Stackshot z-stepper, a Canon 5D SLR, macro lenses (50 mm, 100 mm, and MP-E 65 mm), three Speedlight 580EX II flash units, and an associated computer running Canon utility and Adobe Lightroom 3.6 software. The z-stepper was controlled through Zerene Stacker 1.04 and images were processed using the P-Max protocol. Images were captured over an 18% gray card background for white balance standards. Images were processed in Adobe Photoshop CS6 Extended to add scale bars. Adjustments were made using the stamp tool to correct aberrations. Figures depict distinct morphological features, with structures of interest clearly in focus. Measurement delineations were superimposed on the figures using the line tool.

**Illustration**: Illustrations of key morphological structures were digitized using either Adobe Illustrator CS6 and Adobe Photoshop CS6 or CorelDraw and CorelPhotopaint. Diagrammatic illustrations were produced by collecting reference images of the specimens using both the Leica M165C stereo-microscope paired with the IC80 HD camera as well as the Passport Storm, Visionary Digital system. Images were imported into Adobe Illustrator and traced using the pen, paintbrush, and smooth tools. Adobe Illustrator was used for all plate layouts. Illustrations were produced by Rebecca Konte, Josh Maxwell, Hiromi Yagui, and authors SKB, FW, KDK, and OB.

**Measurements**: Standards of measurements were developed using a Leica M165C stereo-microscope and an IC80 HD coaxial video camera using the live measurements module of the Leica Application Suite (LAS).

## 3. Results

**Proposed standardization of morphological nomenclature**

**Figure 1.**
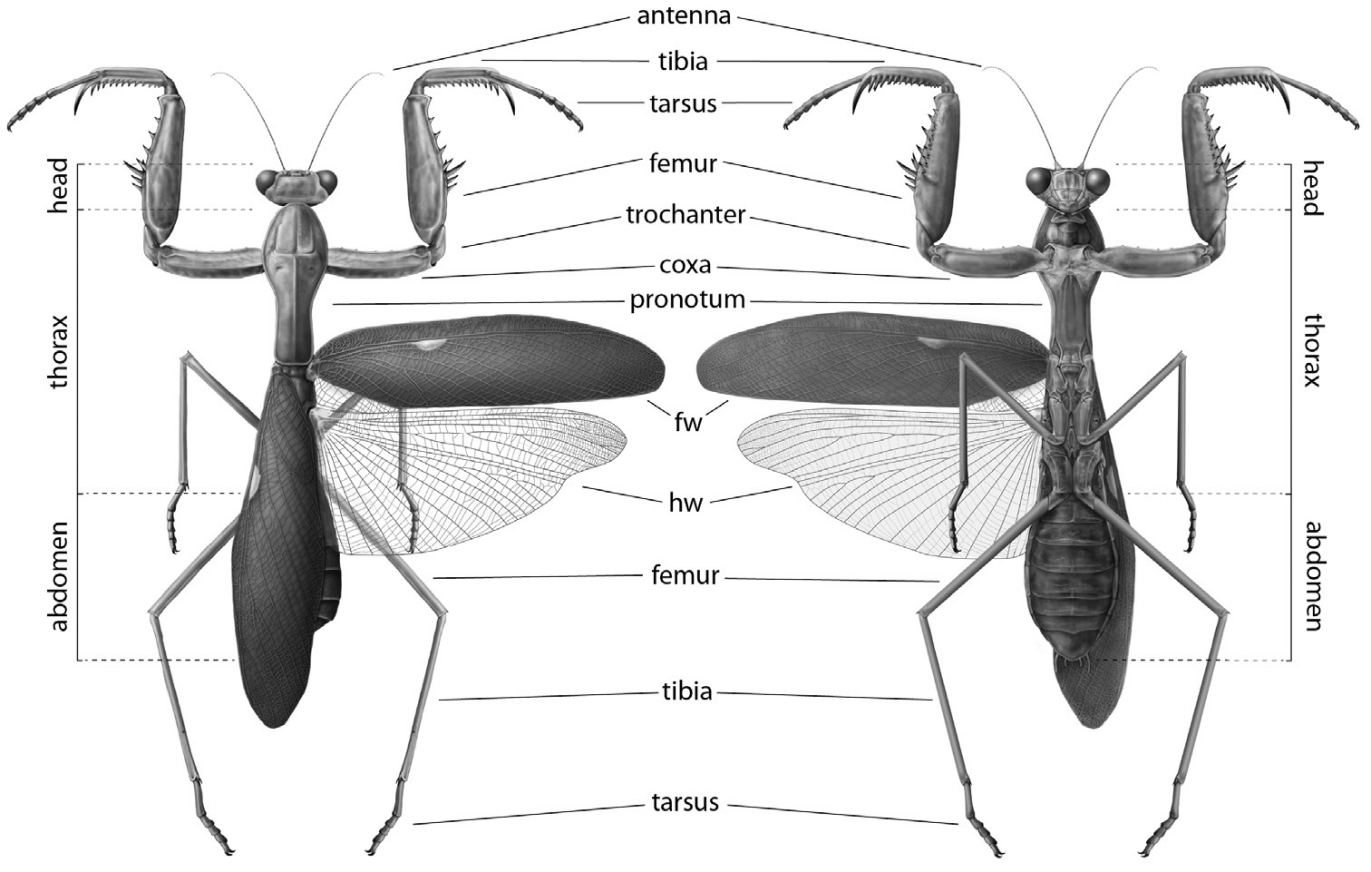
Carbon dust illustrations of *Sphodromantis* sp. dorsal and ventral habitus, from left to right respectively. Illustrations by Rebecca Konte. Abbreviations: **fw** = forewing; **hw** = hindwing.

In the following sections, historical morphological nomenclature of the praying mantis (Fig. 1) is organized by topographically homologous features referenced, described, and illustrated by previous researchers. It is within this framework that we present an updated standard of external and genitalic morphological nomenclature, with preference given to nomenclature described and utilized in influential works; special consideration has been afforded to morphological studies. The terms included in Suppl. material 1–8 are taken directly from the literature; nomenclatural plurality has not been standardized. Sections and tables included in the supplementary materials are organized by major body segments. The terms introduced are defined in the glossary as well as represented in illustrative form.

### 3.1. Head

**Figure 2.**
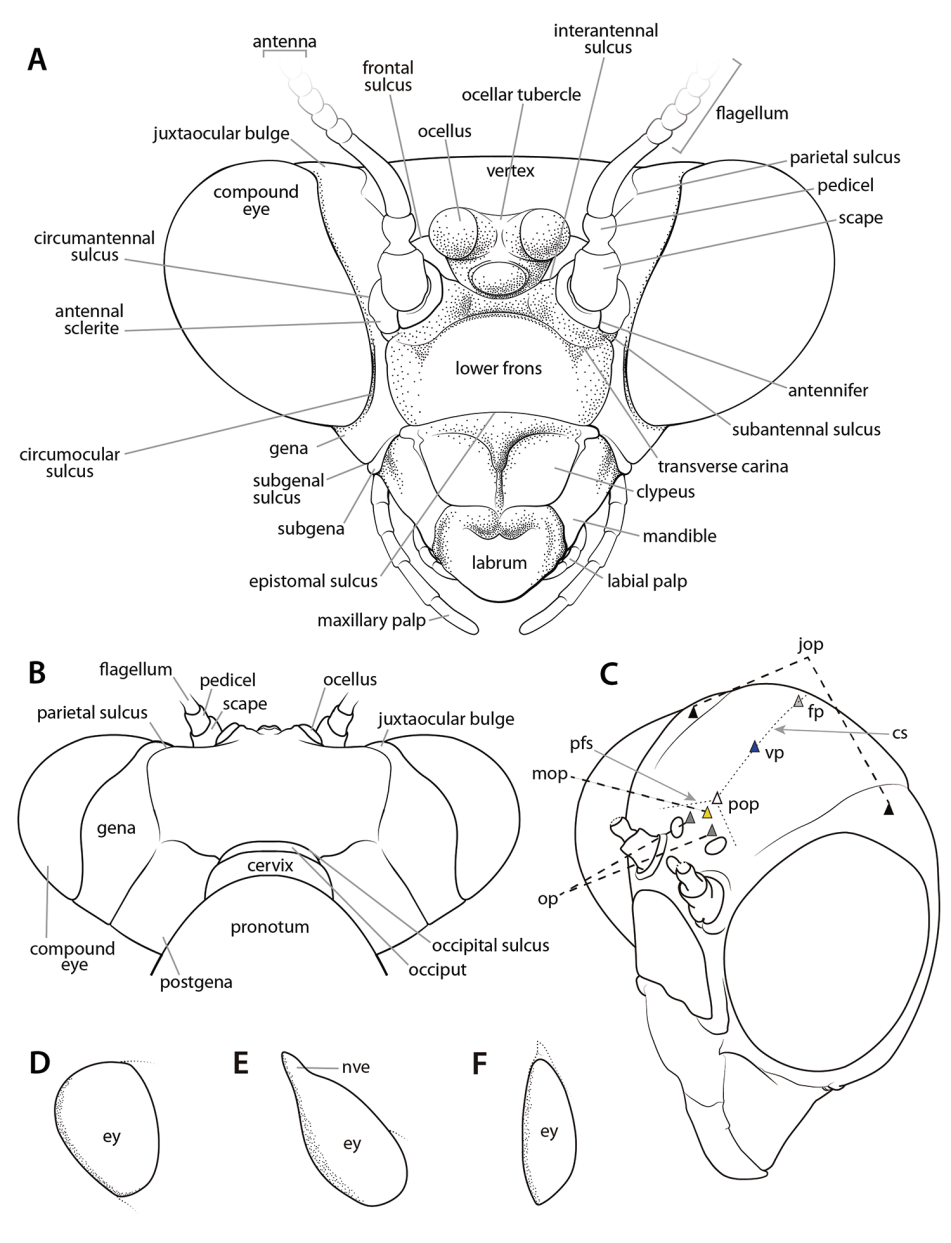
Annotated illustrations of cranial structures. *Omomantis* Saussure, 1899 ♂ head capsule **A** anterior view **B** dorsal view. Generalized mantis head capsule **C** oblique anterior view demarcating regions where cranial process can arise; colored triangles indicate approximate locations of cuticular projections: light gray triangle = fastigial process; black triangles = juxtaocular processes; blue triangle = vertical process; white triangle = postocellar process; yellow triangle = medial ocellar process; gray triangle = ocellar process. Various compound eye shapes (**D**–**F**): **D** approximately globular in *Orthodera* Burmeister, 1838 **E** *Heterochaeta* sp. with a non-visual elongation **F** anteriorly elongate in *Schizocephala bicornis* Linné, 1758. Illustrations **A**–**B** by Josh Maxwell. Abbreviations: **cs** = coronal sulcus; **ey** = compound eye; **fp** = fastigial process; **jop** = juxtaocular process; **mop** = medial ocellar process; **nve** = non-visual elongation; **op** = ocellar process; **pfs** = postfrontal sulcus; **pop** = postocellar process; **vp** = vertical process.

**Figure 3.**
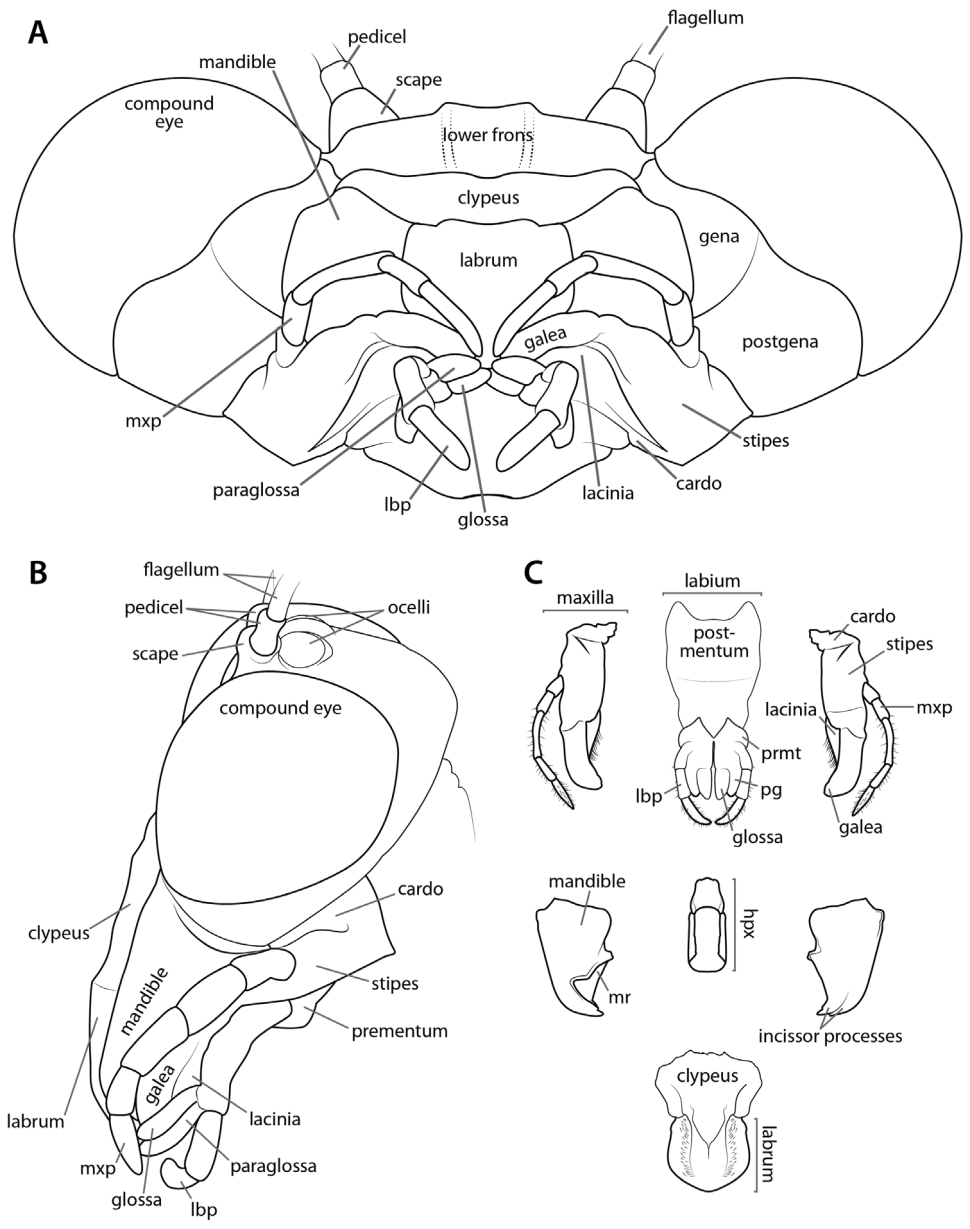
Annotated illustrations of cranial structures. *Omomantis* sp. ♂ cranium **A** anteroventral view; **B** lateral view. *Tenodera sinensis* Saussure, 1871 **C** disarticulated components of mouthparts. Illustrations by Josh Maxwell. Abbreviations: **hpx** = hypopharynx; **lbp** = labial palp; **mr** = molar ridge; **mxp** = maxillary palp; **pg** = paraglossa; **prmt** = prementum.

General cephalic morphological nomenclature is fairly straightforward and without much discord. The structures of the praying mantis head include a pair of antennae, a pair of compound eyes, three ocelli, a lower frons, a clypeus, a labrum, a pair of mandibles, a pair of maxillae and maxillary palpi, as well as a labium and a pair of labial palpi (Figs 2–3). Other features include sulci and sclerotized regions (see Suppl. material 1: Head capsule terminology). Some taxa feature a non-visual elongation on each compound eye (e.g., *Hymenopus coronatus* Olivier, 1792; *Pseudoharpax* Saussure, 1870; *Heterochaeta* Westwood, 1845; *Acanthops* Serville, 1831; etc.). These elongations of the compound eyes are presumably non-visual due to the absence of ommatidia and it has been suggested that they enhance the cryptic appearance of possessor taxa (Edmunds 1972, Wieland 2013) (see Fig. 2e). Many mantodean species feature distinct cranial processes, crests, and tubercles (e.g., Wieland 2013: p. 41). Distinguishing between types of cuticular growths and determining the location from which they arise on the cranium obscures naming these distinct processes. Further complicating matters is that the cuticular growths on the craniums of certain species exhibit various states themselves (e.g., bifurcations, ridges, denticulations, foliaceous outgrowths, etc.) which lend the terminology a nebulous air. Research investigating and homologizing cuticular cranial growths across Mantodea has not been thoroughly conducted. As current interpretations of head processes and growths are ambiguous, we present an annotated figure demarcating regions on the cranium where cuticular processes can arise, including the fastigial, vertical, postocellar, medial ocellar, ocellar, and juxtaocular processes (Fig. 2c). If the entire cranium is extended into a process (e.g., *Phyllocrania* Burmeister, 1838; *Ceratocrania* Westwood, 1889; *Hypsicorypha* Krauss, 1892, etc.), then we suggest referring to that outgrowth as a vertical process. However, if there are multiple cuticular outgrowths originating from various points on the cranium, then it is suggested to refer to the structures as in Fig. 2c. Until a formal ontogenetic study on the development of cranial processes across the Order is undertaken, we suggest the implementation of the cranial process terminology contained herein, but caution that this terminology reflects topographic correspondance and not necessarily true homology.

### 3.2. Wings and wing venation

The wing venation terminology used within this paper follows the serial wing venation pattern (Lameere 1922, 1923), which partly stems from earlier works such as Redtenbacher (1886) and Comstock (1918). It assumes that each main vein comes with two stems (referred to as ‘sectors’), an anterior, convex one, and a posterior, concave one (a main vein and its two sectors form a ‘system’). This pattern was derived from that observed in Palaeozoic extinct palaeopterans in which both branching patterns and vein elevations are organized serially. Lameere (1922, 1923) recognized six paired sectors: costal (**C**) and subcostal (**Sc**); radial (**R**) and sub-radial (**Sr**); median (**M**) and sub-median (**Sm**); cubital (**Cu**) and sub-cubital (**Scu**); penultimate (**P**) and sub-penultimate (**Sp**); and ultimate (**U**) and sub-ultimate (**Su**). Derived from it, the more recent formalization by Kukalová-Peck (1991; see previous accounts by this author) states that anterior and posterior branches are indicated by an ‘**A**,’ or ‘**P**,’ respectively, to follow the abbreviation corresponding to the vein system (e.g., ‘**RA**’ and ‘**RP**’ are the anterior and posterior stems of the radius, respectively). This author recognized eight systems, namely precosta (**PC**), costa (**C**), subcosta (**Sc**), radius (**R**), media (**M**), cubitus (**Cu**), analis (**A**), and juga (**J**). It should be mentioned here that Kukalová-Peck converted Lameere’s main stems of the **C** system (viz., **C** and **Sc**) into distinct systems, and added **PC** to the set. However, in light of the multiple issues with earlier contributions of this author (Rasnitsyn and Novokshonov 1997; Béthoux and Briggs 2008; among others), we believe that the occurrence of a distinct **PC** and **C** is to be re-evaluated.

**Figure 4.**
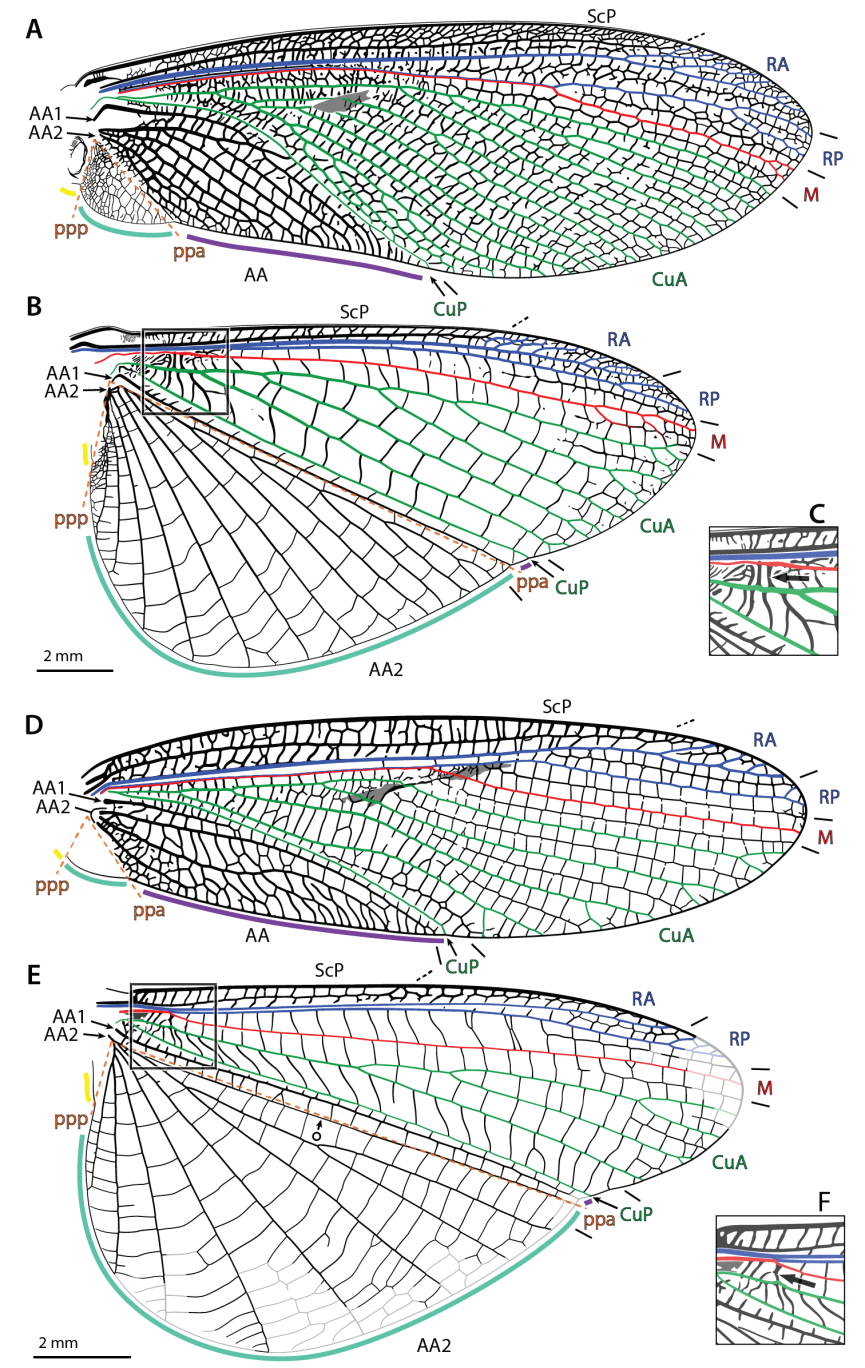
Annotated illustrations of fore- and hindwing venation and structures in *Metallyticus splendidus* Westwood, 1835 (**A**–**C**) and *Chaeteessa* sp. (**D**–**F**). **A, D** forewing **B, E** hindwing (in **E**, º indicates **Iaa_1_-aa_2_**) **C, F** detail of the arculus as located on **B, E** respectively (enlargement 1.5×; black arrow indicates the arculus). Abbreviations: **AA** = anterior Analis; **AA1** = first anterior Analis; **AA2** = second anterior Analis; **CuA** = anterior Cubitus; **CuP** = posterior Cubitus; **M** = Media; **ppa** = plica prima anterior; **ppp** = plica prima posterior; **RA** = anterior radius; **RP** = posterior radius; **ScP** = posterior subcostal; color coded areas: gray = stigma (**sti**); purple = preplicatum; turquoise = plicatum; yellow = plicatulum.

**Figure 5.**
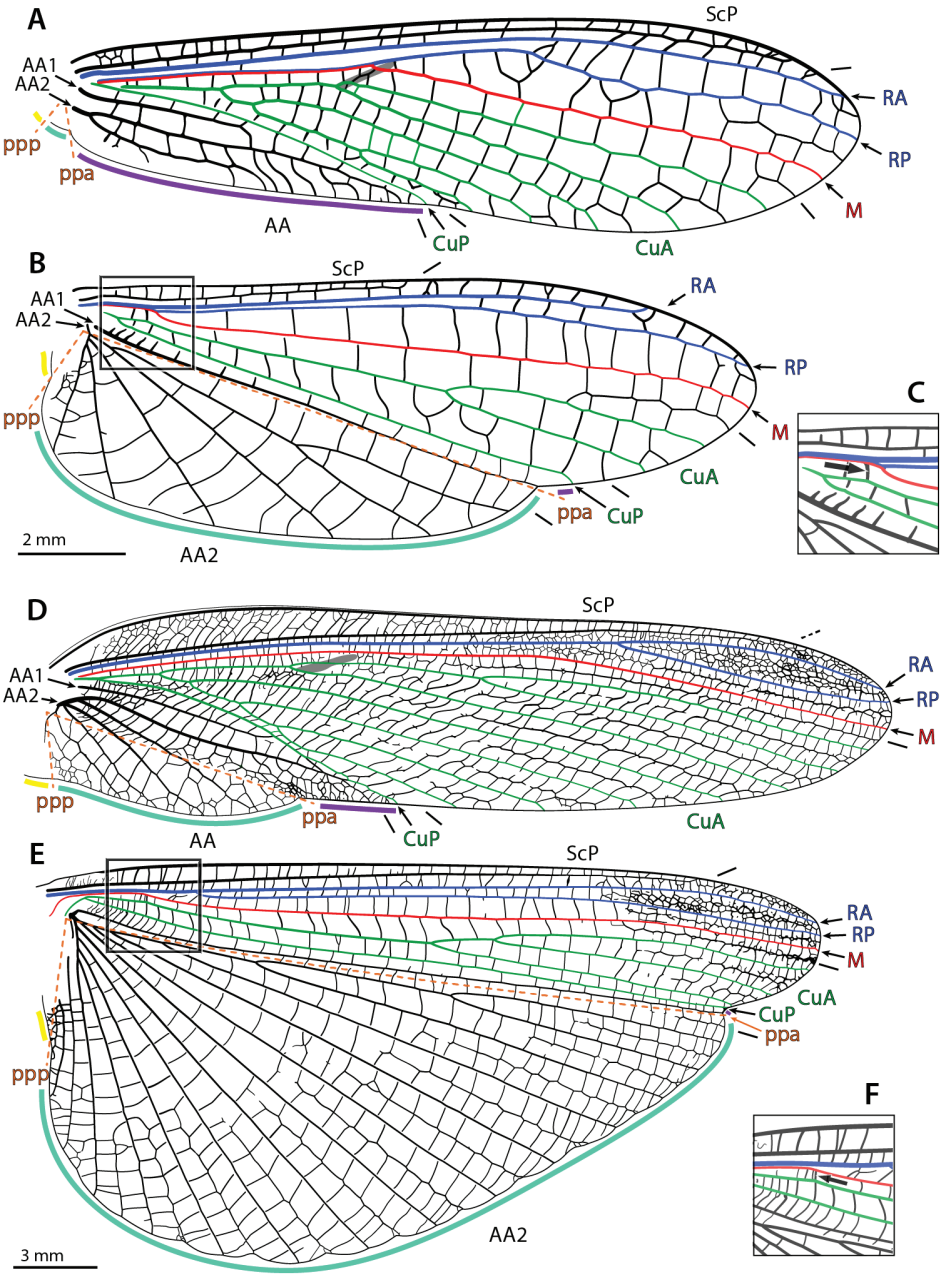
Annotated illustrations of fore- and hindwing venation and structures in *Mantoida maya* Saussure & Zehntner, 1894 (**A**–**C**) and Mantis religiosa (**D**–**F**). **A, D** forewing **B, E** hindwing **C, F** detail of the arculus as located on **B, E** respectively (enlargement 1.5×; black arrow indicates the arculus). Abbreviations: **AA** = anterior Analis; **AA1** = first anterior Analis; **AA2** = second anterior Analis; **CuA** = anterior Cubitus; **CuP** = posterior Cubitus; **M** = Media; **ppa** = plica prima anterior; **ppp** = plica prima posterior; **RA** = anterior radius; **RP** = posterior radius; **ScP** = posterior subcostal; color coded areas: gray = stigma (**sti**); purple = preplicatum; turquoise = plicatum; yellow = plicatulum.

As in other neopteran insects, there is no clear elevation shift between branches of the media in mantises. As a consequence we will refer to an undifferentiated media, **M**. Furthermore, we believe that the occurrence of genuine **AP** branches in fore- and hindwings of neopteran insects is wanting [with the possible exception of *Protophasma dumasii* Brongniart, 1879, revised in Béthoux (2003)]. In summary, we recognize **ScP**, posterior Subcosta; **RA**, anterior Radius; **RP**, posterior Radius; **M**, Media; **CuA**, anterior Cubitus; **CuP**, posterior Cubitus; **AA1**, first anterior Analis (first branch of **AA**, always simple); and **AA2**, second anterior Analis (second branch of **AA**) (see Suppl. material 2–3: Forewing terminology; Hindwing terminology; Figs 4–5).

Sometimes, there can be two rows of cells (delimited by cross-veins) in an area delimited by two veins. It is often the case that the cross-veins delimiting the two rows get aligned and form a vein-like structure. Such structures are referred to as ‘intercalary veins.’ In hindwings they can be aligned with concave folds. They can be referred to using the abbreviations of the surrounding veins written in lowercase (see Béthoux 2015). For example, the particular intercalary vein occurring between **AA1** and the anterior-most branch of **AA2** in *Chaeteessa* Burmeister, 1838 (located along the plica prima anterior –see below) is referred to as ‘**Iaa_1_-aa_2_**’ (Fig. 4e).

**Particular wing structures**

In insect wings it is generally the case that the anterior wing margin is actually a vein-like structure. However, in Mantodea, it is possible that this structure is preceded by a narrow, membranous area. Therefore we distinguish the anterior veinal margin (**avm**) from the anterior wing margin (**awm**). We propose to refer to the area between **awm** and **avm** as the ‘visor’ (**vs**). In the hindwing, the strengthened cross-vein connecting **M** and **CuA** is conventionally referred to as the ‘arculus’ (**arc**).

**Wing venation topographic homology conjectures**

As usual with polyneopteran insects, a broad array of conjectures of topographical homology (THCs) have been proposed to homologize the wing venation of Mantodea with the serial pattern (or any other pattern). In order to facilitate comparison between publications by proponents of various THCs, we describe and discuss the most relevant ones. Our favored THCs for the forewing follow Béthoux and Wieland (2009; themselves followed by Wieland (2013)). Hindwing THCs are more generally consistent with previous accounts except for the posterior area (see below).

There are two major forewing venation patterns among mantises. To ease the following discussion we propose a provisional labelling. The first type, arguably plesiomorphic, encompasses species belonging to *Metallyticus* Westwood, 1835, *Chaeteessa*, *Mantoida* Newman, 1838, and some fossil species (blue shading in Suppl. material 2: Forewing terminology), and will be referred to below as the ‘*MCM*-type.’ In these species the vein (1) (‘**RA**’ in Figs 4a, d, 5a) has no branches or anterior branches only. It is followed by vein (2) (‘**RP**’ + ‘**M**’ in Figs 4a, d, 5a), which is 2-branched (*Chaeteessa* and *Mantoida*; Fig. 4d and 5a, respectively) or with a main fork (*Metallyticus*; Fig. 4a). In the second type, herein referred to as ‘*Mantis*-type’ (orange in shading in Suppl. material 2: Forewing terminology), the vein (1) (‘**RA**’ on Fig. 5d) has a posterior branch (‘vein*’). The veins (1) and (2) are commonly considered as **R** (or part of it) and **M** (or part of it), but to which system ‘vein*’ belongs is debated.

We propose to first address THCs that considered only the *MCM*-type. They will prove weakly supported and some moreover self-inconsistent. Sharov (1962a) stands out with the assumption that **CuA** (as interpreted herein) in both the fore- and hindwing is actually composed of **MP** fused with **CuA**. This proposition is derived from the interpretation that **arc** is **MP**. This author recognized a similar organization in Plecoptera (Sharov 1961, 1962b, 1991) and Orthoptera (Sharov 1968, 1971), in which the forewing, in his opinion, retained evidence of this fusion. However, the corresponding structures were proved to be non-homologous, owing to the presence of trachea, or lack thereof, on the one hand, and the elevation of the connected veins, on the other (Béthoux and Nel 2002; Béthoux 2005, 2008). In Mantodea, **arc** has no trachea and therefore is a cross-vein.

The contribution by Nel and Roy (1996), focusing on a fossil species similar to *Chaeteessa* spp., viz. *Arvernineura insignis* Piton, 1940, and including data on the wing venation of a specimen of *Chaeteessa valida* Perty, 1833, is not straightforwardly accessible. First, on fig. 1, near the wing base, there is label ‘**R** + **MA**’ ambiguously located near two stems that are labeled ‘**RA**’ and ‘**MP**’ in the distal part of the preserved wing. The corresponding description allows the reader to understand that the label ‘**RA**’ refers to the very first branch of the remaining, unlabeled, ‘**RP + MA**.’

Second, one of the figured forewings of a single specimen of *Chaeteessa valida* shows two branches anterior to ‘**RP** + **MA**’ (Nel and Roy 1996: fig. 3a), which is a very unusual configuration, while the other has a single branch (Nel and Roy 1996: fig. 3b) corresponding to ‘**Sc**’ (herein **ScP**) of previous authors. The two unusual branches are interpreted by Nel and Roy (1996) as a short ‘**Sc**,’ and a developed **RA**. However, given that the first branch ends near the origin of the second one and that the second branch ends opposite the end of the usual **ScP**, we assume that **ScP** is branched in the unusual wing and that its posterior branch runs fused with ‘**R** + **MA**’ (herein **RA**). As a consequence, the assumed ‘**RP** + **MA**’ should be understood as ‘**R** + **MA**.’

Third, Nel and Roy (1996) found the number of branches of ‘**CuA**’ to be highly variable in the investigated individual of *Chaeteessa valida* (p. 227). However we counted a consistent number of four and five branches in the represented forewings. Finally it occurred to us that the vein labeled **CuA** (Nel and Roy 1996: figs 1, 2) is referred to as ‘**MP** + **CuA**’ (Nel and Roy 1996: p. 227). All of these issues make reference to this paper uncertain. Therefore we choose to report ambiguous cases regarding *Chaeteessa* wing venation THCs as ‘?’ (see Suppl. material 2: Forewing terminology).

The assumed occurrence of an **R** + **MA** stem by Nel and Roy (1996) is in agreement with the hypothesis of Kukalová-Peck (1991), for whom it is a shared trait of the Dictyoptera, the Paraneoptera, and the Holometabola (see also Kukalová-Peck and Lawrence 2004). However no demonstrative support for such a fusion in the Dictyoptera has ever been published. Note that the scheme reported for Kukalová-Peck (1991) (see Suppl. material 2: Forewing terminology) is based on her interpretation of a forewing of a stem-Dictyoptera. Ambiguities remain on her interpretation of the remigulum in Mantodea (hence the ‘?’ in the corresponding cells; see Suppl. material 2: Forewing terminology).

Although Grimaldi (2003) advocated the legacy of Smart (1956), he engaged in particular THCs under which the vein herein considered as ‘**AA1**’ (convex), alternatively interpreted as **PCu** or **AA1** by previous authors, is considered to be **CuP**, a vein very generally concave in winged insects (notice that this author also referred to this vein as ‘**PCu**’: p. 40). He therefore recognized the vein herein considered as ‘**CuP**’ as a branch of **CuA** (alternatively referred to as ‘**Cu_2_**’ or ‘**CuA_2_**’). This option cannot be followed: the corresponding vein is clearly concave in species of *Metallyticus*, *Chaeteessa*, and *Mantoida*, just as in Blattodea, Plecoptera, etc. In a similar line, we noticed a pair of inconsistent statements: **CuP** is said to occupy the claval furrow (‘**CuP** vein (claval furrow)’) in the diagnosis of *Santanmantis* Grimaldi 2003: p. 27), while the description of *Santanmantis axelrodi* Grimaldi, 2003 includes the statement ‘claval furrow at **CuA_2_**’ (Grimaldi 2003: p. 31). Confusion is finally complemented by the alternative recourse to the labels ‘**CuA**,’ ‘**Cu1**,’ and ‘**CuA_1_**’ to indicate the very same set of branches, and to the labels ‘**1V**,’ ‘**V_1_**’ and ‘**A_1_**’ for the very same vein, all on figs 23 *partim* and 24, representing forewings of *Santanmantis axelrodi*.

Among the more supported accounts, it is argued that vein* belongs to **R**, the fork observed in the *Mantis*-type representing the ‘ancestral’ **RA-RP** fork (Ragge 1955, Smart 1956, Ramsay 1990). Under this scenario vein* has no counterpart in the *MCM*-type, which then possesses a Blattodea-like, single-stemmed **R** (see Guo et al. 2013). A more elaborate proposition was made by Béthoux and Wieland (2009), assuming that vein* is the anterior branch of vein (2), as observed in the *MCM*-type, translocated onto vein (1). This hypothesis was strongly supported by the documentation of intermediate conditions, including data on intraspecific variability. Moreover, the occurrence of vein translocation can now be considered a routine transformation in insect wing evolution (Béthoux 2007, 2012, Cui et al. 2015). This scenario is also consistent with the current hypotheses on the position of species of *Metallyticus*, *Chaeteessa*, and *Mantoida* as successive sister-group of the remaining mantises: the translocation would be a derived condition of mantises of the *Mantis*-type.

A comparison of the fore- and hindwing venation patterns provides further evidence that a common stem **RP** + **MA** occurs in the forewing of the *MCM*-type. The hindwing venation of mantises generally conforms to that of cockroaches, including the occurrence of **arc**. The identification of **RA**, **RP**, **M**, and **CuA** is then straightforward: **R** and **M** run alongside until **M** and then **RP**, or **RP** + **M**, diverge (intraspecific variability can affect this area; Ramsay 1990); and **M** and **CuA** are connected via **arc**. Following the THCs herein favored, the number of terminal branches matches between fore- and hindwings; in all cases M is simple, or with a very distal fork (in *Metallyticus*; a branched **M** can be occasionally or usually present in forewings conforming to the *Mantis*-type); in all cases **RP** is simple or with a very distal fork. Finally, in *Chaeteessa* and *Mantoida* (Figs 4, 5), there is a point where **RP** sharply diverges from **RA**, this generating a broadening of the **RA**-**RP** area. This point, and the resulting broadened area, is present in both wing pairs at nearly the same level. The inflexion of the course of **RP** in the hindwing of *Mantis religiosa* (Linné, 1758) is mirrored by the point of divergence of **RP** in the forewing (Fig. 5).

**The wing posterior area**

Some authors have referred to particular areas of the wing according to the veins delimiting them (e.g., ‘cubital area’). In other cases, function-based names have been proposed, such as ‘vannus,’ a term coined by Snodgrass (1935) for veins associated with the third axillary sclerite, but semantically referring to the fan-like folding mechanism of the corresponding area in the hindwing. At the level of Pterygota, these two types of referring options have led to a great deal of confusion regarding, in the hindwing, the area posterior to the cubital furrow (**cf**). Several authors seem to have conflated it with **CuP** and/or with the hinge located posterior to **AA1** (herein ‘**ppa**’) into a ‘vena dividens’ (e.g. Séguy 1957). The various terminologies have been discussed to some extent by previous scholars (i.e., Hamilton 1972, Hennig 1981), but some aspects require further reconsideration.

The term ‘jugum’ was originally coined by Comstock (1893, 1918; among other publications by this author) for a particular posterior outgrowth of the forewing occurring in some Lepidoptera. According to Comstock, this finger-like outgrowth is supported by a vein belonging to the anal system. Martynov (1925) proposed to use the term in a much wider sense, viz. to refer to portions of foldable areas typical of neopteran insects (‘jugum’ appears to be a synonymy of his ‘neala’). Note that he recognized a set of ‘jugo-radial’ veins in Orthoptera (Martynov 1925: figs 22, 23). As for Mantodea, he identified a jugum in the forewing (Martynov 1925: fig. 24), anteriorly delimited by his ano-jugal fold (herein ‘plica prima anterior,’ **ppa**; this fold acts as a convex hinge). Notably, according to him, this jugum is filled with anal veins.

As mentioned above, Kukalová-Peck (1991) identified a distinct set of jugal veins, the occurrence of which has been confirmed in Palaeozoic palaeopteran insects only (Béthoux and Nel 2003, Béthoux et al. 2007, Béthoux 2015). As a consequence, the ‘jugum’ *sensu* Martynov (1925) is an area filled with anal veins and characteristic of Neoptera, while ‘jugal veins’ *sensu* Kukalová-Peck (1991) seem to occur only in stem-lineages of palaeopteran insects. This situation is problematic. Given that ‘jugum’ *sensu* Comstock (1893) initially had a much more restricted sense and that it is now used in a much wider sense (in relation to a vein system), we suggest that the structure highlighted by Comstock should be termed differently, and that ‘Juga’ should be strictly restricted to the vein system posterior to the ‘Analis’ system.

The whole area in the hindwing posterior to the cubital furrow (**cf)** has also been termed ‘vannus’ (Snodgrass 1935), a term which is a direct reference to the fan-like folding mechanism present in many hindwings of polyneopteran insects. However this is not always the case: for example, in *Metallyticus* spp., only the posterior-most portion of this area folds in a fan-like manner. Therefore ‘vannus’ (and the associated ‘vannal veins’) is not suitable for a general, standard terminology. In this context and regardless of the issues with the term ‘vannus’ itself, it is important to discuss here propositions made by Wootton (1979) for a standardized terminology for the corresponding area at the level of insects. Posterior to the **cf**, Wootton (1979) proposed to distinguish the clavus and the jugum in the forewing (the former is characterized by opposition to the characteristic trait of the vannus, viz. folding capacity) and the vannus and the jugum in the hindwing. In other words, the clavus and the vannus are delimited by the same folds, with the vannus possessing the additional ‘vannal fold.’ The secondary acquisition of an extended membranous area posterior to **ppa** in the *Mantis*-type forewing, a very probable analogue of the early development of the hindwing posterior area in polyneopteran insects, challenges Wootton’s (1979) proposition: in the *Mantis*-type forewings both a clavus and a vannus occur. It demonstrates that Wootton’s (1979) ‘vannal fold’ in the hindwing is actually homologous to his ‘jugal fold’ in the forewing (in addition to their position relative to **cf**, both act as a convex hinge).

The area posterior to **cf** has also been termed in reference to the vein systems supposedly filling it, such as ‘fan-like anal field’ (Brongniart 1893), or ‘anojugal lobe’ (Haas and Kukalová-Peck 2001, Kukalová-Peck and Lawrence 2004; ‘partial’ in the case of Dictyoptera). However, there is some uncertainty in the nature of the corresponding veins. According to Haas and Kukalová-Peck (2001: fig. 13) it is essentially filled with **AP** and jugal veins, but there is no evident rationale for such interpretation. Yet it remains possible that ‘anal area’ will prove inadequate at some stage.

This is leading us to coin three new terms for the area posterior to the **cf**, presumably applicable to polyneopteran insects, at least. This area is divided into three parts. The anterior part is delimited by **cf** and **ppa**. It will be referred to as ‘preplicatum’ (a derivate of ‘plicatum,’ defined below). The medial part is anteriorly delimited by **ppa** and posteriorly delimited by another fold, acting as a concave hinge, herein termed ‘plica prima posterior’ (**ppp**; beyond this fold the wing is attached to the thorax). We propose to refer to this area as the ‘plicatum.’ This proposition has the substantial advantage of avoiding reference to the nature of the veins filling the corresponding area and the inner folding mechanism of the area (if applicable). Derived from ‘plicatum,’ we propose to refer to the area anteriorly delimited by **ppp** and posteriorly attached to the thorax, smaller than the plicatum, as ‘plicatulum.’ Although a wider comparative analysis would be necessary for a positive statement, our plicatulum seems to be homologous to Martynov’s (1925) jugum. The plicatum can fold in a fan-like manner if it possesses ‘vannal folds.’ The plicatum, plicatulum, and the associated folds (**ppa** and **ppp**) constitute the essential structures allowing the neopterous condition. Finally, we propose to coin the term ‘remigulum’ (a diminutive of ‘remigium,’ largely used for the whole area posteriorly delimited by **cf**) for the whole area anteriorly delimited by **cf**. In other words, it encompasses the preplicatum, plicatum, and plicatulum.

‘**Pcu & 1V’ vs. ‘AA1 & Iaa_1_-aa_2_’ in hindwings of *Chaeteessa* spp.**

The existence of a ‘**Pcu**’ vein distinct from **CuP** and the anal system was proposed by Snodgrass (1935) based on wings of the American cockroach. However, our observation of wings of this species and additional cockroaches led us to concur with Kukalová-Peck, (1991): there are no structures interpretable as a vein distinct from **CuP** and **AA1** in cockroaches; **CuP** is always simple, **AA1** is simple or with early, vanishing side branches, and **AA2** is generally branched. It should be emphasized here that ‘**Pcu**’ has never been documented in palaeopteran groups, Plecoptera, or Orthoptera (fossil or extant).

As for mantises, a ‘**Pcu**’ was mentioned by Smart (1956) and Grimaldi (2003) (the latter contribution is dealt with above). Converting Smart’s ‘**Pcu**’ into **AA1** only partly solves the contention. In the hindwing of *Chaeteessa valida*, the first vein-like structure posterior to ‘**Pcu**,’ simple, was regarded by Smart as ‘**1V**,’ homologous to the vein we consider as **AA1** in cockroaches. In other words, with respect to the pattern observed in Blattodea or Plecoptera, there is a supernumerary vein-like structure between (our) **AA1** (simple) and **AA2** (branched) in hindwings of *C. valida* [Smart also reports the occurrence of distinct **1V** (simple), **Pcu** (simple), and **2V** (branched) in *Metallyticus*, but we failed to observe two simple, parallel veins representing ‘**1V**’ and ‘**Pcu**’ in this taxon]. Adding a supernumerary, hypothetical ‘**PCu**,’ lost in ‘higher mantises’ is the option favored by Smart (1956). Under this scenario, *Chaeteessa* spp. exhibits a plesiomorphic condition with respect to all other extant mantises.

We propose a different interpretation of the vein-like structure located between (our) **AA1** and **AA2**. As in Blattodea and Plecoptera, **ppa** is located between **AA1** and **AA2** in mantises. In these insects, as in Phasmatodea (among others), is it very often the case that in the hindwing plicatum, near the posterior wing margin, intercalary veins form along vannal folds. For most of its length this structure does not reach the level of sclerotization observed in the surrounding veins, and it never forms a complete tube. Instead, it is formed of isolated patches of sclerotization, like cross-veins in the plicatum. Moreover, its origin is very faint. All of these features demonstrate that Smart’s ‘**1V**’ is an intercalary vein (herein ‘**Iaa_1_-aa_2_**’). Among mantises, possessing an intercalary vein along **ppa** is unique to *Chaeteessa*, and clearly is a derived condition.

Ongoing research has demonstrated that wing venation THCs proposed by Béthoux and Wieland (2009) and followed herein might be reconsidered. A new publication on the topic is expected.

### 3.3. Pro-, meso-, and metathoracic legs

**Figure 6.**
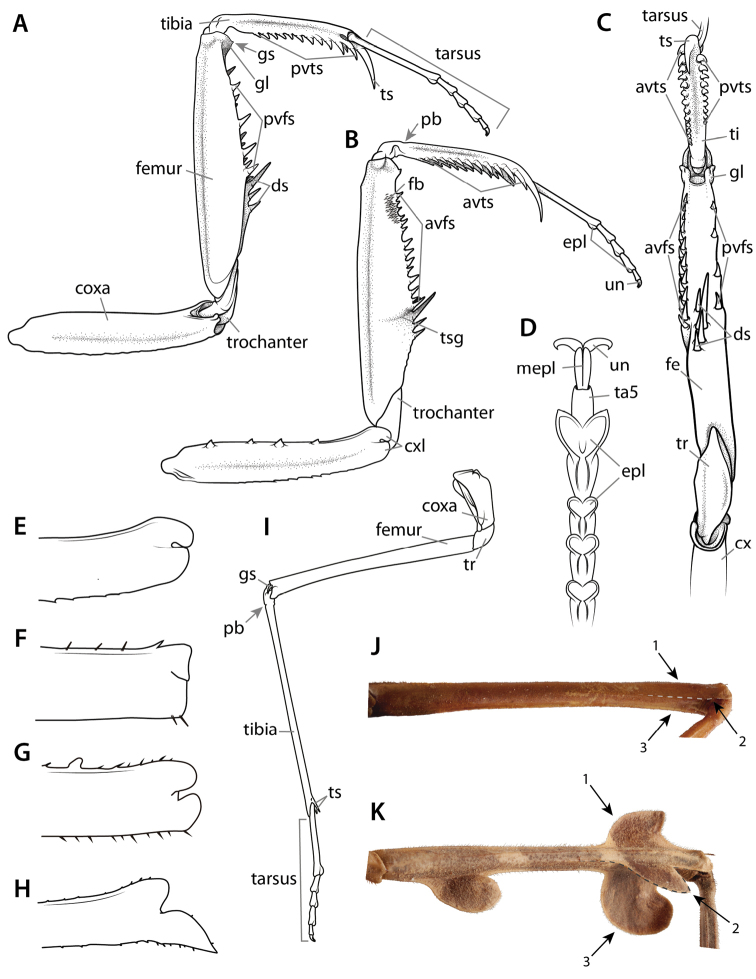
Annotated illustrations of leg structures. Generalized mantodean foreleg **A** dorsal view; **B** ventral view; **C** interior view. Generalized tarsus **D** ventral view. Examples of convergent coxal lobes: **E** *Sphodromantis* sp. ♂; **F** *Paramorphoscelis gondokorensis* Werner, 1907 ♀. Example of parallel coxal lobes **G** *Acromantis insularis* Giglio-Tos, 1915 ♀. Example of divergent coxal lobes **H** *Gongylus gongylodes* Linné, 1758 ♂. Generalized metathoracic leg **I** ventral view. Examples of carinae that can be present on the meso- and metathoracic femora and tibiae **J** *Stagmatoptera* Burmeister, 1838 ♀: ventral view **1** = anterodorsal metafemoral carina; **2** = anteroventral metafemoral carina; **3** = posteroventral metafemoral carina. Example of the cuticular lobes that can project from leg carinae **K** *Alangularis multilobata* (Chopard, 1910) ♀: ventral view **1** = anterodorsal metafemoral lobe; **2** = anteroventral metafemoral lobe; **3** = posteroventral metafemoral lobe. Dashed lines demarcate anteroventral carinae (**J**) and associated lobes (**K**). Abbreviations: **avfs** = anteroventral femoral spines; **avts** = anteroventral tibial spines; **cx** = coxa; **cxl** = coxal lobes; **ds** = discoidal spines; **epl** = euplantulae; **fb** = femoral brush; **fe** = femur; **gl** = genicular lobe; **gs** = genicular spur; **mepl** = medial euplantula; **pb** = proximal bend in the tibia; **pvfs** = posteroventral femoral spines; **pvts** = posteroventral tibial spines; **ta5** = tarsomere 5; **ti** = tibia; **tr** = trochanter; **ts** = tibial spur; **tsg** = tibial spur groove; **un** = unguis.

Praying mantises have three pairs of legs each consisting of a coxa, trochanter, femur, tibia, and tarsus (Fig. 6). The tarsus is subdivided into 5 tarsomeres, the exception being species in the genus *Heteronutarsus* Lefebvre, 1835, in which tarsomeres may be fused, thus resulting in apparently fewer segments (Wieland 2013: p. 161) or in specimens who have lost limbs early in their development (Roy 1999). In order to clearly associate each segment to its corresponding leg and to dispel confusion from variable use in past literature, it is recommended to use the following prefixes: fore-, meso-, and meta- (e.g., forefemur, mesofemur, metafemur). The mesothoracic and metathoracic legs of the praying mantis are fairly typical for insects, the exception being cuticular expansions (lobes) that originate from keels present in the leg segments of some genera (Wieland 2013: p. 96, Svenson et al. 2015) (Fig. 6j–k). The raptorial forelegs are complex structures variably armed with spines, crenulations, tubercles, and setae (see Suppl. material 4: Leg structure terminology).

**Foreleg spination formula**

Foreleg spines constitute an important character system in Mantodea taxonomy. Taxonomic description normally includes a verbal description detailing the number and relative arrangement of such spines. Rivera (2010) presented a more concise method to report spination formulas in his description of the genus *Chromatophotina* Rivera, 2010. The rationale behind it is to use the spination formula to present substantial information contained in the forelegs in a compact form, thereby minimizing repetitive wording and enabling users to extract and process information straightforwardly. The system conceived by Rivera (2010) is reminiscent of those implemented for other organisms, such as the floral formula (Prenner et al. 2010) or the mammal dental formula (Martin et al. 2001) and can be applied for descriptive purposes at different hierarchical levels (e.g., species, tribe, family). Since its inception, the spination formula has been used only in a few subsequent publications (e.g., Rivera et al. 2011, Ippolito and Lombardo 2012, Lombardo et al. 2013, Brannoch and Svenson 2016a, 2016b). In its most recent usage, Brannoch and Svenson (2016a, 2016b) updated the formula by incorporating the morphological nomenclature of Wieland (2013) (see Foreleg annotation). Here we recommend the use of the foreleg spination formula as modified by Brannoch and Svenson (2016a, 2016b) to describe the number and arrangement of the foreleg spines. We provide the formula, as well as a discussion, of particular taxonomic groups where foreleg spination is confounding.

**Foreleg annotation**

Several different terminologies have been used in the descriptions of spination patterns, (e.g., “internal” and “external” spines, “inner” and “outer” spines, “ventral” and “dorsal” spines). Wieland (2013) applied a strictly morphological approach by naming the rows of spines with respect to their position on the legs, under the assumption that the forelegs are regarded as typical insect walking legs – i.e., stretched aside. Under this premise, the rows of spines lie on the edges of the ventral surface of forefemur and foretibia, respectively. They are correspondingly named anteroventral (formerly “inner” or “internal”) and posteroventral (formerly “outer” or “external”) rows of foretibial and forefemoral spines. The discoidal spines are positioned in a single oblique row on the ventral surface of the forefemur and therefore a special morphological assignment is unnecessary. The spination formula details the number of spines on the forelegs including numeric variation. The spines on the forefemur (F) and the foretibia (T) are presented as respective series separated by a semicolon (;). The numeric values for the discoidal spines (**DS**), the anteroventral spines (**AvS**), and posteroventral spines (**PvS**) are listed in that order for the forefemur; only the latter two are given for the foretibia. The formula includes neither the genicular spines of the forefemora nor the tibial spur (which, morphologically, is a derivative of the distal-most anteroventral foretibial spine).

The following example describes the foreleg spination of a fictive species:

F = 4DS/10–12AvS/4–5PvS; T = 7–8AvS/12–14PvS

This example formulation indicates that this fictive species exhibits 4 discoidal spines. The forefemora carry 10–12 anteroventral spines (thus highlighting observed variation) and 4–5 posteroventral spines (again highlighting observed variation). The foretibiae carry 7–8 anteroventral and 12–14 posteroventral spines.

Sometimes a unique specimen is all that is available (e.g., a holotype) and it might exhibit bilateral asymmetry in the number of spines. When this situation arises, such as when describing a new species based on a single specimen, it is important to make a clear distinction to be as precise as possible. In this case, an additional annotation of “R” (right) or “L” (left) can be added to the number of spines detailed in the spination formula. The following example illustrates this situation:

F = 4DS/10(R)–11(L)AvS/4PvS; T = 7AvS/12(R)–14(L)PvS

This means that this unique specimen exhibits 4 discoidal spines, the right forefemur features 10 anteroventral spines, the left forefemur features 11 anteroventral spines, with 4 posteroventral spines on each forefemora. Similarly, both foretibiae carry 7 anteroventral spines, while 12 and 14 posteroventral spines occur on the right and left foretibia, respectively.

In the form presented above, this formula can be used for the description of the vast majority of forelegs across all mantodean species described so far. There are only a few exceptions with highly modified foreleg morphologies, some of which call for special addenda to the formula. In some species, spines can be reduced up to a state where they can only be detected through scanning electron microscopy (SEM) (Wieland 2013). Such spines that cannot be seen with a stereo-microscope are not considered in the formula.

**Figure 7.**
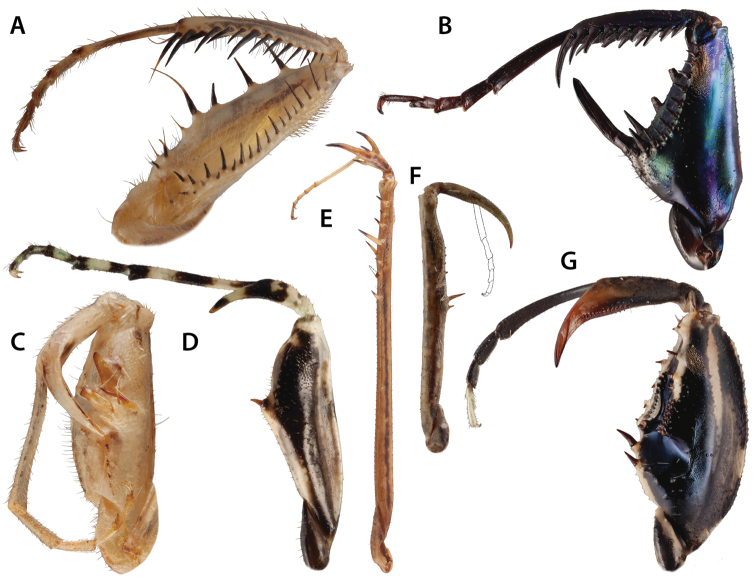
Figure 7. Foreleg spination variation across Mantodea presented in anteroventral view. **A** *Chaeteessa* sp. ♂ **B** *Metallyticus splendidus* ♀ **C** *Paramorphoscelis gondokorensis* ♀ **D** *Amorphoscelis pulchella* Giglio-Tos, 1914 ♀ **E** *Thesprotia* sp. ♂ **F** *Compsothespis* sp. ♀ **G** *Paraoxypilus* sp. ♀. Dashed outlines indicate relative position of broken foreleg structures (i.e., spine **E** and tarsus **F**). Forelegs not to scale.

As far as our knowledge allows, the following are the unique cases that we have identified where the spination formula will need special modifications:

**1.) *Chaeteessa* Burmeister, 1838 (Fig. 7a)**

This enigmatic Neotropical genus is the sister-group of all remaining extant Mantodea according to Svenson and Whiting (2009) and Wieland (2013). Its foreleg features a full set of spines, studied in detail by Wieland (2013). However, the overall morphology in this genus is slightly atypical. The foretibia is strongly bent laterad. The tibial spur has primarily been assumed to be missing, as can be inferred, for instance, from Beier (1968) and other publications. However, Roy (1999) mentioned a small, distal spine on the tip of the foretibia, a “setigerous tubercle,” in the place of the tibial spur. Klass and Meier (2006) tentatively hypothesized a secondary reduction of the tibial spur in *Chaeteessa*, which was further corroborated by Wieland (2013). Due to the shape of the foretibia, the tibial spur was possibly reduced and the distal anteroventral and the posteroventral foretibial spines adopted the function of the former spur (Wieland 2013). Correspondingly, the latter spines are strongly elongated and rest within two spur grooves (an anterior and a posterior groove) instead of within one as in all remaining Mantodea.

The morphological assignment of several spines is still uncertain. Distally on the forefemur, there is a large spine that may be an enlarged genicular spine (interpreted as such in Wieland 2013) or an additional posteroventral spine. There are four distinct forefemoral posteroventral spines as well as three additional proximal strong setae that might represent reduced posteroventral spines. Wieland (2013) encoded the number of forefemoral posteroventral spines in *Chaeteessa* as “more than 6.” It is currently unknown if there are actually 7 posteroventral spines or less.

The arrangement of anteroventral spines in the proximal region of the forefemur, as well as the number of discoidal spines, are also inconclusive. The row of anteroventral spines splits proximally into two diverging rows. This character has also been observed in other Mantodea, e.g., *Mantoida* and *Acanthops* (see Wieland 2013: p. 73). Proximal to the split row of anteroventral femoral spines lies the anterior spur groove for the enlarged distal anteroventral foretibial spine. Proximal to this claw groove, (between the groove and the femoro-trochanteral joint) is an additional spine. There are two distinct discoidal spines on the ventral surface of the forefemur. The assignment of the single most proximal spine mentioned above is uncertain. It might be a discoidal spine (as the spur groove lies between the anteroventral spines and the single spine, a position typically found in Mantodea). However, considering the atypical morphology of the foreleg, the spur groove might have shifted distad from its original position when the foretibial morphology changed. Studies focusing on postembryonic changes in the conformation and arrangement of spines or paleontological discoveries might provide the necessary insights to fully resolve the spination formula for Chaeteessidae. In the meantime, we provide a tentative spination formula as follows:

F = 2(3?)DS/19(20?)AvS/7(?)PvS; T = 14AvS/4PvS

**2.) *Metallyticus* Westwood, 1835 (Fig. 7b)**

Wieland (2008, 2013) studied the foreleg morphology of *Metallyticus*. In this extraordinary Southeast Asian genus, the proximal posteroventral forefemoral spine is enormously enlarged, which is apomorphic for the genus. In the literature, the discoidal spines have been noted to be (primarily) missing (e.g., Roy 1999). However, the study of first instar specimens with SEM imaging led to the conclusion that the adult morphology is highly derived, probably due to the specialized lifestyle of the genus (Wieland 2013). The first instar shows the “typical” mantodean foreleg morphology, which undergoes considerable changes during postembryonic development (Wieland 2013). Actually, the proximal anteroventral forefemoral spine is a persisting discoidal spine that lost its function (which was taken over by the elongated posteroventral spine) (Wieland 2013). The spination formula for *Metallyticus* therefore is:

F = 1DS/11AvS/4PvS; T = 8AvS/6PvS

**3.) Perlamantinae (Fig. 7c)**

*Perlamantis* Guérin-Méneville, 1843 and *Paramorphoscelis* Werner, 1907, the only two genera of this subfamily, have highly specialized forelegs. *Perlamantis allibertii* was studied by Wieland (2013: pp. 68, 74) and SEM images were presented. Except for microscopic remnants of spines, there are no spines on the foretibia. The remnants are larger than the ‘typical’ reduced spines but they are hard to recognize under a stereo-microscope. Therefore, they are not considered in the spination formula. The forefemur lacks posteroventral spines (except for microscopic remnants). One discoidal spine is present. The four remaining anteroventral forefemoral spines are strongly modified, most of them forming flattened and enlarged plates that are oriented in oblique angles towards each other. The morphological assignment of the spines in *Perlamantis* is unequivocal, the spination formula therefore reads as follows:

F = 1DS/4AvS/0PvS; T = 0AvS/0PvS

**4.) Amorphoscelinae (Fig. 7d)**

Foreleg morphology in Amorphoscelinae (e.g., *Amorphoscelis* Stål, 1871, *Gigliotoscelis* Roy, 1973, *Caudatoscelis* Roy, 1973) is unique because all spines except for the tibial spur and one discoidal spine are reduced. Wieland (2013) presented SEM images of an unidentified *Amorphoscelis* species from Southeast Asia, showing that remnants of spines from both femoral rows and distal discoidal spines are present with no remnant spines found on the foretibia. As these remnants are only discernible under the SEM they are not considered in the spination formula.

F = 1DS/0AvS/0PvS; T = 0AvS/0PvS

**5.) Thespidae and Haaniinae (Fig. 7e)**

Members of Neotropical and Nearctic Thespidae and Asian Haaniinae exhibit atypical foreleg morphology, where both forefemora and foretibiae have undergone extensive modifications resulting in a remarkable diversity of spination patterns. Despite this, the spination formula can accommodate most of this variation without resorting to additional symbology or annotations. Therefore, members of *Thespis* Audinet-Serville, 1831 exhibit the following spination formula:

F= 4DS/12–13AvS/4PvS; T=8–11AvS/5PvS

However, special annotation is needed for taxa such as *Bantia* Stål, 1877, *Mantillica* Westwood, 1889, *Pseudomusonia* Werner, 1909, *Thesprotia* Stål, 1877, *Thesprotiella* Giglio-Tos, 1915, *Thrinaconyx* Saussure, 1892, and other related genera. In these genera, one or two of the distal-most spines of the anteroventral foretibial series have shifted from their usual ventral location into either a slightly lateral or decidedly dorsal position (e.g., Wieland 2013, Agudelo and Rafael 2014). The following example, corresponding to *Bantia*, shows how to account for dorso-laterally displaced spines in the spination formula:

F=4DS/9–10AvS/4PvS; T=4–6[+1]AvS/4–7PvS

Notice that the foretibial **AvS** exhibit the following count: 4–6[+1]. This means that this part of the foretibia exhibits 4 to 6 spines in the normal, ventral position, whereas the distal-most spine (i.e., [+1]) is displaced from the continuity of the series into a more dorso-lateral location. Therefore, the full foretibial **AvS** series in *Bantia* includes 5 to 7 spines as a whole. It is worth mentioning that the number of displaced distal **AvS** is fixed for each genus, whereas the remaining **AvS** can (and do) exhibit variation in number. Therefore, the annotation [+1] for *Bantia* will remain constant for all of its members.

Another, more extreme example of this kind of annotation, is represented by members of *Thesprotia* (e.g., Wieland 2013: pp. 63, 83), where the spination formula would be:

F=3DS/5–6AvS/1PvS; T=[+2]AvS/1PvS

In this example, the foretibial **AvS** series is reduced to simply [+2], because there are no ventral spines and the only two spines of this series are displaced into a distinctly dorsal position. As in *Bantia*, the [+2] annotation will remain constant across all species within this genus.

In some taxa, the displacement of the distal-most foretibial **AvS** is not evident. For example, there has been some controversy on the location of this spine in *Galapagia solitaria* Scudder, 1893 (see Hebard 1920), where the distal-most foretibial **AvS** seems to be only slightly dorso-laterally displaced. This question might be solved by further examining immature stages to better define the spination formula in this and other genera that may exhibit a similar condition.

**6.) *Compsothespis* Saussure, 1872 (Fig. 7f)**

The foreleg morphology of the African *Compsothespis* shows a strong tendency of reduction (Wieland 2013). The foretibia does not carry any spines except for the tibial spur. In the forefemur, all visible spines except two discoidal spines are very small but clearly discernible. There probably has been a reduction of anteroventral forefemoral spines as their number is unusually low. However, these putative remnants are not visible under a stereo-microscope and are thus not considered in the formula. Although strongly reduced in size, the morphological assignment of the spines is clear:

F = 3DS/4AvS/4PvS; T = 0AvS/0PvS

**7.) Paraoxypilinae (Fig. 7g)**

The Australian Paraoxypilinae (e.g., *Paraoxypilus* Saussure, 1870, *Gyromantis* Giglio-Tos, 1913, *Cliomantis* Giglio-Tos, 1913) have highly modified forelegs, possibly as an evolutionary response to hunting ants (Wieland 2013). The forelegs of an unidentified *Paraoxypilus* species were studied with the SEM by Wieland (2013: p. 69). Many of the forefemoral and all foretibial spines in this genus are small, blunt, and strongly structured with small cuticular ridges. There are three discoidal spines, the distal-most of which is very small but recognizable. Following a single larger proximal anteroventral forefemoral spine, the next nine anteroventral spines form a dense, comb-like structure that interacts with the similarly structured anteroventral foretibial spines that are positioned on a small, shelf-like protrusion on the distal anterior edge of the foretibia. While the set of spines is complete on the forefemur, there are only remnants of posteroventral foretibial spines, discernible only with SEM and therefore not considered. The morphological origins of the spines are unequivocal and the formula for *Paraoxypilus* (other Paraoxypilinae have to be studied in detail) is:

F = 3DS/13AvS/4PvS; T = 12AvS/0PvS

For most of the abovementioned taxa larger numbers of specimens have to be studied because the degree of variation is unknown so far. The examination of first instar specimens is likely to shed further light on the evolution of foreleg spination and uncertain cases of morphological assignment (e.g., Wieland 2013; Rivera and Svenson 2016).

**Verbal description of foreleg spines**

A good proportion of Mantodea taxa show variation in the way the spines are arranged. For instance, in certain thespids the foretibial **AvS** may exhibit spines of various sizes, and the size of the gaps between each spine of the series may also vary. All of this information is taxonomically relevant (e.g., Rivera and Svenson 2016). The spination formula cannot account for such variation without resorting to complex annotations, which will ultimately turn this tool into an overly complicated system and thus contrary to its intended use. Instead, authors are encouraged to complement the spination formula with those details of spine arrangement that they may consider, in their better judgment, relevant within the context of their study.

We suggest representing spine size arrangement by using the letter “I, i” and the underscore symbol, which has been used periodically in the taxonomic descriptions of Mantodea (e.g., Rehn 1935). The majuscule (i.e., “I”) represents relatively large spines, the minuscule (i.e., “i”) represents relatively smaller spines, and the underscore symbol (i.e., “_”) represents spineless regions. For example, in *Cornucollis masoalensis* Brannoch and Svenson, 2016 the forefemoral anteroventral spines alternate in size from medium to small in the following formation: IiIiIiIiI_I, with a spineless region between the distal penultimate and ultimate anteroventral spines (Brannoch and Svenson 2016a).

### 3.4. Thoracic structures

**Figure 8.**
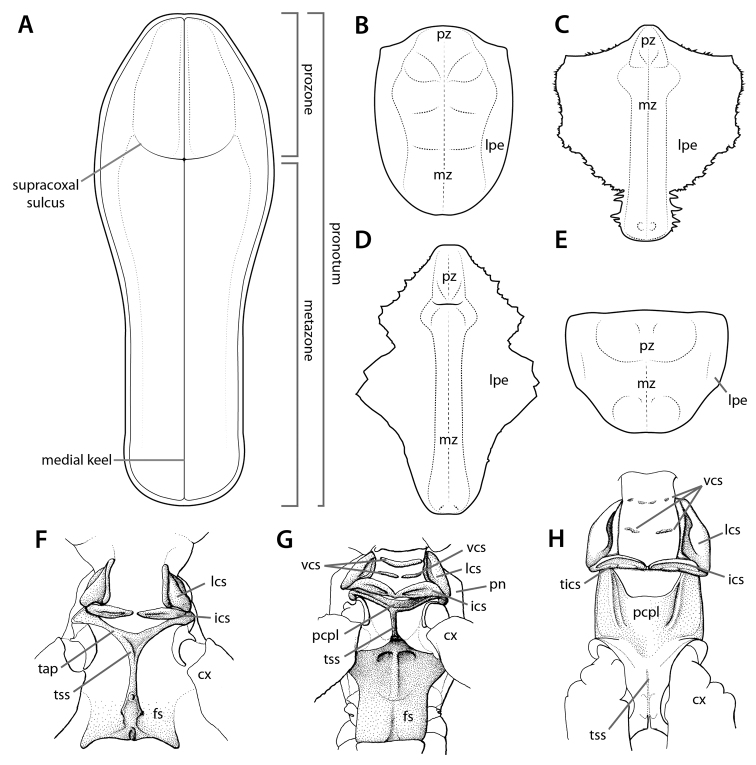
Annotated illustrations of thoracic structures. Pronotum in dorsal view **A** *Sphodromantis* sp. ♂ (illustration by Josh Maxwell) **B** *Theopompella congica* Rehn, 1949 ♀ **C** *Idolomantis diabolica* (Saussure, 1869) ♀ **D** *Deroplatys indica* Roy, 2007 ♂ **E** *Amorphoscelis pulchella* ♀. Prothorax in ventral view **F** *Mantoida maya* ♂ **G** *Humbertiella* Saussure, 1869 ♀ **H** *Phyllocrania paradoxa* ♂. Figures **F**–**H** reproduced and adapted from Wieland (2013). Structures not to scale. Abbreviations: **cx** = coxa; **fs** = furcasternite; **ics** = intercervical sclerite; **lcs** = lateral cervical sclerite; **lpe** = lateral pronotal expansion; **mz** = metazone; **pcpl** = postcervical plate; **pn** = pronotum; **pz** = prozone; **tap** = transverse anterior part of T-shaped sclerite; **tics** = torus intercervicalis; **tss** = T-shaped sclerite; **vcs** = ventral cervical sclerite.

**Figure 9.**
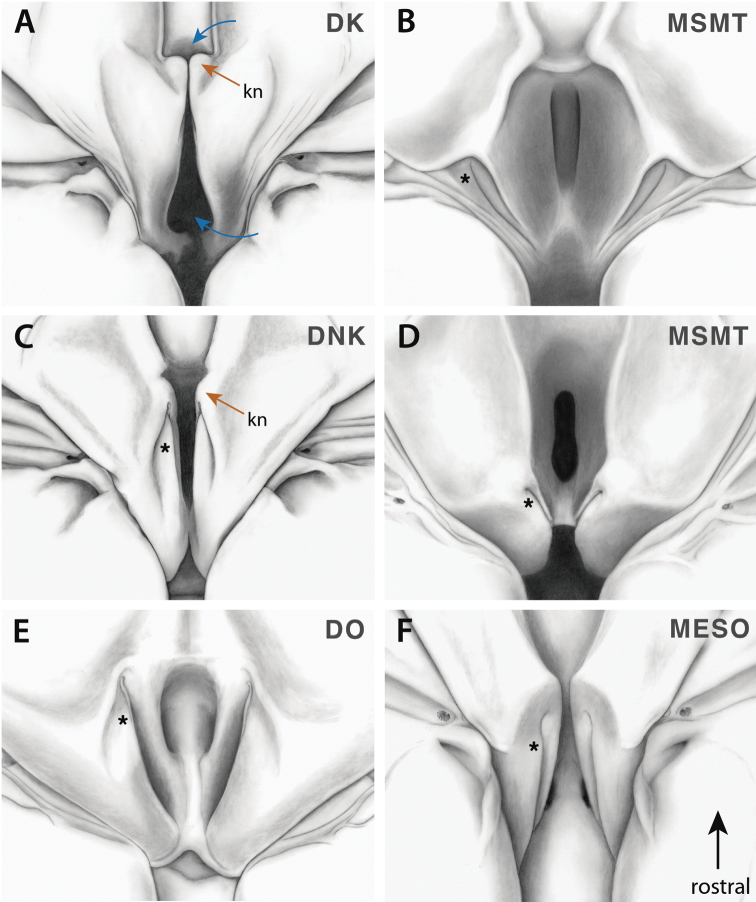
Annotated illustrations of hearing organ forms. Figures reproduced and adapted from Yager and Svenson (2008). **A** *Parasphendale agrionina* Gerstaecker, 1869 ♂ DK form hearing organ **B** *Mantoida* sp. ♀ MSMT form hearing organ **C** *Parasphendale agrionina* ♀ DNK form hearing organ **D** *Angela* Serville, 1839 ♂ MSMT form hearing organ **E** *Tarachodes afzelii* (Stål, 1871) ♀ DO form hearing organ **F** *Hymenopus coronatus* ♀ MESO form hearing organ. Asterisks indicate the ventral rod (**B**–**F**), which is not visible in the DK form (see Yager and Svenson 2008). Orange arrows indicate the knob, present on DK and DNK forms, barely visible in DO forms, and not present in MSMT forms. Blue arrows indicate opening to the auditory chamber. Abbreviation: **kn** = knob.

The thoracic structures of Mantodea include the pronotum (Fig. 8a–e), thoracic sternites, and the sclerites of the cervix (Fig. 8f–h), which lend stability to the region and are, in part, important insertion sites for the muscles of the neck (see Wieland 2006: 57) (see Suppl. material 5: Thoracic structure terminology). Some mantodean taxa feature lateral pronotal expansions that range from a minor marginal rim around the pronotum (Fig. 8a) to substantial lobes on the pronotal margin (Fig. 8b–e). These pronotal expansions can enhance the possessor mantis’ special resemblance to substrate or vegetation (e.g., dead leaf mantis *Phyllocrania paradoxa* Burmeister, 1838). Additional structures include the thoracic hearing organs (Fig. 9), of which there are six auditory types with four that are morphologically distinguishable (see Yager and Svenson 2008). While many mantodean taxa possess hearing organs, there are lineages of primitively earless mantises (e.g., Acanthopoidea, see Rivera and Svenson 2016).

### 3.5. Abdominal and genitalic structures

**Figure 10.**
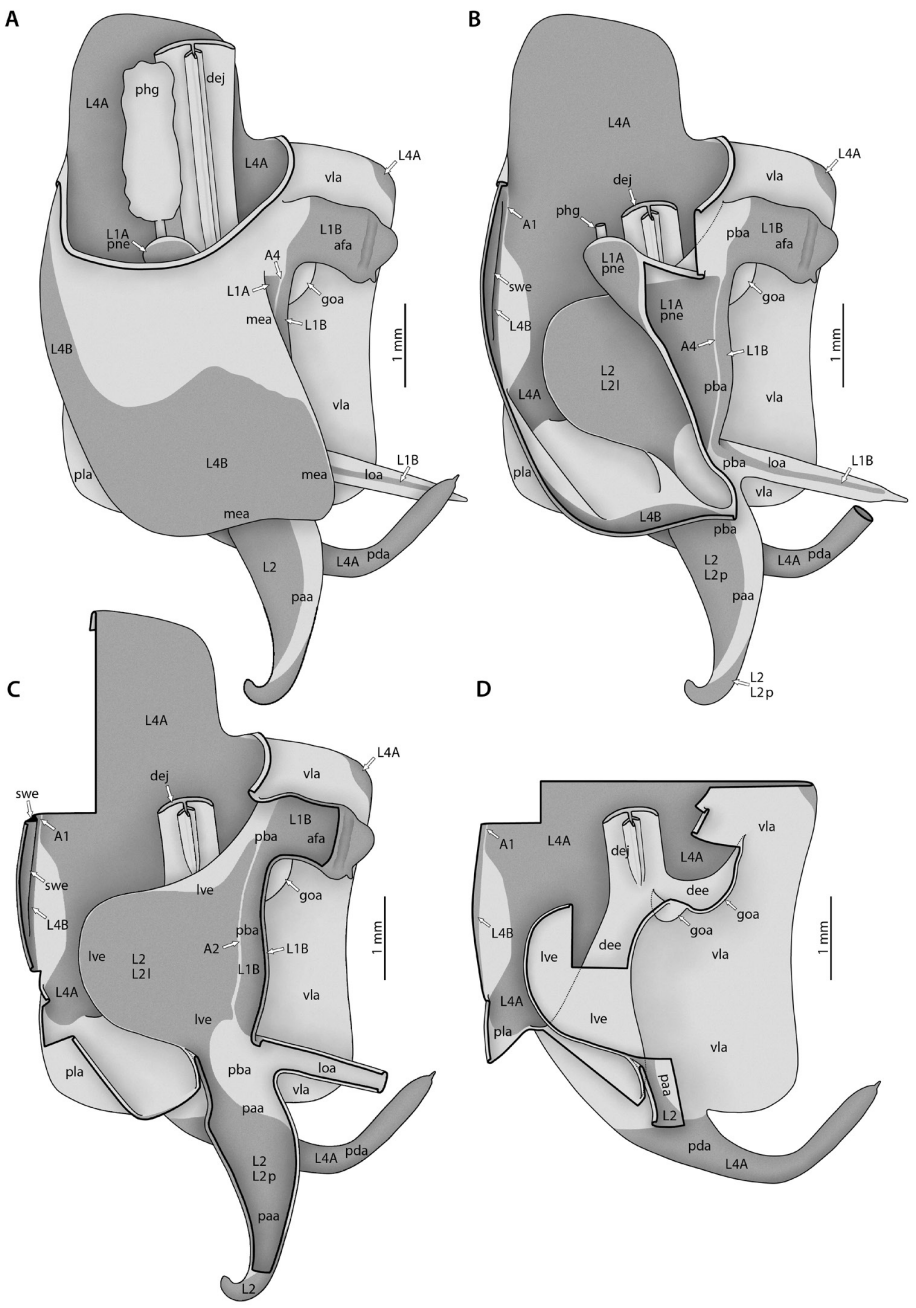
Annotated illustrations of male *Sphodromantis* sp. genitalia for morphological use. Male postabdomen, left phallic complex **A**–**D** dorsal view. From **A** (showing intact left complex) to **D** dorsal and peripheral parts are removed step by step. Thick black lines are (virtual) cutting lines. Continuous thin black lines are freely visible edges (= lines along which the cuticle bends away from the observer’s view). Dashed thin black lines are (parts of) edges hidden beneath other cuticle. Membranous cuticle in very light gray, sclerotized cuticle in darker gray; cuticle shaded darker where it goes underneath other cuticle. For abbreviations see text, glossary, or Suppl. material 2: Extended abdominal glossary.

**Figure 11.**
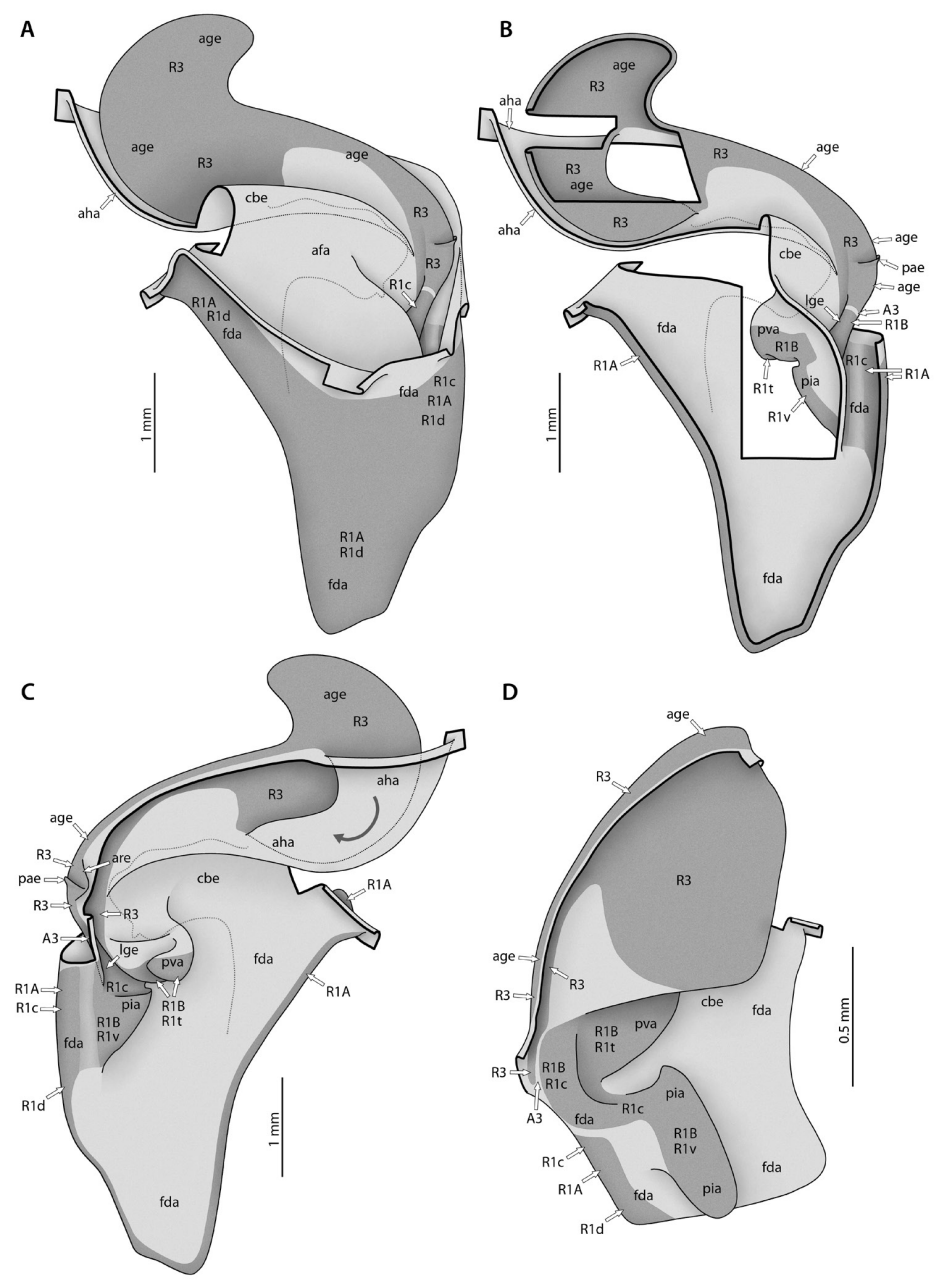
Annotated illustrations of the right phallomere in male genitalia for morphological use. **A**–**C** Male postabdomen, right phallomere of *Sphodromantis* sp. **A** dorsal view of intact right phallomere **B** dorsal view of right phallomere with many parts removed **C** ventral view of intact right phallomere. *Chaeteessa caudata* Saussure, 1871 right phallomere **D** ventral view, intact. Thick black lines are (virtual) cutting lines. Continuous thin black lines are freely visible edges (= lines along which the cuticle bends away from the observer’s view). Dashed thin black lines are (parts of) edges hidden beneath other cuticle. Dashed gray line indicating outline of process **afa** of left phallic complex as it is placed in association with the right phallomere. Membranous cuticle in very light gray, sclerotized cuticle in darker gray; cuticle shaded darker where it goes underneath other cuticle. For abbreviations see text, glossary, or Suppl. material 2: Extended abdominal glossary.

**Figure 12.**
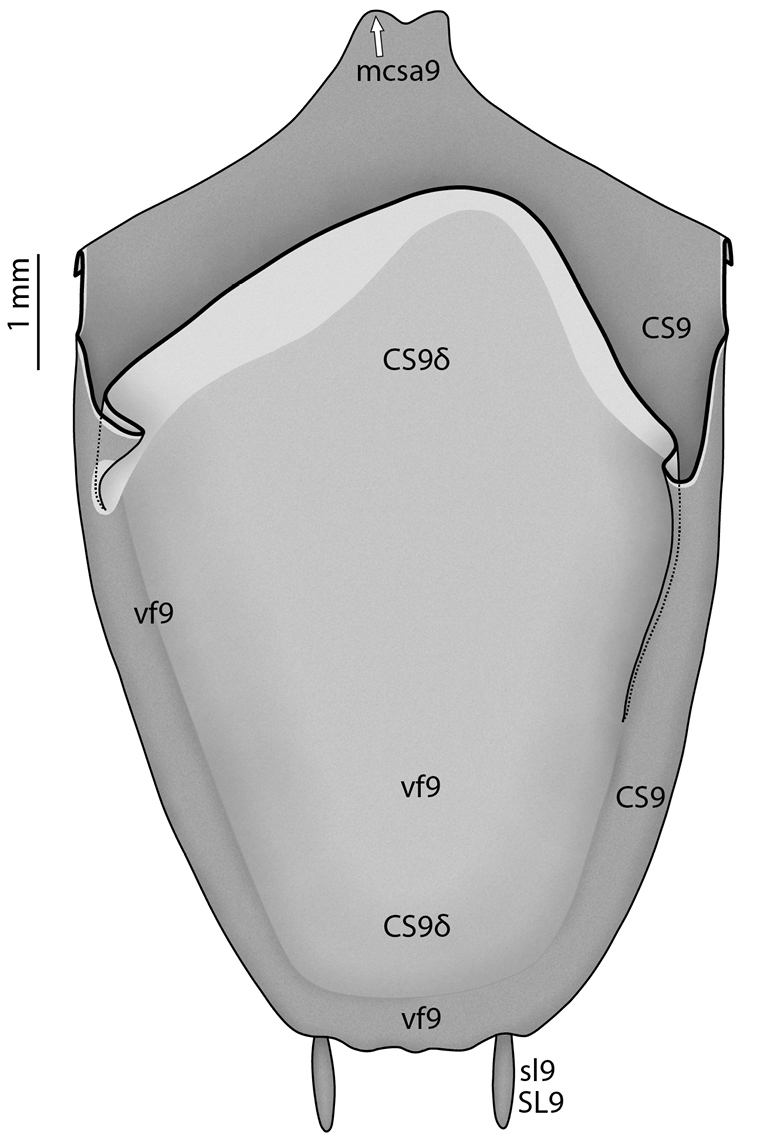
Annotated illustrations of the male *Sphodromantis* sp. postabdomen for morphological use. Coxosternite **CS9** (= subgenital plate) and ventral fold **vf9** (“subgenital lobe”) of *Sphodromantis* sp., dorsal view. Thick black lines are (virtual) cutting lines. Continuous thin black lines are freely visible edges (= lines along which the cuticle bends away from the observer’s view). Dashed thin black lines are (parts of) edges hidden beneath other cuticle. Membranous cuticle in very light grаy, sclerotized cuticle in medium to darker grаy (darker = more strongly sclerotized); cuticle shaded darker where it goes underneath other cuticle. For abbreviations see text, glossary, or Suppl. material 2: Extended abdominal glossary.

**Figure 13.**
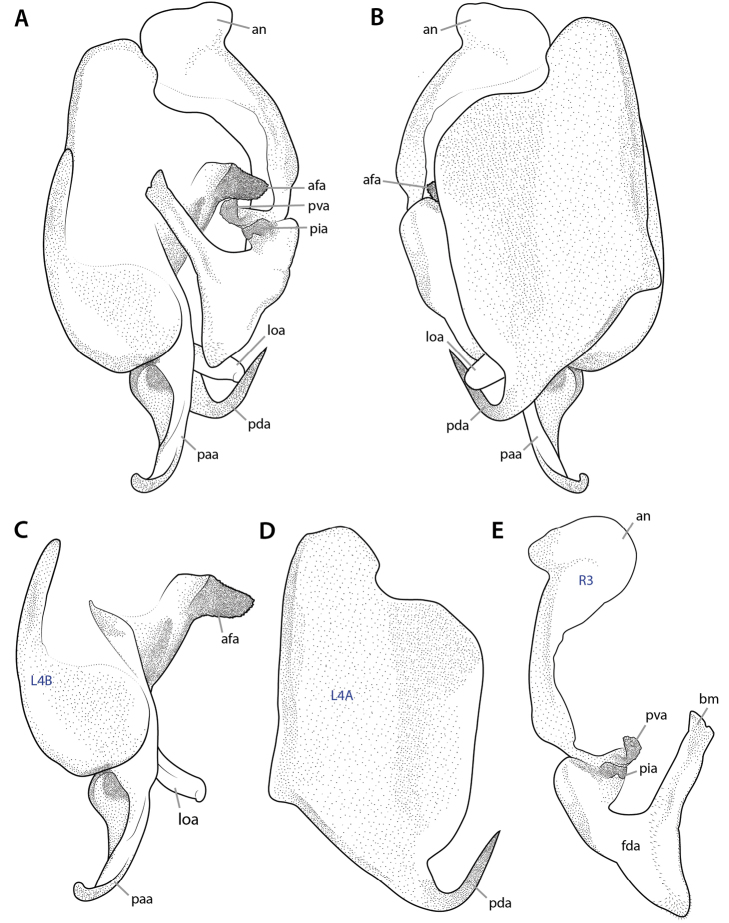
Annotated illustrations of *Sphodromantis* sp. male genitalia for taxonomic use. Intact genital complex **A** dorsal view **B** ventral view. Disarticulated genital complex to isolate the individual phallomeres **C**–**E**: **C** left phallomere (of the left phallic complex), dorsal view **D** ventral phallomere (of the left phallic complex), dorsal view **E** right phallomere, ventral view. Blue text refers to major sclerotizations. Abbreviations: **afa** = anterior process (left phallomere); **an** = anterior extension of sclerite R3 (anterior apodeme); **bm** = dextral extension (right phallomere); **fda** = main posterior lobe (right phallomere); **L4A** = sclerite extending over the ventral wall (left phallomere); **L4B** = sclerite extending over the dorsal wall (left phallomere); **loa** = posteromesal (left phallomere); **paa** = posterior process (left phallomere); **pda** = posterior process (ventral phallomere); **pia** = process posterolateral to pva (right phallomere); **pva** = process anteromesal to pia (right phallomere); **R3** = anteriorly extending sclerite (right phallomere).

**Figure 14.**
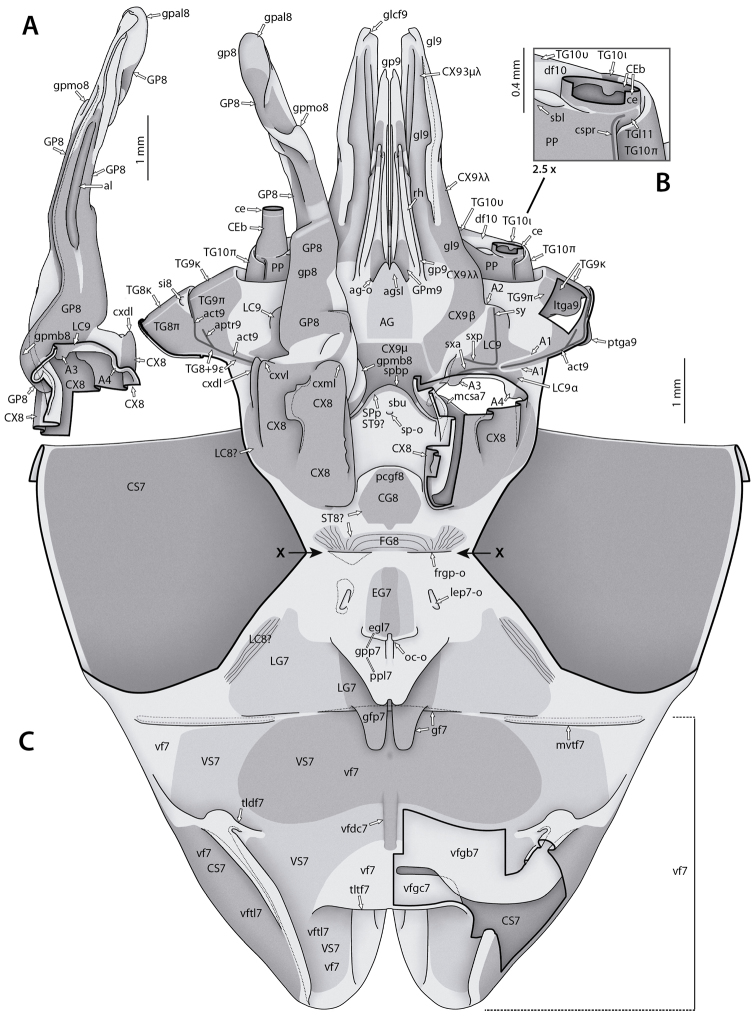
Annotated illustrations of the female *Sphodromantis* sp. postabdomen and genitalia for morphological use. **A** left gonapophysis 8 with adjoining parts of coxa 8 and laterocoxa 9 in dorsal view **B** lateral parts of terminal abdomen in ventral view, with focus on base of cercus (enlarged 2.5× from **C**) **C** entire genital region, most parts in external view. Posterior parts (segments VIII–XI) are bent 180° dorsally and to the anterior compared to anterior parts (of segment VII), along the axis marked by arrows X. The lower half of the illustration shows the ventral fold 7 (“subgenital lobe”) and the genital fold area in dorsal view; the upper half of the illustration shows the ventral sides of segments VIII–XI in ventral view. Left gonapophysis 8 removed. Thick black lines are (virtual) cutting lines. Continuous thin black lines are freely visible edges (= lines along which the cuticle bends away from the observer’s view). Dashed thin black lines are (parts of) edges hidden beneath other cuticle. Thick gray lines are internal ridges. Dashed gray line next to fold **mvtf7** in **C** giving outline of area where dorsal and ventral walls of ventral fold 7 are firmly connected by columellae. Membranous cuticle in very light gray, sclerotized cuticle in medium to darker gray (darker = more strongly sclerotized); cuticle shaded darker where it goes underneath other cuticle. For abbreviations see text, glossary, or Suppl. material 2: Extended abdominal glossary.

**Figure 15.**
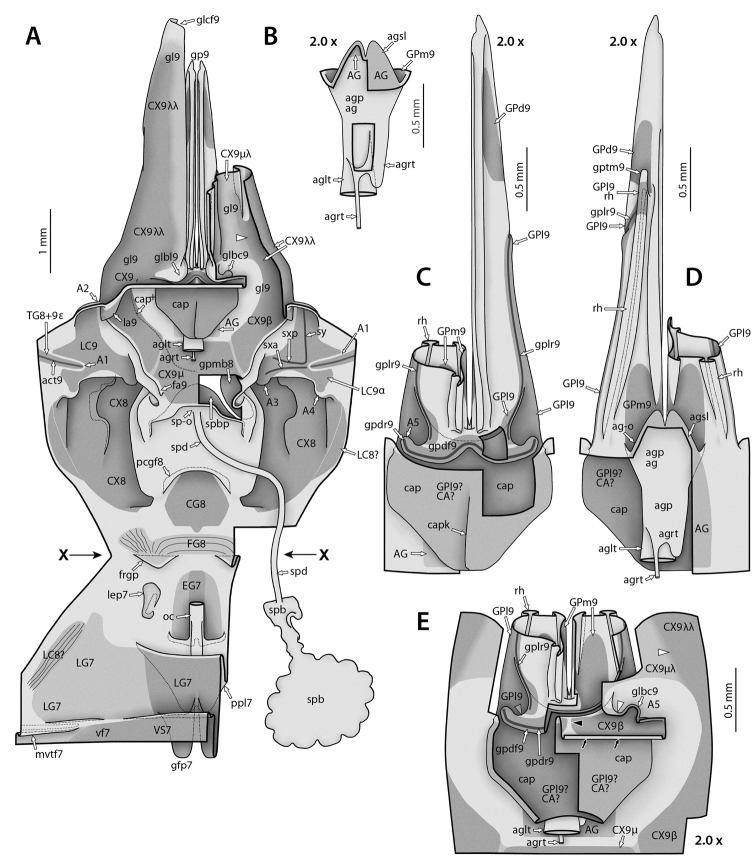
Annotated illustrations of the female *Sphodromantis* sp. postabdomen and genitalia for morphological use. **A** genital region, most parts in internal view. Posterior parts (segments VIII, IX) are bent 180° dorsally and to the anterior compared to anterior parts (of segment VII), along the axis marked by arrows X. The lower half of the illustration shows part of the dorsal wall of the ventral fold 7 (“subgenital lobe”) and genital fold area in ventral view; the upper half of the picture shows the ventral sides of segments VIII and IX in dorsal view. Right gonoplac 9 cut open **B** accessory gland with some structures surrounding its opening **C** gonapophyses 9 and structures around their base in dorsal view, left gonapophysis 9 cut near base (accessory gland not included) **D** gonapophyses 9 and structures around their base in ventral view, left gonapophysis 9 cut near base, outlet tubes of accessory gland (**aglt, agrt**) cut near base **E** base of gonapophyses 9 (right gonapophysis 9 cut open) together with central apodeme and adjacent parts of coxa 9 sclerotizations and gonoplacs 9. Thick black lines are (virtual) cutting lines. Continuous thin black lines are freely visible edges (= lines along which the cuticle bends away from the observer’s view). Dashed thin black lines are (parts of) edges hidden beneath other cuticle. Thick gray lines are internal ridges. Dashed gray line labeled **cap^+^** in (**A**) (left side only) indicating maximum size of central apodeme cap in other specimens. Membranous cuticle in very light gray, sclerotized cuticle in medium to darker gray (darker = more strongly sclerotized); cuticle shaded darker where it dives beneath other cuticle. For meaning of black arrows and black, white, and gray arrowheads see text. For abbreviations see text, glossary, or Suppl. material 2: Extended abdominal glossary.

**Figure 16.**
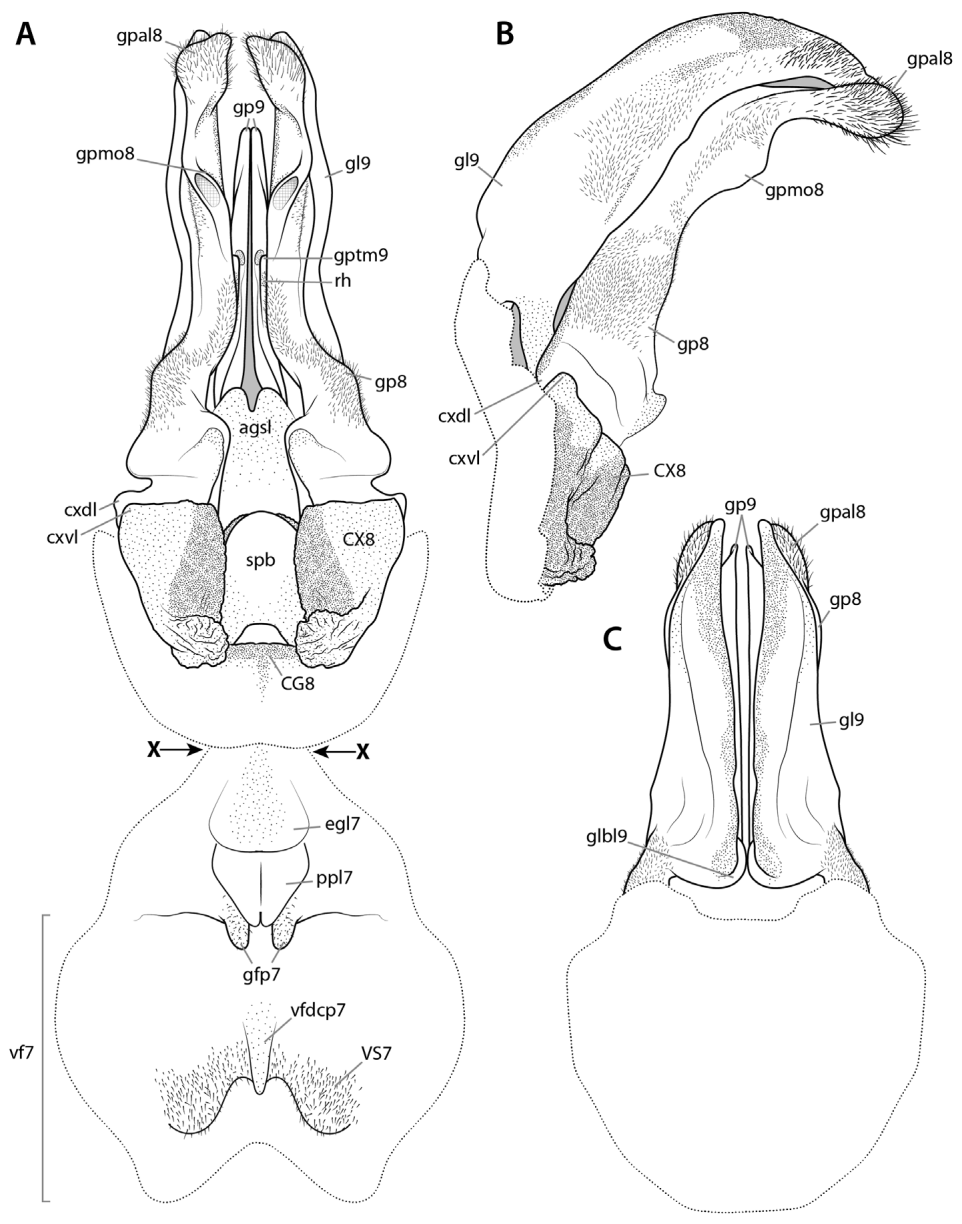
Annotated illustrations of intact female genitalia *Tenodera sinensis* for taxonomic use. **A** segments are opened up 180° along the axis marked X, the upper half of the illustration shows the ventral side of segments VIII and IX in dorsal view, the lower half of the illustration shows the dorsal wall of the ventral fold **vf7** and genital fold in ventral view **B** segments VIII and IX in lateral view **C** dorsal side of segments VIII and IX in dorsal view. Dotted lines indicate regions of membrane, connective tissues, and musculature. Abbreviations: **agsl** = accessory gland supporting lobe; **CG8** = caudogyne; **CX8** = coxa 8; **cxdl** = dorsolateral coxal lobelet; **cxvl** = ventrolateral coxal lobelet; **egl7** = epigynal lobe; **gfp7** = process of genital fold; **gl9** = gonoplac 9; **glbl9** = gonoplac basal lobe 9; **gp8** = gonapophysis 8; **gp9** = gonapophysis 9; **gpal8** = apical lobe of gonapophysis 8; **gpmo8** = medial outgrowth of gonapophysis 8; **gptm9** = medial tine of gonapophysis 9; **ppl7** = papilla lobe; **rh** = rhachis; **sbp** = spermathecal bulge; **vf7** = ventral segmental fold 7; **vfdcp7** = process of dorsal carina of ventral fold 7; **VS7** = vestibular sclerite 7.

**Figure 17.**
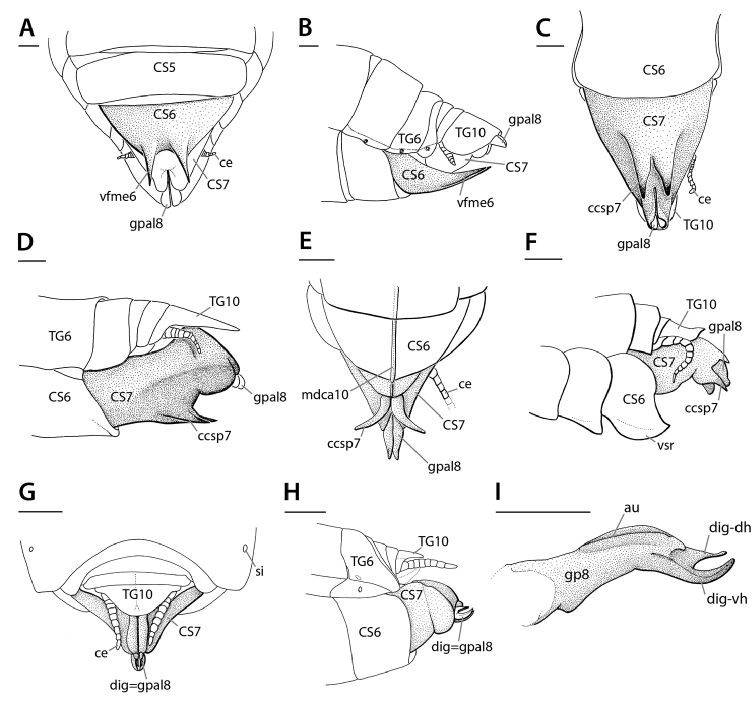
Annotated illustrations of digging structures on female genitalia. **A**–**B** Eremiaphilidae-type digging spine: *Eremiaphila* sp. terminalia **A** ventral view **B** lateral view. **C**–**D** *Rivetina*-type digging spine: *Rivetina* Berland & Chopard, 1922 terminalia **C** ventral view **D** lateral view. **E**–**F** *Chroicoptera*-type digging spine: *Chroicoptera longa* Giglio-Tos, 1915 terminalia (**E**) ventral view; *Chroicoptera saussurei* Giglio-Tos, 1915 **F** lateral view. **G**–**I** *Ligaria*-type digging spine: *Ligaria* Stål, 1877 **G** dorsal view **H** lateral view **I** lateral view (left side) of the left gonapophysis 8. Note that in the *Ligaria*-type, the digging structures are gonapophyses 8. Figures **A**–**E** and **G**–**I** reproduced and adapted from Wieland (2013); figure **F** reproduced from Wieland 2013, which was modified from Kaltenbach 1996. Abbreviations: au = aulax; ccsp7 = centrocoxosternal processes 7; ce = cercus; CS5–7 = coxosternite 5–7; dig-dh = digging device, dorsal hook; dig-vh = digging device, ventral hook; mdca10 = middorsal carina 10; vfme6 = ventromedian expansion (of ventral fold); gpal8 = apical lobe of gonapophysis 8; si = spiracle; TG6–10 = tergite 6–10. Scale bars: 1 mm.

At present, praying mantis species are most frequently delimited through observable differences in external morphological and genitalic features (e.g., Svenson 2014, Agudelo and Rafael 2014, Agudelo and Rivera 2015, Svenson et al. 2015, Rivera and Svenson 2016, Brannoch and Svenson 2016a, b, Svenson et al. 2016, Rodrigues and Cancello 2016, etc.). The reliance on genitalia to make taxic determinations necessitates a morphological investigation of both the male and female genitalia as well as the pre- and post-genital segmentation of the abdomen (see Suppl. materials 6–8: Abdominal structure terminology; Male genital structure terminology; and Female genital structure terminology; Figs 10–17). Furthermore, as enhancements to imaging technology are continuously being improved, thereby allowing taxonomists and morphologists better access to the morphological nuances of insects, we present a thorough morphological investigation of the terminal elements of the abdomen including a new terminological approach with supporting figures (Figs 10–12; 14–15). To supplement this morphological investigation, we provide annotated scientific illustrations of genitalia to demonstrate the level of morphological information generally used in taxonomic works as well as the illustrative style generally employed (Figs 13, 16–17). This information is presented with the hope that others will draw useful morphological and taxonomic information to apply to their own studies of Mantodea.

**Abdominal structures**

The abdomen of Mantodea consists, as primitively in insects, of 11 segments plus a non-segmental apical telson. The segments preceding the genitalia comprise the pregenital segments. Among these, the 1^st^ is strongly modified by forming the transition to the thorax, and the 2^nd^ and 3^rd^ segments also differ to some extent from the ‘typical’ midabdominal segments following them. The segments showing morphological modifications for genital functions are the genital segments, which in Mantodea are segments 7–9 in the female and segment 9 alone in the male (although the male genitalia are probably contributed by segment 10, see below). Segments 10 and 11 are the terminal or postgenital segments, which are even more strongly modified than the genital segments, and partly reduced. The genital and terminal segments comprise the postabdomen.

The morphological literature on the abdomen of Mantodea is quite limited. The exoskeleton and musculature of the entire abdomen, including the genitalia, were treated by Levereault (1936: exoskeleton; 1938: musculature) for *Stagmomantis* Saussure, 1869 and by LaGreca and Rainone (1949) for *Mantis* Linné, 1758. Ford (1923) also treated the musculature of *Stagmomantis*, but the documentation of the exoskeleton is poor and difficult to understand. The male genital and postgenital segments were specifically studied by Walker (1922, exoskeleton of *Stagmomantis*), Snodgrass (1937, exoskeleton and musculature of *Tenodera* Burmeister, 1838), LaGreca (1954, partial documentation of exoskeleton of genitalia of 16 Mantodea species), and Klass (1995, 1997). The latter author presents data for the basally diverging genera *Chaeteessa* and *Mantoida*, for *Metallyticus*, and for the highly derived *Sphodromantis* Stål, 1871, including the musculature for *Mantoida* and *Sphodromantis*. Specific studies of the female genital and postgenital segments were contributed by Walker (1919, exoskeleton of *Stagmomantis*), Marks and Lawson (1962, part of exoskeleton of *Stagmomantis*), and Klass (1998, exoskeleton of *Sphodromantis*). In Wieland’s (2013) otherwise fundamental work on Mantodea morphology, only few selected elements of the abdomen and genitalia are considered.

Accordingly, knowledge of the various parts of the abdomen has remained limited to a few species of higher Mantodea; the only exception is the inclusion of “basal” mantodeans with regard to the male genitalia. In addition, though their quality varies, none of the abovementioned works (with the exception of Klass 1995, 1997) provides a comprehensive description of the addressed parts of the mantodean abdomen. Most abdominal morphology is thus still unknown for the greatest part of the Order.

Regarding male and female genitalia, the limited existing morphological knowledge demonstrates that these highly complicated parts of the mantodean body are a very useful source of characters for taxonomic work. For review of the historical treatment of male and female genitalia in mantodean taxonomy see Brannoch and Svenson (2016b).

In the male and female genital segments and the terminal abdomen, the morphological interpretation regarding the homology and homonomy of many elements has long been strongly disputed, which is partly evident from the heterogeneous terminologies used by the various authors and by some lengthy and complicated discussions in relevant literature. However, most of the issues could be resolved in the coming years by comparative work at the insect level (e.g., Klass 2001, 2003, 2008; Klass and Matushkina 2012; Klass et al. 2012) – although some elements have remained problematic.

The following descriptions are predominantly based on Klass (1995, 1997, 1998) concerning the genitalia of both sexes, with a focus on *Sphodromantis*. We use a new terminology for this, which includes all recent evidence on morphological interpretations, described below. Due to the limited taxic coverage of abdominal morphology in Mantodea, conditions in some taxa explored in the future might diverge considerably from the descriptions following below. We hope that the following will provide inspiration to do such exploration in a taxonomic framework.

**Basics of a new abdominal terminology**

In recent years, K.-D. Klass and other researchers have done much comparative morphological work on the cuticular exoskeleton, musculature, and nervous system of the abdomen of various insects, with a focus on male and female genitalia (e.g., Klass 2003, 2008, Klass and Matushkina 2012 for females; Helm et al. 2011 for males). Homologies between taxa and homonomies between segments and sexes have been core issues in these works. It became evident that traditional ways of naming exoskeletal structures were insufficient and too poorly standardized for morphological terms to include information on the kind of element they address and on the homology or homonomy hypothesized. Therefore, a new terminology for elements of the cuticular exoskeleton has been gradually developed that should minimize these shortcomings.

(*1*) *Sclerotizations*: These are areas of the cuticle that are hardened to a varied extent, in contrast to fully flexible membrane. Sclerotizations are usually colored (yellowish to black), but this is not always the case. We view a set of ‘principal sclerotizations’ of the abdominal segments; this essentially comprises sclerites as putatively present in a plesiomorphic insect condition (e.g., Archaeognatha; see Klass and Matushkina 2012) plus sclerites that appeared later in evolution but are not (or not clearly) derived from members of the plesiomorphic set. Many of the widely used full names for sclerites can either end in -ite (e.g., coxite, tergite) or in -a/-um (e.g., coxa, tergum). The former terms should only be used when a sclerotized area forms a discrete and undivided sclerite, while the latter terms are also applicable when the addressed piece of sclerotization is fused with another or is subdivided. The abbreviations used herein for principal sclerotizations are composed of two uppercase letters (e.g., **TG** = tergum/-ite); subdivisions of principal sclerotizations, those separated by membrane or distinctly weakened sclerotization, are specified by a lowercase letter in the third position (e.g., **TGp** = paratergite). In case of homonomous sclerotizations occurring in several segments, the terms can have a number in the last position, which specifies the abdominal segment to which the element belongs (e.g., **TGp9**). In addition, for descriptive purposes it is often convenient to address particular areas of sclerites that are not or less distinctly separated from the remainder. For such areas one or two lowercase Greek letters are used (e.g., **TG8π** = paratergal area), which are placed behind the segmental indication. Fusions of sclerites are expressed by “+,” such as **CX9+LCp9** (an intrasegmental fusion) or **CX8+LC9** (an intersegmental fusion). If a fusion occurs widely, a new name can be given to the resulting compound sclerite; for instance the compound sclerite **CX6+LCa6+LCp6+ST6**, including most of the original ventroabdominal sclerites, is called **CS6**, the coxosternite of segment 6. Articulations between sclerites, ranging from simple areas of close contact to elaborate ball-and-socket joints, are expressed by “-”, such as **CX9-LCp9** (intrasegmental articulation) or **CX8-LC9** (an intersegmental articulation). In a shorter but less formal way, articulations can be called **A1**, **A2**, etc.

The terminology for the sclerotizations of the male genitalia was established (Klass 1995, 1997) before the above terminology was developed and thus differs from it. First, only a single uppercase letter has been used to categorize them: **L** for the left part of male genitalia and **R** for the right part. Second, a number following the letter distinguishes the principal sclerites of the phallic organs (e.g., **L4**). Third, another uppercase letter denotes isolated sclerites resulting from the subdivision of a principal sclerite (e.g., **L4B**), while a lowercase letter denotes a particular region of the main sclerite (e.g., **L4d**; no matter whether it is separated from the rest or not). As its implementation is widespread in Mantodea morphology, taxonomy, and systematics, this terminology is here retained for convenience.

(*2*) *Formative elements*: These are more or less discrete in- and evaginations of the cuticle or discrete thickenings of the cuticle (upon either membranous or sclerotized cuticle). Abbreviations are composed of 2–4 lowercase letters (e.g., **cx**, **pda**, **frgp**). The terms can have a number in the last position, which specifies the abdominal segment to which the element belongs (e.g., **cx8**). An appended ‘-o’ denotes the external / internal opening of a strongly invaginated / evaginated formative element (e.g., **sp-o** is the external opening of the female spermatheca **sp**). Usually the borders of a formative element cannot be exactly defined, as the body wall gradually bulges inward or outward in its periphery. The body wall areas of different formative elements are not necessarily mutually exclusive; two cases are particularly important: First, a less inclusive formative element can be a part of another, more inclusive formative element. For instance, the lobe **vla** of the male genitalia can bear a distinct process **pda** arising from its distal edge (Fig. 10d); **pda** is entirely part of **vla**. Second, neighboring formative elements of different orientation (inward versus outward) can have parts of their walls in common. For instance, the left part of the dorsal wall of lobe **vla** is at the same time the ventral wall of the pouch **dee** above it (Fig. 10d). Fusions of formative elements of the same orientation (external or internal) can occur, which are expressed by “+.” For instance, the usually independent processes **paa** and **pda** of the male genitalia (Fig. 10c) can be partially fused, having a common stem, and the entire complex is then called **pda+paa** while the free terminal parts are still called **paa** and **pda**. The same two-letter combinations are frequently used for formative elements and sclerotizations that are approximately co-extensive or otherwise closely associated (e.g., **gp** for the gonapophysis and **GP** for its sclerotization). But one should keep in mind that the degree of co-extensivity can strongly vary across taxa.

The terms used for formative elements of the male genitalia (Klass 1995, 1997) fit into this scheme but no segmental assignment is required. Their abbreviations are composed of 3 lowercase letters. The first two letters are either derived from the abbreviation of the element in LaGreca (1954: pp. 27, 28, e.g., lamina ventral = **lv**) or give some characteristic (e.g., **vl** = ventral lobe). The last letter is either ‘a’ or ‘e’ (e.g., **lve**, **vla**), ‘a’ standing for the German ‘auswärts’ (= directed outward: evagination), and ‘e’ for ‘einwärts’ (= directed inward: invagination).

For additional discussion regarding this terminology see Suppl. material 9: Abdominal terminology discussion.

**Pregenital abdominal segments**

The pregenital segments of Mantodea are fairly simple in structure. They bear an undivided ventral sclerotized plate without special differentiations, which is generally called sternite but preferably addressed coxosternite (**CS**). The coxosternite of the 1^st^ abdominal segment (**CS1**) is very small and hidden, i.e., the anterior-most freely visible coxosternite belongs to segment 2 (**CS2**). In addition, each segment bears a large, undivided dorsal plate, the tergite (**TG**). Its lateral parts are bent downward, usually along an angled longitudinal edge, the laterodorsal carina (**ldca**). These lateral parts are not separated by membrane from the dorsal main part, but there can be indistinct separation by weaker sclerotization. These lateral parts are best called the paratergal areas (**TGπ**) and are formally part of the tergite. On segment 1, the paratergal areas are absent. The dorsal remainder of a tergite is here called the centrotergal area (**TGκ**).

The posterior part of each coxosternite and tergite extends over the anterior portion of the successive coxosternite or tergite, and from its posterior margin a membrane is reflected to the anterior to reach the anterior margin of the overlapped successive sclerite. Both on the dorsal and ventral side, the posterior part of a segment thus forms a transverse fold that covers part of the following segment: the dorsal (**df**) and ventral (segmental) folds (**vf**). The reflected membrane is often called the ‘intersegmental membrane,’ but this should be avoided, because with regard to primary segmentation the membrane entirely belongs to the segment in front. The membrane is better called intercoxosternal membrane (ventrally) or intertergal membrane (dorsally), or conjunctival membrane (both sides).

Spiracles (**si**) and associated spiracle sclerites (**SI**) are present on abdominal segments 1–8. Each includes a posterior spiracle sclerite (**SIk**; instead of **K**) and an anterior spiracle sclerite (**SIm**; instead of **M**), both being considered subsets of the spiracle sclerotization **SI**.

Wieland (2013: chars. 145–148) described expansions of abdominal segments, which basically occur in four different positions: dorsomedian, dorsolateral, ventromedian, and ventrolateral. The dorsomedian (**dfme**) and ventromedian (**vfme**) expansions result from a spatially limited lengthening of the dorsal or ventral segmental folds (**df, vf**) and are reasonably denoted as parts of these. The dorsolateral segmental expansions (**dlse**) apparently result from a strong elevation of part of the laterodorsal carina (**ldca**). The ventrolateral segmental expansions (**vlse**) are elevated from the lateral parts of the coxosternite. Other types of segmental expansions may exist that do not fulfill the positional criteria of the 4 types treated above; such elements should receive different names. A special kind of a ventromedian expansion of the ventral fold (**vf6**) can be seen in the paired spine-like projections on coxosternite **CS6** of female Eremiaphilidae (**vfme6**) (Fig. 17a–b), which assist oviposition by digging and originate from a generally elongated ventral fold **vf6** (Wieland 2013: chars. 139, 141; ‘dig’ in figs 365, 367). Although the morphological origin of digging structures in female Mantodea is often identical (see Fig. 17), homology of digging structures in different genera is not likely (Wieland 2013: 170 ff.). Wieland (2013) therefore decided to informally name these structures in regard to the genera or larger taxonomic groups displaying them (e.g., Eremiaphilidae-type, *Chroicoptera*-type, *Ligaria*-type, *Rivetina*-type). These informal names refer to a set of adaptations for digging on several abdominal sclerites and/or the ovipositor, which is characteristic for the respective group. As new types of digging devices have been discovered (F. Wieland, unpubl. data), these informal names will be maintained to describe systems of digging structures in Mantodea.

**Male genital region**

Tergite 9 (**TG9**) resembles the tergites of the preceding segments. Coxosternite 9 (**CS9**), however, shows many peculiarities (see Klass 1995, 1997: ‘S9’ in figs 4, 22, 30, 40; Fig. 12): First, the anterior margin of coxosternite 9 is usually expanded anteriorly, and the median part of the expansion is additionally dragged out in a pair of mediocoxosternal apodemes (**mcsa9**). Second, the posterior part of coxosternite 9 and the anteriorly reflected cuticle following it, which together form the ventral fold 9 (**vf9**), are strongly expanded posteriorly. This long lobe **vf9** covers the entire phallic organs ventrally and is thus functionally a ‘subgenital lobe’; accordingly, coxosternite **CS9** has the function of a ‘subgenital plate.’ As a third peculiarity, the posterior edge of fold **vf9** bears a pair of styli (**sl9**, having their own sclerite **SL9**), which is the only pair of styli retained in adult Mantodea. In some Mantodea styli are absent (Wieland 2013: char. 142). As styli easily break off, absence should only be stated after examination of the continuity of the cuticle; a pair of small holes upon the hind edge of fold **vf9** suggest broken off styli.

**CS9** and **vf9** can show asymmetry in various parts: in the anterior apodemes **mcsa9**, in the hind edge of **vf9** (potentially including an asymmetric positioning of the styli), in the dorsocoxal sclerotization **CS9δ** (as in Fig. 12), or in overall shape (Klass 1997; Wieland 2013: char. 137). In a description, it should be clearly stated which parts are asymmetrical and in which ways, and the asymmetries must be correlated with the orientation of the asymmetry of the male genitalia (see below).

The male genitalia (= phallic organs, Figs 10–11, 13) follow where the dorsal wall of **vf9** bends back to the posterior, and they rest upon the dorsal wall of **vf9**. The segmental assignment of the phallic organs is not clarified: they may belong to segment 9, but more likely they are elements of segment 10 –either part of the 10^th^-segmental limbs or formations independent of limbs.

The phallic organs are strongly asymmetrical. Homonomies between the elements of the left and right parts are unresolved –and may not exist. The normal (dextral) orientation of the phallic asymmetry is as in Figs 10–11, 13 (see also Klass 1997: figs 3, 24, 31, 38). However, a few mantodean genera (e.g., *Ciulfina* Giglio-Tos, 1915, *Haania* Saussure, 1871) include species with a side-reversed asymmetry, i.e., the phallic organs are mirror-imaged compared to those of other mantodeans (sinistral orientation), or species where both dextral and sinistral males occur (e.g., Balderson 1978, Anisyutkin and Gorochov 2004, Holwell et al. 2007, 2015). In cases of a sinistral orientation, the terms for left and right structures of the phallic organs must also be applied in a side-reversed way. In addition, in those species, sinistrality in **CS9** and **vf9** must also be assessed.

The phallic organs of Dictyoptera were often divided in a left phallomere, a right phallomere, and a ventral phallomere. This subdivision, however, is conflicting with regard to the interpretation of the ‘ventral phallomere’ (ventral lobe **vla** in Fig. 10) as a left, right, or median element (see Klass 1997: pp. 17–19 for a more complete discussion). The evidence contributing arguments to this topic comes from various blattodeans, but as the homology of lobe **vla** between Blattodea and Mantodea is clear, the arguments can be applied to Mantodea as well. Studies of the nymphal development in blattodeans have variously found lobe **vla** to be a median element (Snodgrass 1937) or a right element (Qadri 1940), the latter hypothesis being based on inclusion of younger nymphs than in the former. However, the innervation of lobe **vla** through nerves of the left side only (Pipa 1988) strongly suggests this part to belong to the left side of the body. The same results from two aberrant male cockroaches with bilaterally symmetrical phallic organs, one having a left phallomere plus a ventral phallomere on each side (in a mirror-imaged fashion), the other one having a typical right phallomere on each side (in a mirror-imaged fashion) but no ventral phallomere (K.-D. Klass unpubl. data). This led Klass (1995, 1997) to divide the phallic organ into a right part, the right phallomere (the traditional right phallomere only) and a left part, the left (phallic) complex (including the formerly distinguished left and ventral phallomeres). However, the nymphal ontogeny of the phallic organs should be revisited, best under inclusion of the innervation.

Both the right phallomere and the left phallic complex consist of a variety of processes, pouches, and apodemes, and each bears several sclerites. Internally they are equipped with a rich musculature. In Mantodea the morphology of phallic organs is fairly uniform, especially compared to the much greater structural diversity in Blattodea. Yet, there are some exceptions to this.

Left phallic complex (Fig. 10, 13c–d): both the dorsal and ventral walls are essentially level. The ventral wall is more strongly expanded to the anterior and thus longer (Fig. 10a). Along the left edge of the left complex, the dorsal and ventral walls bend directly into each other. In the right and right-posterior parts, however, there are several foldings of the body wall superimposing each other.

Ventral to the pouch **pne**, the cuticle is folded outward to form a more or less continuous edge that is drawn out into a maximum of known processes, which from anterior to posterior are (see Fig. 10b–c and Snodgrass 1937: fig. 10): **afa**, **apa**, **loa**, and **paa**. LaGreca (1954: fig. 3) considers process **apa** as part of process **afa**, but the processes are distinctly separate in many Mantodea and thus named separately herein (**apa** refers to ‘apophysis, posterior [process]’). The four processes vary considerably in their shape and in the extent and position of sclerotization upon them (see below). As some of these processes can be absent or fused, the homology of the individual remaining processes can be difficult to resolve. If a fusion of certain processes is hypothesized, the product can be called, for instance, **afa+apa**. In *Chaeteessa* and *Mantoida* the area bearing the processes is highly distinct (Klass 1997: figs 34, 45). For practical reasons we further introduce here the term **pba** (referring to ‘process bearing’) for the four processes together plus the edge from which they arise.

Beneath this process-bearing edge **pba**, the cuticle is folded inward again to form another deep pouch **lve** (Fig. 10c–d). Ventral to this pouch the cuticle is again folded outward to form the lobe **vla**. The posterior edge of lobe **vla** is drawn out into another process **pda**, which is a specific part of lobe **vla**. The ejaculatory duct (**dej**) opens into the dorsal wall of lobe **vla** (Fig. 10d). On the mesal and anterior flanks of the opening of the ejaculatory duct there are often membranous lobes of varied size and shape, which are subsumed as lobes **goa**.

The left phallic complex bears three principal sclerites: **L1**, **L2**, and **L4** (sclerite **L3** only occurs in Blattodea; Klass 1997).

The principal sclerite **L4** covers much of the dorsal and ventral walls of the left complex. The ventral portion of **L4** is usually plate-like (Klass 1997: figs 6, 20, 28), but in *Mantoida* **L4** is horseshoe-shaped and frames the ventral wall (Klass 1997: fig. 41). While in *Chaeteessa* and *Mantoida* the dorsal and ventral parts of **L4** are fully continuous around the left edge of the left complex, in all other examined taxa (apparently including all shown in LaGreca 1954) **L4** is divided into a dorsal **L4B** sclerite and a ventral **L4A** sclerite.

The principal sclerite **L1** extends over parts of the walls of pouch **pne** and of the edge **pba** between pouches **pne** and **lve**; in the latter area it is limited to the anterior part bearing the processes **afa**, **apa**, and **loa** (Fig. 10b–c), onto which **L1** extends to a varied extent. While sclerite **L1** is undivided in *Chaeteessa* and *Mantoida* (Klass 1997: figs 4, 45), at least some of the remaining Mantodea show a complete division into two sclerites **L1A**, located in pouch **pne**, and **L1B**, located upon edge **pba** and its processes.

The principal sclerite **L2** takes most of the dorsal wall of pouch **lve** (Fig. 10b–c); smaller parts of **L2** can bend in the marginal ventral wall of pouch **lve**. Posteriorly, sclerite **L2** additionally extends into the ventral wall of the process **paa** (Fig. 10c); it also bends into the dorsal wall of **paa**, but part of this is usually left membranous (Fig. 10b). The two areas of sclerite **L2** can be distinguished as **L2l** (in pouch **lve**) and **L2p** (on process **paa**; Fig. 10b–c). The right margin of part **L2l** of the sclerite is in contact with the ventral left margin of sclerite **L1** or **L1B**: articulation **L1b-L2l** or **L1B-L2l** depending on the condition of **L1**.

As far as is known, the sclerites **L1**, **L2**, and **L4** are separate from each other. The only known example of a fusion is the one between **L2** and **L4** at the bases of the processes **paa** and **pda** in *Mantoida* (Klass 1997: figs 41, 46). This is based on a likewise peculiar close association of the bases of these processes in *Mantoida*, where they have an approximately common base (compound process **pda+paa**).

When examining the phallic organs, one should be aware that additional, smaller sclerites may occur. The only known example is sclerite **L5** of *Metallyticus*, which is located in the otherwise membranous dorsal wall of the ventral lobe **vla** (Klass 1997: fig. 24).

The more or less distinct edge **pba** bearing the processes **afa**, **apa**, **loa**, and **paa** and the associated or neighboring parts of sclerites **L1** and **L2** surely form the most complex and variable part of the left phallic complex of Mantodea. Interesting characters concern the presence and shape of the processes and the extension, the kind of subdivision, and the kind of mutual contact of the sclerites **L1** and **L2** in this area. An exploitation of the many characters offered by this area requires a very detailed examination.

The right phallomere (Fig. 11, 13e) is conveniently divided into an anterior part and a posterior part, which consists of a large lobe projecting to the posterior, the **fda** lobe. Its shape varies a bit, including the presence in at least *Tenodera* of a mesobasal accessory lobe (a part of lobe **fda**; not distinct in the taxa studied in Klass 1997). The right-proximal ventral wall of lobe **fda** bears two approximately tooth- or ridge-like projections: process **pva** further anteromesally, and process **pia** further posterolaterally (Fig. 11c–d; 13a, e). The processes **pia** and **pva** can be fully separated (as in *Chaeteessa*, Fig. 11d) or have a very short common base (as in *Sphodromantis*, Fig. 11b). Their shape varies strongly; process **pva** can either be limited to the anterior ventral wall of lobe **fda** (Fig. 11c) or reach far to the posterior (Fig. 11d). In some Mantodea with a distinct process **afa** on the left complex (Fig. 10a–b), the **afa** approaches the processes **pia** and **pva** (gray dashed lines, Fig. 11a–c) and seems to interact with them possibly as a clasper.

Sclerite **R1** is a very complex principal sclerite; it takes most or all of the dorsal wall of lobe **fda**, and along the lateral edge of lobe **fda** it also bends into its ventral wall to expand over the processes **pia** and **pva** and (usually) the area between them (Fig. 11). **R1** was divided into four areas (Klass 1997: fig. 330a–e): **R1d** (‘dorsal’) includes most of the dorsal part, except the area where **R1** bends ventrally to reach process **pia**; **R1v** (‘ventral’) is the sclerotization of process **pia**; **R1t** (‘tooth’) is the sclerotization of process **pva**; **R1c** (‘central’) is the remaining sclerotization in between the three other areas, bearing articulation **A3**.

The anterior part of the right phallomere consists of the anteriorly expanded ventral wall of the phallomere, which in its larger anterior part is sclerotized by sclerite **R3** (Fig. 11a, c–d). The deepened **R3**-bearing anteroventral part of the right phallomere has been considered an apodeme, while its condition is basically not different from that in the anteriorly deepened anteroventral part of the left complex sclerotized by **L4A** (Klass 1997: fig. 28). However, sclerite **R3** always bends a bit around the bottom of the deepening into the dorsal wall of ventral fold **vf9** (Fig. 11d, where only the sclerotized part of the **vf9** wall is retained). The sclerotized groove thereby formed along the anterior margin of sclerite **R3** is called **age** (referring to ‘anterior groove’).

**Female genital region**

Tergite 7 (**TG7**) and spiracles 7 and 8 agree with their counterparts of the preceding segments. Tergite 8 (**TG8**) and Tergite 9 (**TG9**) (Fig. 14c) are both short (ca. ½ the length of **TG7**), and both have a pair of large paratergal areas 8 (**TG8π**) and paratergal areas 9 (**TG9π**), which are indistinctly separated from the dorsal main parts, the centrotergal area 8 (**TG8κ**) and the centrotergal area 9 (**TG9κ**), respectively, by weaker sclerotization. In this way, they could thus alternatively be considered as separate sclerites: paratergites 8 and 9 are detached from centrotergites 8 and 9. In addition, both **TG8π** and **TG9π** are extended ventromesally: **TG8π** at the posterior margin of segment 8 and **TG9π** at the anterior margin of segment 9. At the segmental border the two extensions are fused to form the paratergal extension (**TG8+9ε**).

Coxosternite 7 (**CS7**) and ventral fold 7 (**vf7**) show many peculiarities (Figs 14c, 16a) compared to the preceding coxosternites and ventral folds: First, the anterior margin of **CS7** is usually expanded a bit anteriorly, and the median part of the expansion is additionally dragged out in a pair of short mediocoxosternal apodemes 7 (**mcsa7**; partly seen through a window in Fig. 14c). Second, the posterior part of **CS7** and the anteriorly reflected cuticle following it, which together form the ventral fold of segment 7 (**vf7**), are strongly expanded posteriorly. This long fold or lobe **vf7** covers the ventral sides of segments 8 and 9 and most of the ovipositor ventrally and is thus functionally a ‘subgenital lobe’; accordingly, **CS7** has the function of a ‘subgenital plate.’ The posterior (apical) part of ventral fold **vf7** is bilobate, whereby a pair of ventroterminal lobes 7 is formed, which are part of **vf7**. The posterolateral parts of the ventral fold **vf7** are bent upwards, so that in a natural posture the left and right ventroterminal lobes enclose a vertical slit. The **CS7** sclerotization of the ventroterminal lobes is continuous with the remainder of **CS7**. The apices of the ventroterminal lobes can be shaped to form a pair of pointed elongate processes, which assist oviposition by digging (Wieland 2013: ‘dig’ in figs 376, 377). Coxosternite **CS7** can furthermore bear a pair of ventral spines in its central part, which represent another digging device for oviposition (Wieland 2013: char. 140; ‘dig’ in figs 369, 370); these are called herein the **centrocoxosternal processes 7** (**ccsp7**) (see Fig. 17).

The anteriorly reflected cuticle lying above the posterior part of coxosternite **CS7** is highly differentiated (Figs 14c, 15a) and comprises two regions: The dorsal wall of the ventral fold **vf7** (around sclerite **VS7**) and the genital fold area (around sclerite **LG7**). Regarding the overall orientation of the animal, the latter is located anterior to the former. However, in a strict morphological sense, the genital fold area is located *posterior* to the dorsal wall of the ventral fold **vf7**, since the cuticle of the two areas is reflected anteriorly. The space enclosed above the ventral fold **vf7** is the vestibulum (**vst**); the smaller space enclosed above the genital fold area is the genital chamber; the two spaces together can be called the genital pouch.

Most of the dorsal wall of ventral fold **vf7** is sclerotized by the large vestibular sclerite (**VS7**), which is not in contact with the coxosternite **CS7**. **VS7** is homonomous with the dorsal sclerotization of ventral fold **vf9** in the male, called **CSd9** if separated, and could alternatively be termed **CSd7**. While most of sclerite **VS7** is very weak, its anteromedian part is heavier. At the midline the heavier sclerotization reaches further posteriorly along a low ridge-like elevation, the dorsal carina of ventral fold 7. From the upward-bent edge of the anterior part of each ventroterminal lobe, a fold descends to the level of the vestibular sclerite: the descending fold of the ventroterminal lobe. The dorsal walls of the left and right ventroterminal lobes are transversely connected by the transverse fold of the ventroterminal lobes. Beneath this fold the cuticle extends into a large cavity, which presumably is glandular in nature and is here called the gland of ventral fold 7 (**vfgl7**, seen through window cut into right dorsal wall of ventral fold **vf7** in Fig. 14c). At its frontal end, the dorsal wall of lobe **vf7** bears a pair of very shallow inward and forward directed folds, the marginovestibular transverse folds 7 (Figs 14c, 15a). In front of these folds the dorsal and ventral walls of lobe **vf7** are fixed upon each other by vertical columellae (likely cuticular, as they are KOH-resistent) that cross the narrow body cavity.

The genital fold area bears the languette sclerite **LG7** and three lobe-like elements around the midline, **gfp7**, **ppl7**, and **egl7**, which belong to the posterior marginal part of segment 7. The genital fold (**gf7**) is a very short transverse fold with a very shallow, slit-like cavity beneath it. The fold **gf7** is additionally expanded into a pair of paramedian processes of the genital fold (**gfp7**), which are part of the genital fold and render it strongly bilobate. Sclerite **LG7**, which in *Sphodromantis* is a transverse plate while in *Mantis* it is medially divided, ascends onto the processes **gfp7**, which are sclerotized by it all around. From the area in front of the margin of sclerite **LG7** arises a membranous papilla lobe and in front of this, the epigynal lobe. The cuticulized common oviduct opens into the space between the papilla lobe and the epigynal lobe, and in the dorsal wall of the papilla lobe the opening is extended in a groove. Internally the common oviduct forks into a pair of lateral oviducts, into which the cuticle can extend for some distance or not (only cuticulized parts are retained after KOH maceration). The epigynal lobe bears a weak sclerite in its dorsal wall, the epigyne (**EG7**). The papilla lobe and the epigynal lobe together form the genital papilla, which thus bears the female genital opening in its center (Fig. 14c). Sclerite **EG7** is flanked by a pair of small membranous pouches here called the lateroepigynal pouches (Fig. 15a).

While the aforementioned elements pertain to segment 7, the lateral ends of sclerite **LG7** are connected with a pair of weak, finely folded sclerotizations that likely belong to segment 8, representing (part of?) the laterocoxae 8 (**LC8**). However, this interpretation remains to be tested based on the muscular connections, and there is another candidate sclerite to represent **LC8** (see below).

In front of sclerite **EG7** and pouches **lep7** the body wall is reflected to the posterior (along the transverse line indicated by X→ ←X in Figs 14c, 15a); this is the anterior bottom of the genital chamber **gc** and the ventral wall of abdominal segment 8 follows. The anteriormost part of segment 8 bears two median sclerites: the weak, finely folded frontogyne (**FG8**) and the heavier caudogyne (**CG8**). Sclerite **FG8** is probably medially divided in *Mantis* (hence a pair of sclerites: LaGreca & Rainone: i in fig. 2). The bottom edge of the genital chamber (X→ ←X) forms a pair of small pouches that are partly sclerotized by sclerite **FG8**: the frontogynal pouches (Fig. 15a). At the posterior margin of the caudogyne the body wall forms a posteriorly and outwardly directed fold, the postcaudogynal fold.

The areas posterolateral to the caudogyne **CG8** bear a pair of large, heavy sclerites, which are often sculptured and represent the limb-base sclerotizations of segment 8: the coxae 8 (**CX8**, Figs 14a, c, 15a, 16a, b). In the posterolateral part of sclerite **CX8**, two superimposing posterolaterally directed projections are present: the dorsolateral coxal lobelet (**cxdl**) and the ventrolateral coxal lobelet (**cxvl**) (Figs 14c, 16a–b). The posteromesal part of sclerite **CX8** bears a posteromesally directed projection: the mesal coxal lobelet (**cxml**). These lobelets could be parts of the original limb-base projections, the coxal lobes (**cx8**), but could as well be secondary elements of a heavy sculpturing of the coxae **CX8**. At the lateral flank of each sclerite **CX8** a small semilunular sclerite is located that is finely and incompletely detached from **CX8**, which is the second candidate to represent the laterocoxa 8 (**LC8**, Figs 14c, 15a; see above).

At the posterior margin of each sclerite **CX8** a long process originates, the gonapophysis 8 (**gp8**), the surface of which is mostly sclerotized by the gonapophyseal sclerotization 8 (**GP8**). The gonapophysis 8 shows a fairly complex structuring, and some of its formative elements have been named or are named herein (Figs 14a, c): a rounded bulge at the mesal base is called the mesal bulge of gonapophysis 8; the lobe-like apex is called the apical lobe of gonapophysis 8 (Brannoch and Svenson 2016b: AL in figs 3, 5); a membranous ventromesal lobe at ca. 2/3 of the length is the medial outgrowth of gonapophysis 8 (Brannoch and Svenson 2016b: MO in figs 3, 5); a longitudinal groove in the proximal third of the dorsal wall is the aulax (**al**; a part of the olistheter, see below), which is completely sclerotized (Fig. 14a). Sclerite **GP8** is undivided but shows a complex distribution over the walls of gonapophysis **gp8**, leaving several membranous patches, and its strength varies strongly in the different parts. The proximal part of sclerite **GP8** forms two branches: the ventral branch (Fig. 14c) forms a narrow bridge joining sclerite **CX8** whereas the dorsal branch (Fig. 14a) bends mesally, sclerotizes part of the mesal bulge, and forms the articulation **A3** with sclerite **LC9** (see below).

The last element of segment 8 is the spermatheca (Fig. 15a), which has its external opening (Fig. 14c) upon an elevated area between the coxae **CX8**. The internal part of the spermatheca consists of a slender spermathecal duct and a spermathecal bulb. The elevated area and its limiting folds are here comprised as the spermathecal bulge. The area anterior to the spermathecal opening (including fold **spba**) and the area posterior to it (including fold **spbp**) can be sclerotized by the unpaired spermathecal sclerotization (**SP**). We distinguish here the anterior sclerite **SPa** (on fold **spba**) and the posterior **SPp** (on fold **spbp**). A sclerite taking the entire spermathecal bulge would be **SP** with the areas **SPα** (anteriorly) and **SPπ** (posteriorly). The morphological interpretation of the **SP** sclerotizations is unresolved; the posterior **SPp** part could be contributed by the true sternum 9, but as for the putative sternal elements of segment 8, this term should be used with caution.

The ventrolateral areas of segment 9 bear a pair of heavy sclerites, the laterocoxae **9** (**LC9**, Figs 14c, 15a; also called gonangula). Each **LC9** sclerite can form up to four articulations: **A1** with the paratergal extension **TG8+9ε**, which is inserted into a deep U-shaped notch of sclerite **LC9**; **A2** with the lateral margin of the coxal sclerite **CX9** (see below); **A3** with the dorsal proximal arm of the gonapophyseal sclerite **GP8** (see above); and **A4** with coxa **CX8**, in the area hidden by the projections **cxvl** and **cxdl**.

The coxae 9 (**CX9**), originally a pair of sclerites, form an almost ring-shaped sclerite in Dictyoptera; the anterior half is visible in Fig. 14c, the posterior half in Fig. 15a, e. The anteromedian parts of the left and right coxae **CX9** are medially fused and are here called the mediocoxal area 9 **CX9μ**. Its coxal origin is shown by the attachment of muscles from the centrotergal area and muscles to the gonapophyses 9. From the lateral and dorsal parts of the **CX9** ring, the **CX9** sclerotization ascends onto a pair of large, posteriorly directed blade-like processes, the gonoplacs, which represent the projecting limb bases of segment 9 and can thus also be called coxal lobes 9. The gonoplacs have a slightly convex dorsolateral surface almost entirely sclerotized by **CX9** and a slightly concave, although longitudinally folded, ventromesal surface that is partly sclerotized by another part of **CX9**. For descriptive purposes, the different parts of coxa **CX9** can be addressed as the basicoxal area 9 (**CX9β**; the parts of the ring sclerite surrounding the bases of the gonoplacs, under exclusion of the anterior **CX9μ** area), the mesolobocoxal area 9 (**CX9μλ**, on the mesal surface of the gonoplac) and the laterolobocoxal area 9 (**CX9λλ**, on the lateral and dorsal surface of the gonoplac). The sclerotizations **CX9μλ** and **CX9λλ** are only connected in the regions shown by white arrowheads in Fig. 15a, e. The wide apex of each gonoplac bears a notch, the apical cleft of the gonoplac (Brannoch and Svenson 2016b: AC in figs 3, 5). The bases of the gonoplacs are fully separated from each other (Figs 15a, e), and each gonoplac forms a membranous mesal lobe at its dorsal base, the gonoplac basal lobe. Beneath this lobe there is a sclerotized cavity (part of **CX9β** sclerotization), the gonoplac basal cavity, part of which forms the socket of a ball-and-socket articulation (**A5**, Fig. 15e; see below for opposite part of **A5**).

The area enclosed by the coxae **CX9** and the gonoplac bases bears some further structures, which in anteroventral to posterodorsal succession are the following: a transverse fold whose median part is posteriorly expanded to form a bifid lobe; this is here called the accessory gland supporting lobe (**agsl**, Figs 14c, 15b, d, 16a). The ventral wall of lobe **agsl** bears a weak sclerite, the accessory gland sclerite (**AG**, Fig. 14c, 15b). The cavity above the lobe **agsl** is the base of the accessory gland(s) of segment 9 (**ag** = colleterial gland(s), Fig. 15b, d); the opening of the cavity is the accessory gland opening. The gland consists of a fairly wide chamber close to the entrance, the accessory gland pouch, which further internally forks into the asymmetrical left (wide) and right (narrow) outlet tubes of the accessory glands (left **aglt** and right **agrt**). The right **agrt** appears to originate ventrally from the left **aglt**, and is strongly narrowed internally to a wider proximal part. We here adopt the identification of left and right tubes from Kenchington and Flower (1969a: fig. 1); however, the reverse might be true as the right tube has a base further to the left than the left tube (see ventral view in Fig. 15b). Immediately dorsal to the accessory gland opening lie the bases of the paired gonapophyses 9 (**gp9**, Fig. 15c, d, e), which are homonomous with the gonapophyses 8 **gp8**. The bodies of the left and right **gp9** are fused at their very bases to form a short common stem (Fig. 15c). The **gp9** bear a variety of substructures: a ventral external carina with an Ω-like cross section, the rhachis (Fig. 15d), which fits into the aulax groove (Fig. 14a) of **gp8** to form a sliding interlock (a tongue-and-groove system), the olistheter. The distal end of the rhachis forms a finger-like process, the medial tine of gonapophysis 9 gptm9 (Brannoch and Svenson 2016b: MT in figs 3, 5; Figs 15d, 16a). Dorsally on their common stem, the gonapophyses 9 bear an anterodorsally directed transverse fold, the dorsobasal transverse fold of gonapophyses 9 (**gpdf9**, Fig. 15c, e). The fold is very low in its median part, but the lateral parts are high and each form a condyle that fits into the socket of the gonoplac basal cavity (articulation **A5**). The cuticle extending to the anterior from fold **gpdf9** enters a deep, flat cavity, the central apodeme (**cap**), which is sclerotized entirely. In addition the dorsal wall of the **cap** forms a dorsally (internally) directed median keel, the central apodeme keel. The size of **cap** varies significantly among *Sphodromantis* specimens (maximum size found indicated by gray dashed line ‘**cap^+^**’ in Fig. 15a); this may be due to continuing internal growth in adults.

Gonapophysis **gp9** bears three separate sclerites, which together constitute the gonapophyseal sclerotization 9 (**GP9**): the mesal gonapophyseal sclerite 9 (**GPm9**) (Fig. 15b, d); the distal gonapophyseal sclerite 9 (**GPd9**) (Fig. 15c, d); the lateral gonapophyseal sclerite 9 (**GPl9**) (Fig. 15c, d). At the dorsal base of the gonapophyses **gp9** (upon their common stem; Fig. 15c, e) the left and right sclerites **GPl9** are also fused, and they are together expanded over nearly the entire fold **gpdf9** and the entire apodeme **cap** (in the dorsal wall of fold **gpdf9** the sclerotization is notched: gray arrowhead in Fig. 15e). While the sclerotization of fold **gpdf9** plausibly belongs to the gonapophyseal sclerotization **GP9** (i.e., is a genuine part of sclerite **GPl9**), this is less likely for the sclerotization of the apodeme **cap**. The latter might alternatively be a formerly independent central apodeme sclerotization secondarily fused with a **GP9** sclerite (i.e., with **GPl9**). This is suggested by conditions in Odonata (Klass 2008: sclerite **CA** and apodeme **ca** in fig. 7), but clarifying the issue needs more detailed data on the musculature of Mantodea than presently available.

**Terminal abdominal segments**

**Figure 18.**
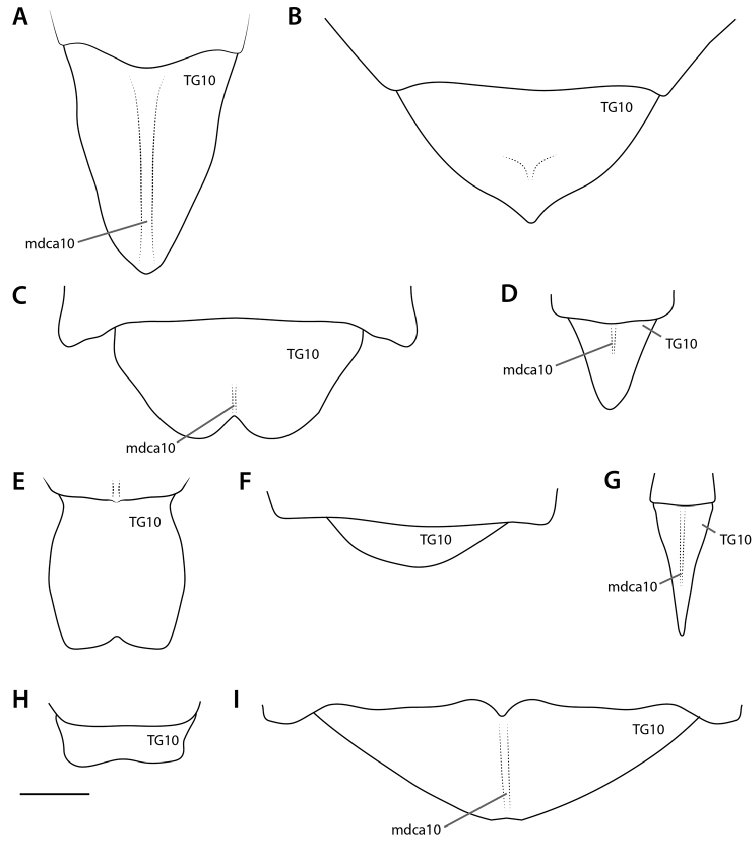
Morphological variation of supraanal plates. **A** *Brunneria* Saussure, 1869 ♀ **B** *Coptopteryx* Saussure, 1869 ♀ **C** *Acanthops* sp. ♀ **D** *Callimantis* Stål, 1877 ♂ **E** *Fulcinia* Stål, 1877 ♀ **F** *Deroplatys* Westwood, 1839 ♂ **G** *Kongobatha diademata* Heard, 1920 ♂ juvenile **H** *Metallyticus splendidus* ♀ (reproduced and adapted from Wieland (2013)) **I** *Deroplatys* sp. ♀. Abbreviations: **mdca10** = middorsal carina 10; **TG10** = tergite 10 (= supraanal plate). Scale bar: 1 mm.

Tergite 10 (**TG10**, often referred to as the supraanal plate) is a transverse plate resembling the preceding tergites, but its median part is usually more or less strongly expanded to the posterior. This part of tergite **TG10** shows much variation in shape, proportions, and the presence of a longitudinal middorsal carina (**mdca10**) (Fig. 18).

The paraprocts **PP** are a pair of ventral sclerites located posterior to the male or female genitalia (Fig. 14). Their posterior parts are placed on a pair of posteromesally projecting lobes, the subanal lobes (**sbl**, formative elements). Paraprocts and subanal lobes are very difficult to interpret in that they either belong to segment 10 or 11. The paraprocts could be composed of parts of both segments.

**Figure 19.**
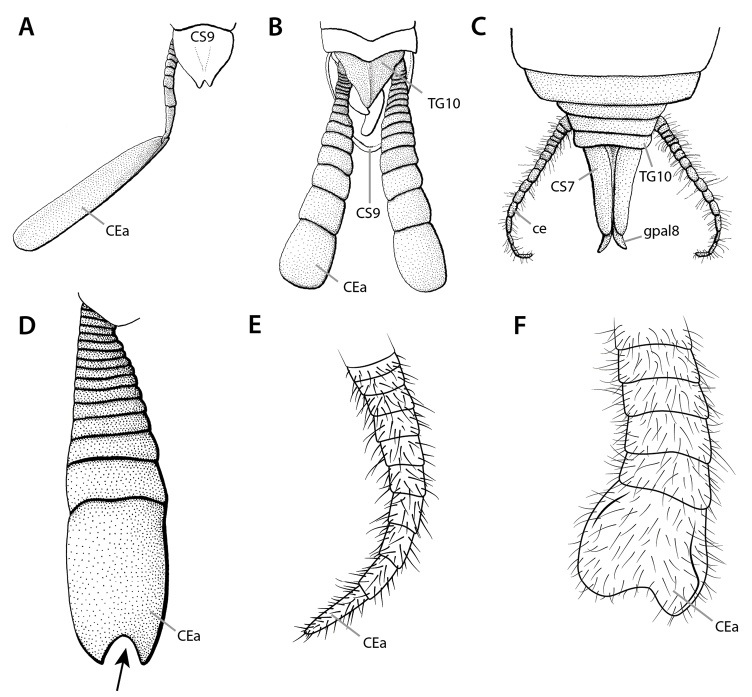
Morphological variation of cerci. **A** *Amorphoscelis* sp. ♂ in ventral view **B** *Heterochaeta bernadii* ♂ in dorsal view **C** *Metallyticus splendidus* ♀ in dorsal view **D** *Toxodera maculata* ♀ left cercus in dorsal view **E** *Ciulfina* sp. ♂ right cercus in lateral view **F** *Acanthops* sp. ♀ left cercus in dorsal view. Arrow indicates apical notch in distal cercomere. **A**–**D** reproduced and adapted from Wieland (2013). Abbreviations: **ce** = cercus; **CEa** = distal cercomere; **CS7** = coxosternite 7 (= female subgenital plate); **CS9** = coxosternite 9 (= male subgenital plate); **gpal8** = apical lobe of gonapophysis 8; **TG10** = tergite 10 (= supraanal plate). Not to scale.

The cerci are the limbs of segment 11, the exoskeleton of which consists of a series of cylindrical sclerites (all together: **CE**) separated by very narrow, more or less distinct annuli of membrane. The sclerotized sections are called cercomeres. When counting cercomeres, particular attention should be paid to the base of the cercus, where the division into sclerite cylinders is often indistinct (Wieland 2013: chars. 151, 152; proximal cercomeres are either fused or are, perhaps more likely, only incompletely separated during their ontogeny). A count not considering this is quite meaningless. Due to the great variation of cercomere number in Mantodea (Wieland 2013), it is impossible to homologize individual cercomeres among different taxa (or even among specimens) and to create a consistent terminology for cercomeres. We suggest the terms **CEa** for the apical cercomere and **CEb** for the basal cercomere (Figs 14b, 19), but note that even these may only partly be homologous among taxa. There is variation in the cross section of the cerci (flattened versus near-circular) and in the shape and length of the apical cercomere (Fig. 19) (see Wieland 2013: chars. 149, 150).

The mediotergite 11 (**TGm11**, the ‘epiproct’) is a small, weak sclerite placed behind tergite 10 at the midline, being more or less strongly overfolded by the dorsal fold **df10**. It is a median part of the fragmented and reduced tergite 11 (see Klass 2001 for a discussion at the insect level). The mediotergite 11 is placed upon a small lobe, the dorsal fold 11 (**df11**, the ‘supraanal lobe’), which is a median remainder of a formerly wider fold **df11**. The telson is the posterior apical part of the insect body, which is not considered as a segment because it lacks anlagen of a ganglion and of limbs. While in ‘primitive insects’ there can be structural differentiations (telsonal sclerites and lobes), in Mantodea the telson is merely a hypothetical membranous region around the anus. The anus (opening of the rectum, **re**, thus abbreviated **re-o**) is by definition located upon the telson and forms the morphological posterior tip of the body.

### 3.6 Oothecae

**Figure 20.**
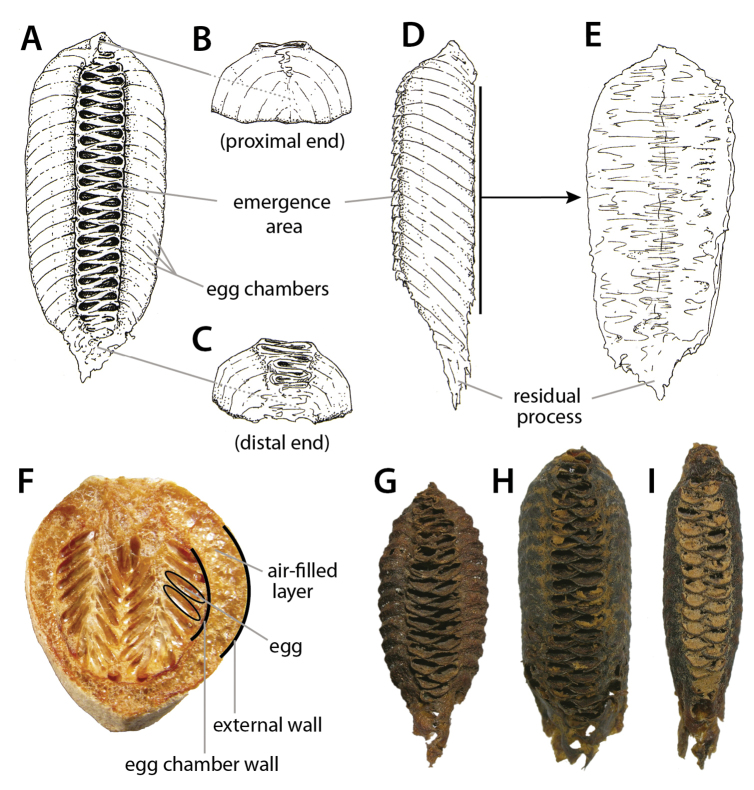
General ootheca morphology. **A**–**E** Annotated illustrations of a generic ootheca: **A** dorsal view **B** rear view **C** frontal view **D** lateral view **E** ventral view **F** internal view of a bisected egg case, species unknown. Example of interspecific variation of ootheca morphology (**G**–**I**) **G** *Choeradodis rhombicollis* Latreille, 1833 **H** *Choeradodis stalii* Wood-Mason, 1880 **I** *Choeradodis columbica* Beier, 1931. Illustrations and photographs courtesy of Hiromi Yagui.

**Figure 21.**
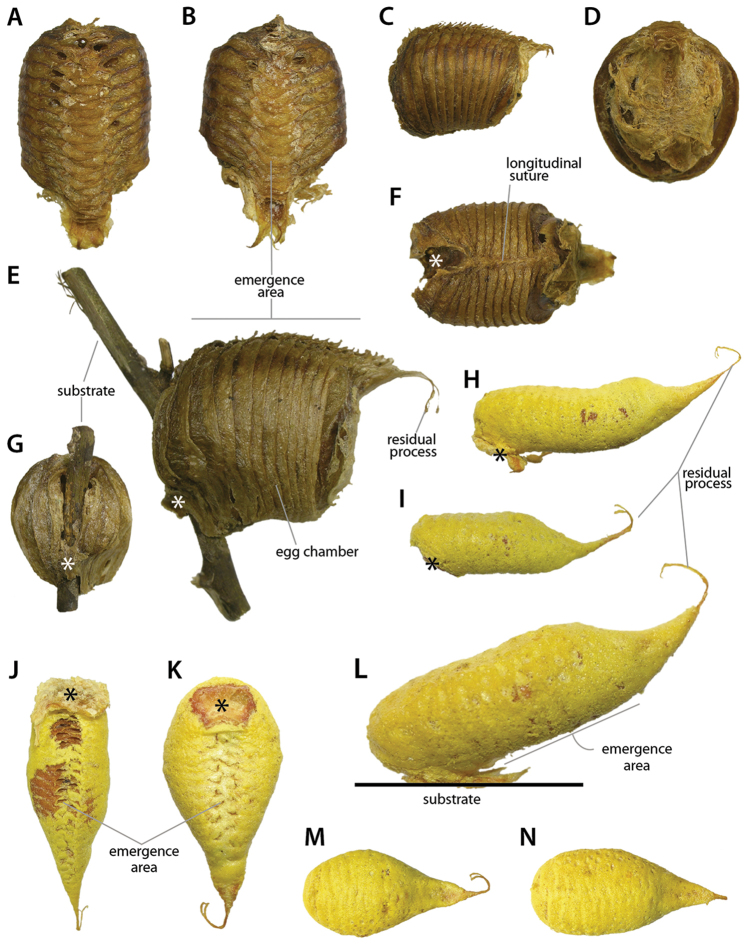
Oothecae of *Stagmatoptera supplicaria* Burmeister, 1838 and *Callibia diana* Stoll, 1813. *Stagmatoptera supplicaria* oothecae (**A**–**G**): **A**–**B** dorsal view **C** lateral view **D** frontal view **E** lateral view of ootheca attached to substrate **F** ventral view **G** rear view showing attachment site. *Callibia diana* oothecae (**H**–**N**): **H**–**I** lateral view; **J**–**K** dorsal view **L** lateral view demonstrating approximate substrate position **M**–**N** ventral view. *Callibia diana* oothecae collected and imaged herein did not retain the attachment substrate. In these situations, we recommend illustrating a line to indicate substrate position. In those oothecae with extensive outer coating, it is suggested to remove part of the coating to reveal relevant details of the external wall, such as color and/or texture; in this case **J** shows regions where the outer wall is exposed. Asterisks (*) indicate points of attachment to substrate. Photographs courtesy of Hiromi Yagui.

The ootheca, or egg case, is a complex structure female praying mantises form during oviposition to provide support and protection to eggs from environmental conditions and natural enemies (Kramer 1973) (Figs 20–21). The ootheca itself is formed from the frothy secretions of the accessory glands of the female genital complex, which gradually harden upon mixing (Hackman and Goldberg 1960, Kenchington and Flower 1969b, Fuseini and Kumar 1973, Kumar and Barnor 1974, Kramer et al. 1989, Courrent et al. 2008). Unlike other dictyopterans, mantodean oothecae are remarkable for exhibiting extensive architectural and cryptic variation, as well as variation in the mechanical properties of its constituent elements, mostly protein and calcium-based compounds (Rudall 1956, Hackman and Goldberg 1960, Kenchington and Flower 1969b, Kramer et al. 1989, Walker et al. 2012). Mantodean oothecae remain largely understudied at the chemical, micro-, and macroscopic structural levels. Breland and Dobson (1947) were the first, and virtually the only, to address the implications of the structural variation of oothecae for praying mantis taxonomy. Although their sampling was limited (10 spp.) and phylogenetically scattered, Breland and Dobson (1947) suggested that oothecae exhibit distinct species-specific characters, providing a preliminary assessment of ootheca variability for the first time. A recent revision of Acanthopoidea, the polymorphic earless praying mantises, demonstrated that ootheca characters were useful for delimiting higher-level taxa (Rivera and Svenson 2016). This study, in addition to available literature records, strongly suggests that oothecal architecture does in fact exhibit great potential for taxonomic and systematic inference in Mantodea. For this reason, we consider that oothecae constitute a relevant piece of information deserving further attention from researchers. We suggest that, when possible, oothecae should be included in species descriptions and general taxonomic treatments of Mantodea (e.g., Roy 2002a, Roy and Stiewe 2009, Svenson 2014; Tedrow et al. 2014, Rivera and Svenson 2016).

The following are relevant diagnostic features exhibited by all oothecae:

1. *Shape and size*. Shape and general architecture are relatively well conserved within members of the same family, but often it is difficult to distinguish intrageneric from intraspecific variation; overall, shape is a far better diagnostic character above species level. Oothecae can be fusiform, oblong, rectangular, guttiform, barrel-like, maraca-like, cylindrical, etc., whereas size appears to be associated with the number of eggs contained within the ootheca. Regardless of size, the overall shape of the ootheca is otherwise well conserved and thus a given female will form similarly shaped oothecae. As a result, measurements such as length and width are only partially informative; nevertheless, we suggest including referential measurements to supplement ootheca descriptions. It is also important to note that captive females that are unhealthy, starved, unmated, or lack adequate oviposition substrate within their enclosures, may form abnormal oothecae unsuitable for description and/or identification; otherwise, oothecae obtained under proper artificial breeding conditions differ little if at all from their wild counterparts

2. *External wall*. The external wall exhibits extensive variation in its mechanical properties, presumably because of variation in the constituent materials that make the ootheca. The external wall can range from smooth and flexible to textured and very strong (Fig. 20f). Ootheca coloration, generally cryptic, varies from light to very dark brown, sometimes exhibiting greenish or reddish tones. The external wall may also be coated with a layer of spongious material that partially or completely covers the ootheca. The external coating varies in thickness and toughness, with coloration that ranges from whitish to dark brown, and less often brightly colored yellow or green. The external coating tends to be caducous and as such might be partially missing or missing altogether in older oothecae found in the field or in museum collections (see Fig. 21j).

3. *Point of attachment*. Depending on the species, females attach their oothecae to specific substrates, such as stems, leaves, bark, rocks, crevices, or even in the ground. The most typical type of ootheca attachment site is along the ventral surface (Fig. 20d, e), but it may also be attached to the substrate by their proximal end (Fig. 21e, l), thus forming an angle with the substrate, rendering its ventral surface exposed. The oothecae of some species cling from a thread or are stalked, and other species form inverted oothecae, attaching them to the substrate by its dorsal aspect. The point of attachment and preferred substrate exhibit extensive variation and are diagnostic at various taxonomic levels.

4. *Egg chambers*. Oothecae basically consist of a series of egg chambers (Fig. 20a, f) aligned one after the other, usually in a zigzag arrangement. Depending on the species, oothecae are made of one to multiple egg chambers, each containing one to several eggs. The external wall delimiting the egg chamber is usually what separates the eggs from the external environment. Sometimes, a transitional air-filled layer of variable thickness separates the egg chambers from the external wall, so that a gap can be seen between the core of the ootheca containing the eggs and the external wall.

5. *Emergence area*. Each egg within the egg chamber exhibits a single opening through which each hatchling emerges (Fig. 20a). In the most common ootheca type, the chamber openings align in two parallel rows along the dorsal edge of the ootheca, forming the emergence area. However, the emergence area may exhibit modifications. For instance, it might be restricted to a single emergence opening, or the openings may exhibit a flap that the hatchlings must push to exit the ootheca. Sometimes the emergence area is sealed with a layer of spongious material, which is not retained in the other areas of the ootheca. Particularities of the emergence area are conserved at various taxonomic levels.

The following are two examples of how it is suggested oothecae should be described:

*Stagmatoptera supplicaria* Burmeister, 1838 (Fig. 21a–g). Ootheca barrel-like and mostly circular in cross-section (slightly laterally compressed). Proximal end of ootheca partially or fully encircling the substrate to which it is attached (usually a stick or stem) in such a way that the ootheca sits with its ventral surface exposed and thus the emergence area is more or less perpendicular to the substrate. External wall russet brown in color. External coating absent but, if present, it is restricted to a whitish, thin layer of frothy material on and in the immediate vicinity of the emergence area (normally absent in hatched and older oothecae). Exhibiting approximately 15–25 egg chambers whose boundaries are clearly delimited, visible on the lateral view as parallel ridges that perpendicularly converge on a longitudinal, ventral sulcus. Distal end of ootheca truncated and rough, thus differing in appearance and texture from the rest of the structure. External wall separated from central egg mass by a gap of empty space. Emergence area composed of multiple openings (at least as many as the number of chambers), all aligned to form two parallel rows along the dorsal longitudinal axis of the ootheca. Each egg chamber exhibits a flexible flap (i.e., an operculum) that closes its corresponding opening. These flaps project slightly beyond the edge of the ootheca, sometimes forming short, residual processes (often bifid). Measurements (in mm): length, 26.45–31.85; width, 20.7–20.8; thickest girth, 67.8–69; length of emergence area, 21.6–24.95; width of emergence area, 6.2–6.5.

*Callibia diana* (Stoll, 1813) (Fig. 21h–n). Ootheca guttiform (i.e., proximal end broadly rounded, distal end tapered), elliptical in cross-section, and clearly dorsoventrally compressed. Ootheca attached to flat substrates (such as leafs) by its proximal, dorsal angle and rotated at almost 110–130 degrees over its longitudinal axis relative to the substrate; thus, what appears to be the dorsal aspect of the ootheca is actually ventral, as the emergence area is in close proximity to the substrate. External wall russet brown, thick and rigid, surface coarse and ventrally scaly. External coating present and persistent, brightly colored in sulfur yellow, even in older specimens; the coating is smooth, thick and extensive, covering the entire ootheca, including the emergence area. Exhibiting approximately 24–42 egg chambers, the boundaries of which are clearly delimited and visible in lateral view (better seen after partially removing the outer coating). Emergence area with 22–39 openings that align to form two parallel rows along the dorsal longitudinal axis; emergence area itself depressed, forming a subtle longitudinal furrow. Distal end of ootheca exhibits a long, filiform, and often bifid, residual process. Measurements (in mm): length, 14.25–19.45; width, 7.4–10; thickest girth, 21.25–26.2; length of emergence area, 11.65 –20.1; width of emergence area, 2.4–3.8.

### 3.7. Specimen collection

In temperate climates of the northern hemisphere, praying mantises generally hatch between April and June with their final eclosion occurring 3 to 4 months after hatching, living for roughly 6 to 7 months in the wild (Lawrence 1992, Hurd et al. 1994, Maxwell 1999). In southern Europe, some species hatch in the summer and early autumn, overwintering as mid-instar nymphs, before eclosing to their adult forms in the spring, whereas in tropical or desert climates, oothecae can hatch throughout the year, or in association with seasonal periods, with adults living for a longer period of time in the wild than their temperate cousins (Maxwell 1999). Regional seasonality is presumed to influence the timing of life cycles (Wolda 1988, Hurd 1999).

**Figure 22.**
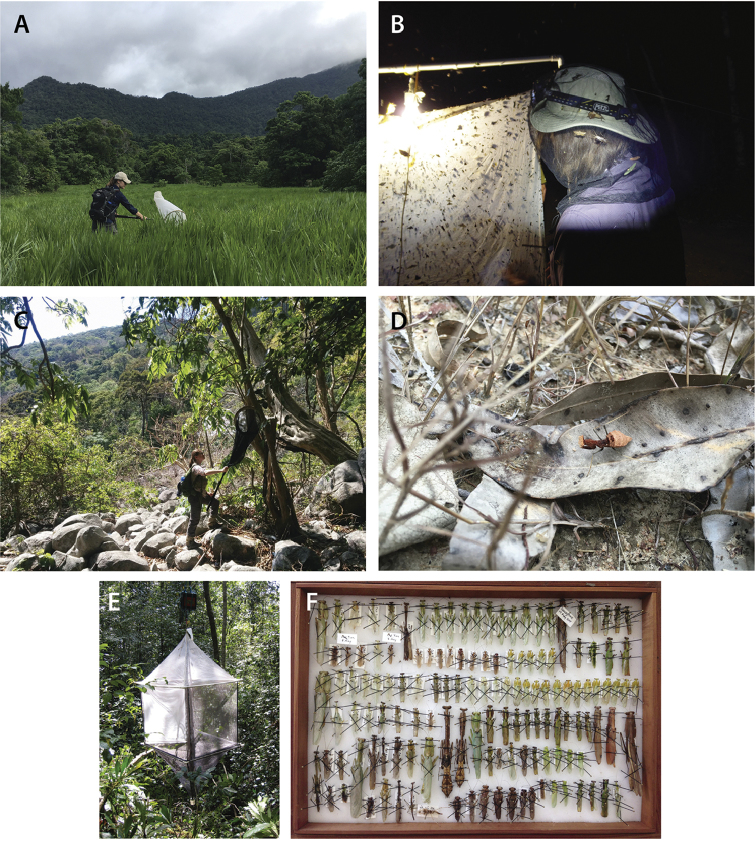
Mantis collecting techniques. **A** Sweeping vegetation with an insect net for the collection of grass-dwelling mantises **B** metal halide light trap set up to collect flight-capable mantises **C** scanning bark with the aid of an insect net for the collection of bark mantises **D** scanning the ground and leaf litter for ground-dwelling mantises **E** passive light trap set up to be suspended in tree canopies for the collection of canopy-dwelling mantises **F** field preparation of collected specimens secured with insect pins inside a Schmitt Box. It is recommended to thoroughly dry out preserved specimens in the sun, being mindful of ants and other scavengers, prior to travel back to home institutions.

Praying mantises can be collected using a wide variety of insect collecting techniques. To collect praying mantises via sweep netting, sweep an insect net back and forth over vegetation, such as bushes and shrubs, grasses, and the leaves on tree branches (Fig. 22a). However, care must be taken to check the net bag frequently as too much vegetation and debris can become compacted in the bottom of the net, potentially damaging sampled specimens. Researchers can also employ drop cloth sampling using a white ground sheet or a handheld beat sheet, which is a sheet that is held open with two support rods that are arranged into an “X” and fitted into the four corners of the sheet. These sheets are placed strategically under the foliage of small trees and shrubbery prior to beating the vegetation to dislodge insects, which then land on the sheets where they can be easily seen and hand collected. This method is effective in open habitats, such as savannahs with sparse, woody vegetation. Light trap methods entail suspending metal halide, mercury vapor, UV, or LED lights in front of a vertical white sheet (Fig. 22b) or within a passive trap system. It is recommended to frequently check the light trap for target insects and to survey around the area of the set-up as it is common to find praying mantises in the periphery of the light. To locate bark mantises, slowly run an insect net or long stick down the posterior surface of tree trunks while visually scanning the surface of the trunk for the movement of mantises (Fig. 22c). When disturbed, bark mantises tend to run around the circumference of the tree, rarely leaving the trunk (Hill et al. 2004). It has been observed that generally only one bark mantis will occupy the surface of a given tree (O’Hanlon 2011). For the collection of ground mantises, slowly walk within habitats with patchy vegetation and light leaf litter, or within desert or rocky habitats, gently moving the substrate with an insect net or boot while carefully watching for the movement of quick running ground mantises (Fig. 22d). Other techniques, including flight intercept or canopy light traps (Fig. 22e), have been known to aid in the collection of mantises as well.

To document live specimens with photo- and videography in order to capture information about body position, coloration, and behavior, living specimens can be collected into individual 50 mL vials for temporary storage. The specimens can then be individually placed (to prevent cannibalism) into a pop-up mesh enclosure for long-term housing. For euthanization, place collected specimens into a kill jar activated with cyanide or ethyl acetate. If necessary, the right mesothoracic leg or thoracic muscle tissue can be removed post-euthanization and preserved in 95% ethanol for future DNA tissue vouchering or in RNA*later*™ for RNA and mRNA isolation. Lastly, field store specimens in vials of 95% ethanol (especially early instar nymphs), pinned within a Schmitt Box and secured with multiple bracing pins, or individually wrapped in tissue paper or glassine envelopes with chlorocresol crystals added to the storage container to preserve specimens. If field pinning (Fig. 22f), it is critical to dry the specimens as soon as possible, which can be accomplished using the sun or a specifically designed field drying box with a light bulb. Furthermore, if field pinning specimens within a Schmitt Box, it is highly recommended to store the entire box within a tightly sealed plastic bag or some similar means because ants will be able to detect the deceased mantises and enter the box to harvest food, thereby destroying them. If using chlorocresol, monitor specimens regularly for damage from the chlorocresol tablets and store in cool, dry place.

### 3.8. Specimen preparation

Pinning and spreading insect specimens is a delicate process, which involves some degree of scientific forethought. The joints of specimens become rigid and brittle after death, and with this in mind, specimens need to be properly prepped for mounting. Place dried specimens in a relaxing chamber from 6 hours to two days (depending on specimen size), frozen specimens need to be thawed, and specimens preserved in ethanol need to be rehydrated by transitioning through a sequence of successively lower ethanol concentrations over several days (i.e., from 95% ethanol to 80%, 70%, 50%, 30%). These relaxing techniques should make specimens pliable enough to pin and spread (see specimen mounting section). Large-bodied specimens, such as gravid females, might preserve better if the abdominal cavity is eviscerated and stuffed. To do this, cut along the pleural membrane of one side of the abdomen with a scalpel, between the tergum and sternum. Next, carefully remove the internal contents with forceps, being careful not to destroy or remove the genitalia. To preserve the eviscerated specimen, lightly dust inside the abdominal cavity with a mixture of 3 parts talcum powder to 1 part boracic acid. If desired, the abdominal cavity can be stuffed with cotton to restore shape (Ehrmann 2002, Gullan and Cranston 2010). It is strongly recommended that specimens intended for research not be eviscerated as this technique can destroy internal and external morphological characters, ultimately rendering specimens difficult to describe for taxonomic purposes.

Early instar nymphs should be stored in ethanol, as they tend to desiccate irreparably. Subadults, especially those of large species, can be spread fairly well.

It is recommended that exuviae be stored individually in small cellophane bags, mounted on the pin of the specimen that molted it, if possible. Exuviae can also be stored individually in vials of 95% ethanol.

Oothecae do not require special treatment for preservation, and thus common insect pinning techniques can be used for these structures. Oothecae are better preserved dry, pinned through their proximal end. It is recommended to collect wild oothecae along with the substrate to which they were attached, as this provides useful information for taxonomic identification and description. For embryological research, store oothecae in 95% ethanol, which will adequately preserve the egg case and embryos.

### 3.9. Specimen mounting

Euthanized praying mantis specimens that are to be mounted for curation or vouchering need to first be relaxed in a humidifying chamber ranging for 6 hours (specimens under 22mm) to 2 days (completely dry, large bodied specimens). To ensure that the specimen does not decay, check the humidifying chamber periodically. Pin and spread specimens on a foam insect pinning board using size 3 insect pins (or size 1–2 insect pins if mounting small and delicate specimens, e.g., species of *Amorphoscelis, Hapalopeza*, *Mantoida*, etc.) and a mounting block. Symmetrically arrange and brace the appendages using appropriately sized insect pins. Spread and position the right fore- and hindwings parallel to each other using either size 000 pins or glassine paper secured with pins. Due to the extreme morphological diversity of praying mantises, some slight modifications to the following pinning methods might need to be employed to minimize specimen damage and enhance ease of mounting.

1.) With mantis in hand, gently flex and move the fore-, meso-, and metathoracic legs, ensuring that the specimen can be positioned complanate on the pinning board without sustaining damage.

**Figure 23.**
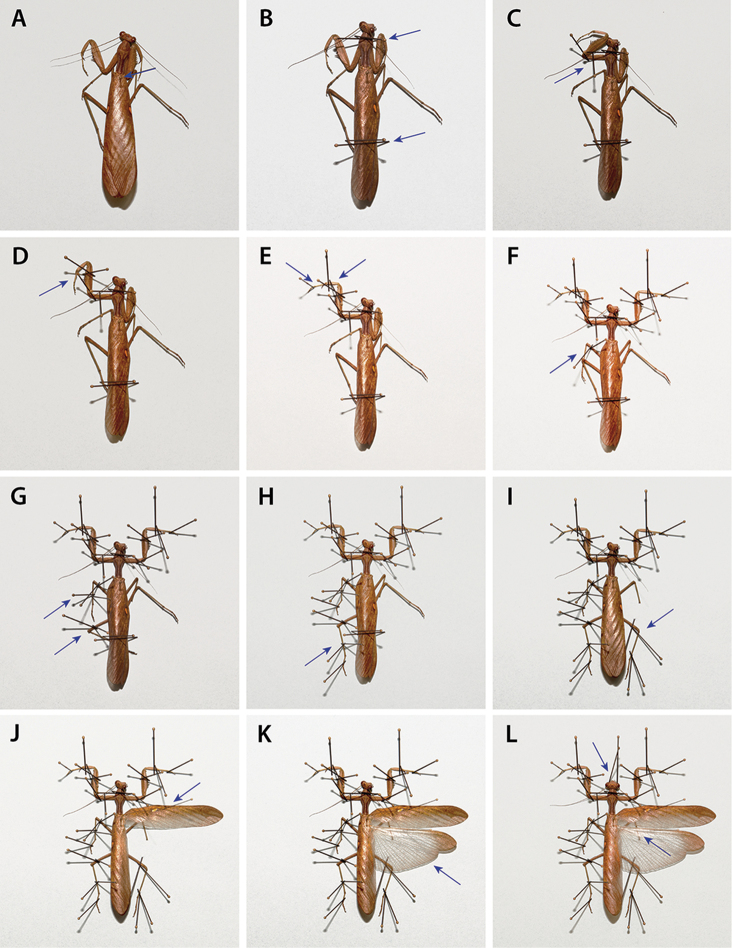
Pinning demonstration of a relaxed *Sphodromantis* sp. specimen. Blue arrows indicate approximate locations to arrange and/or pin specimen. **A** Pin specimen to the right of the midline in the mesothorax, just above the forewings **B** insert pins across the prozone and abdomen to support the specimen while pinning **C** position and secure the forecoxae **D** position and secure the forefemora **E** position and secure the foretibiae and foretarsi **F**–**I** position and secure the left mid- and hindleg as well as the right hindleg; the right midleg needs to be arranged but not secured with pins to accommodate the wings (see **J**) **J** position and secure the right forewing **K** position and secure the right hindwing **L** if needed, make further adjustments to the position of the head, antennae, and right mesothoracic leg to ensure bilateral symmetry. Due to the morphological diversity of praying mantises, some modifications to this method might need to be employed to minimize specimen damage.

2.) Place the specimen on the mounting board, dorsal side up. Pin the specimen to the board by inserting a pin to the right of the midline in the mesothorax, just above the forewings (Fig. 23a). The specimen should be roughly 25 mm from the pointed tip of the pin. A precise height can be obtained by using an insect pinning block. Pinning the specimen at this height enables one to grasp the pin without risk of touching the specimen and ensures that curated collections look uniform.

3.) To stabilize the specimen during the spreading process, cross two bracing pins anterior to the supracoxal sulcus of the pronotum and another pair above the abdomen (Fig. 23b). This will reinforce the specimen’s body, thereby minimizing the likelihood of damage that could occur by arranging the forelegs.

4.) Move the forecoxae forward, arranging them symmetrically and perpendicularly to the body. Secure this position with bracing pins (Fig. 23c).

5.) Position the forefemora symmetrically and perpendicularly to the forecoxae. Ensure that the foretibiae are directed laterad. Secure this position with bracing pins (Fig. 23d).

6.) Position the foretibiae symmetrically and perpendicularly to the forefemora. Secure this position with bracing pins (Fig. 23e).

7.) Position the foretarsi along the same axis as the foretibiae, perpendicular to the forefemora. Secure this position with bracing pins (Fig. 23e).

8.) Position the left mesofemur anteriorly, at an approximate 60° angle to the mesothorax, with the left mesotibia and mesotarsus positioned approximately parallel to the body (Fig. 23f–g). Secure this position with bracing pins. The same arrangement can be performed on the right mesothoracic leg if the wings are to be left unspread. However, if the right fore- and hindwings are to be spread, the right mesothoracic leg can be positioned similarly to the left mesothoracic leg, but it might not be possible to secure the leg with bracing pins.

9.) Position the metafemora posteriorly at an approximate 120° angle to the metathorax, with the metatibia angled slightly inward and the metatarsi positioned approximately parallel to the body (Fig. 23g–i). Secure this position with bracing pins.

10.) At a point located immediately behind the subcostal vein of the right forewing, slightly more proximal than distal to the body, insert a 000 insect pin and gently sweep the forewing forward to a position perpendicular to the body. Ensure that the pin tip is angled low (i.e., not perpendicular to the complanate specimen) and that the wing is rotated upwards in a consistent arc to minimize damage to the cells of the wing. With the pin still in the wing, gently insert the pin into the mounting board to secure the position of the wing (Fig. 23j). Another approach is to use strips of glassine paper over the wings, moving the wings into position as one would with Lepidoptera, before pinning into the glassine paper around the outer perimeter of the wings in order to secure them.

11.) Use the tip of a 000 pin at a point immediately behind the subcostal vein of the hindwing, slightly more proximal than distal to the body, to gently draw the wing forward in a smooth arc. Pin the hindwing just behind, but parallel to, the forewing with no overlap (Fig. 23k). The hindwing can also be secured with glassine paper as described in step 10.

12.) Working with forceps underneath the spread wings, gently position the right mesothoracic leg to achieve relative symmetry with the left mesothoracic leg (Fig. 23l). A pin might be necessary to secure the positioning of the leg, if possible.

Position the head symmetrically in relation to its body. This can be done by applying pressure with bracing pins along various points on the head. Secure this position with bracing pins. Position the antennae symmetrically with pins, preferably directed posteriorly to protect the antennae from damage and to save space in collections. For large-bodied specimens, support the abdomen by placing bracing pins underneath to prevent sagging.

### 3.10. Male and female genitalia preparation

For viewing and studying the genitalic complex, it is suggested to employ previously optimized methods for musculature or sclerotized and membranous structures (see below). Freshly euthanized specimens or those that have been preserved in ethanol or some other fixative provide the best morphological results. For dry preserved specimens, relax the specimen in a humidifying chamber for 6 hours to 2 days (depending on size) prior to dissecting the terminalia.

To investigate the sclerites and membranes of either the male or female genital complex, one first needs to dissect the terminalia at the apical margin of coxosternite 7, thereby isolating the terminal abdominal elements. A less destructive method for removing male genitalia entails cutting along the abdominal pleura from the abdominal apex to the apical margin of coxosternite 7, as well as to cut the membrane underneath the paraprocts. To remove the genital complex from coxosternite 9 (= subgenital plate), cut tissues between the genitalia and the abdominal wall (Rodrigues and Cancello 2016). To study musculature, dissect the terminalia at coxosternite 7 and soak the isolated material in 75% isopropanol for at least three days (Klass 1998).

Place the isolated terminalia in a vial of 10% potassium hydroxide (KOH) solution, heating the solution in a water bath ranging from 40°C for small specimens (e.g., *Ilomantis* Giglio-Tos, 1915) to upwards of 100°C for medium to large specimens (e.g., *Sphodromantis*) for five minute intervals, until the terminalia are sufficiently cleared of soft tissues (generally between five to thirty minutes). To clear the female genitalia of large bodied species, it might be necessary to gently remove and dislodge some internal tissues within the terminalia at some point during the maceration process in order for the solution to fully penetrate the structures. When the structures are sufficiently cleared, remove the terminalia from the KOH solution, rinse twice with distilled water, and place in ethanol to stop the clearing process. While in ethanol, clean the genitalia of remaining tissues and disarticulate the structures under a microscope with fine forceps and iris scissors (as in Klass 1997, 1998, Brannoch and Svenson 2016b, Rodrigues and Cancello 2016).

Lactic acid can be used in the place of KOH, as it is less caustic than KOH and is reportedly better at maintaining the shape of the genital structures (Triplehorn and Johnson 2005).

We strongly suggest that prepared male and female genitalia be stored in microvials of glycerin attached to the specimen’s pin for easy association. While slide mounting male genitalia is possible, we find that it creates curatorial issues (e.g., associating genital slides with specimens, storage of slides, etc.) and inhibits future morphological investigations as specimens are mounted flat onto the glass and fixed, making it impossible to study the 3-dimensional shape and subsequent characters of the genitalia. And while it might seem easier to image genitalia that have been slide mounted, characters that are not immediately visible or the shape of structures that are projected outward are lost by the compression of the slide and thus cannot be imaged. To image male and female genitalia that have been preserved in microvials, it is recommended to follow the techniques proposed by Su (2016), which entail floating genitalia in an ethanol-based gel. This technique does not interfere with image stacking or specimen preservation.

### 3.11. Specimen labels

Specimen labels should contain relevant collection information for the purposes of scientific utilization and value. The labels should be made of acid-free archival paper. The labels can be made in a spreadsheet using size 4 or 5-point font or written neatly by hand with a fine pen (e.g., 0.25 mm line width). Ink should be of archival quality, and therefore both fade- and waterproof.

The primary label should include at least the following information: country, region, GPS coordinates, elevation, date (presented as either 2.Apr.2013 or 2.iv.2013), and collector’s identity. Other information, such as habitat type and collection method, is valuable to list as well.

The secondary label should include at least the following information: taxon details (e.g., genus and species), taxonomic authority (i.e., the name of the person who described the particular taxon and the year it was formally described), and the name of the person who determined the identification.

Ootheca labels should include details on oviposition substrate, in addition to other relevant collection data (e.g., collection locality; taxonomic determination; number of hatchlings; parasitoids; habitat type; etc.). In general, preservation procedures and labeling must ensure the association of the ootheca with its collection locality, taxon and/or the specific specimen that produced a given ootheca, as well as valuable natural history information (e.g., oviposition sites, habitat use, etc.).

A technique for generationally labeling oothecae and nymphs has been described by Travassos Filho (1945) as a means for relating male-female matings, the oothecae that came of that mating, the surviving sibling imagoes that emerged from their respective oothecae, and the generation to which each mantis belongs. Serial numbers are assigned to the mated male and female (e.g., n. 80 and n. 81, respectively). The oothecae that are laid as a result of that pairing are then assigned a number and letter to denote the laying female and the order laid (e.g., 81-A, 81-B, 81-C; “81” designating the female and “A,” “B,” etc. indicating the order laid). The surviving imagoes are likewise given serial numbers beginning with the number that follows serially from their mother’s own numerical designation (e.g., 82, 83, 84, etc.). For a discussion on labeling oothecae laid by females with unknown male partners and oothecae laid by unknown females, see Travassos Filho (1945).

### 3.12. Standards of measurement

Praying mantis specimens should be mounted complanate and symmetrically in order to maximize access to morphological features, thereby minimizing the needed number of orientations to observe the specimen (see Specimen Mounting). In other words, the more irregularly a specimen is positioned, the greater the number of orientations required to observe all relevant morphological features. With advancements in digital imaging and increased specimen numbers, a standard of orientation will minimize the number of images required for digital observation in addition to streamlining workflow in the lab, thereby reducing time spent imaging specimens. With an optimally mounted specimen, researchers are able to quickly access important morphological information that will help determine sex, species, and other information of interest. Furthermore, measuring specimens that have been mounted in this way is easier and less time consuming.

*Prepared specimens.* In specimens that are “partially spread,” the wings lay flat against the thorax and abdomen. The mesothoracic and metathoracic legs are arranged symmetrically alongside the body with the forelegs held perpendicularly above the specimen’s head. The “spread” mounting technique is similar to the partially spread method, with one or both sets of wings spread. As the “spread” method best displays the specimen’s morphological features, thereby allowing for an increased ease in data collection and specimen viewing, it is recommended that this technique be employed.

*Unprepared specimens.* One of the most pervasive approaches for mounting praying mantises is the “unspread” method, that is, pinning the specimen without spreading the legs and wings. This approach allows the specimen to dry with its legs folded naturally underneath its body, making it extremely difficult to obtain morphological data without first undertaking the cumbersome process of relaxing the specimen in order to remount it.

Furthermore, because the “unspread” and “partially spread” methods often require that the specimen be more frequently manipulated for measurement collection, the specimen is at an increased risk of sustaining damage. In the following section, the process for proper measurement collection will be described and pictorially represented on specimens that have been mounted using the “spread” method. Suggestions for how to obtain certain measurements on “unspread” specimens are also included, but it should be noted that with specimens arranged in this manner it might be difficult to obtain consistent measurements.

**Head.** Arrange head with anterior surface oriented parallel to the scope bed without lateral tilting, which can result in inaccurate measurements. The anterior surface of the head should be arranged parallel to the floor of the stereo-microscope, and in full view. Based on the preservation of the specimen and the angle of the head in relation to the body, it may need to be positioned dorsally or ventrally.

**Figure 24.**
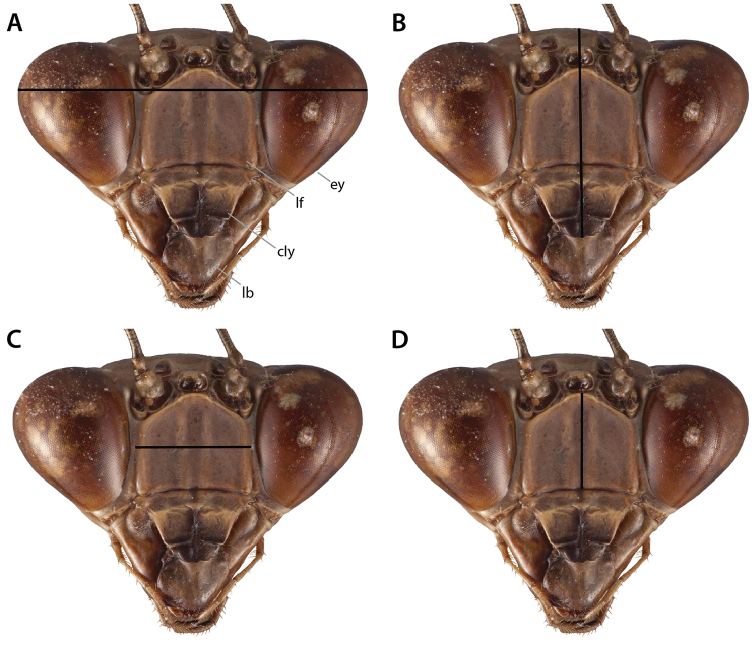
Head and lower frons measurement standards. Solid black lines indicate approximate location to take length and width measurements. *Sphodromantis* sp. head **A** head width **B** head length **C** lower frons width **D** lower frons length. Abbreviations: **cly** = clypeus; **ey** = compound eye; **lb** = labrum; **lf** = lower frons.

*Head width.* Measure from the outermost point of the compound eye to the opposing margin at the widest point (Fig. 24a). This measurement should be perpendicular to the central axis of the head.

*Head length.* Capturing the relative length of the head can be achieved by measuring from the vertex to the clypeo-labral junction. Measure from the vertex midline to the posterior margin of the clypeus, located just above the labrum (Fig. 24b). Some specimens will exhibit coloration that makes seeing this junction difficult; adjusting the light intensity or angle will render it visible.

*Frons width and length.* The lower frons is a well-defined area on the head that lies within the arms of the epicranial sulcus extending between the antennal insertions and abutting the upper margin of the clypeus. Measure from the mediolateral margin of the lower frons to the opposing margin for relative width (Fig. 24c). Measure from the anteromedial margin of the lower frons to the opposing edge for relative height (Fig. 24d).

**Figure 25.**
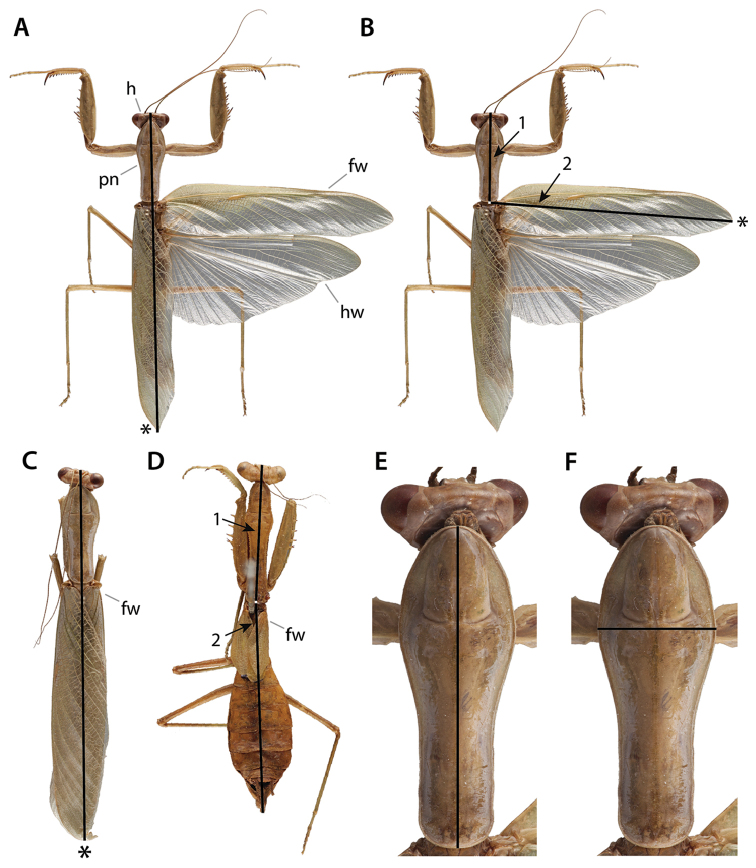
Body and pronotal measurement standards. Solid black lines indicate approximate location to take length and width measurements. *Sphodromantis* sp. prepared specimen: **A** body length measurement method 1; **B** body length measurement method 2, with measurements designated 1 and 2 indicating the first and second measurements needed for the body length measurement, which, when summed together result in total body length **C** *Sphodromantis* sp. unprepared specimen **D** *Stagmomantis nahua* Saussure, 1869 unprepared brachypterous specimen with the sum of measurements designated 1 and 2 equaling total body length. *Sphodromantis* sp. pronotal measurements **E** length **F** width. Asterisks (*) represent potential areas where specimen damage may result in an inability to take an accurate measurement (e.g., wing tip damage). Abbreviations: **fw** = forewing; **h** = head; **hw** = hindwing; **pn** = pronotum.

**Body.** Position the specimen dorsal side up, parallel to the bed of the microscope, without lateral tilting. Total length of specimen is relative to head position, abdominal preservation and wing position (Fig. 25a–d). The abdomen, being soft-bodied, is prone to desiccation and damage. Furthermore, the insect’s abdominal size and quality might vary due to its access to food sources; for female mantises, if gravid; and generally, if the terminalia have been removed for genitalic investigation. Therefore, it is suggested that measurements be taken before any dissections occur on the specimens, as it is best to have a standard measurement that is not affected by outside influences. Therefore, the distal terminus of the structure that extends farthest and is undamaged is the preferred method. Consequently, in contrast to “fixed” structures such as the pronotum or individual leg segments, overall body size is not necessarily a good character for species diagnoses or as reliable data for analysis. However, it is useful for general size ranges that may aide in rough identifications.

*Body length – Prepared specimen.* For a rough size estimate on partially spread specimens, measure from the central ocellus or medially between the antennal insertion sites as a consistent landmark that accounts for relative head position to the distal terminus of the abdomen, fore-, or hindwing (whichever is longer) (Fig. 25a–b). For a rough size estimate on fully spread specimens, measure to the middle of the meso- or metathoracic tergal plate that bears the wing axillary region and record the data and continue to the distal terminus of the abdomen, fore-, or hindwing (whichever is longer).

*Body length – Unprepared specimen.* For a rough size estimate, measure from the central ocellus or medially between the antennal insertion sites to the distal terminus of the abdomen, fore-, or hindwing (whichever is longer) (Fig. 25c–d). It might be necessary to relax and remount specimens to collect measurement data.

For comparable size determinations across taxa, it is recommended to measure from the central ocellus or medially between the antennal insertion sites and conclude at the abdominal terminus (Fig. 25d).

**Pronotum.** To measure the length and width of the pronotum, the specimen should be oriented dorsal side up, the pronotum parallel to the scope bed, without lateral tilting. For measuring the height of the pronotum, position the specimen with the lateral margin facing up (Fig. 25e–f). The pronotum is divided into a prozone and a metazone by the supracoxal sulcus, a furrow that traverses horizontally across the pronotum.

*Pronotal height, length, and width.* To determine pronotal height, measure from the anteromedial point on the lateral pronotal edge to the dorsal surface of the pronotum; the measurement should be parallel to the supracoxal sulcus. For pronotal length, measure along the midline of the pronotum from the anterior margin to the posterior margin; the measurement should be perpendicular to the supracoxal sulcus (Fig. 25e). For pronotal width, measure across the widest region of the pronotum (including lamellar expansions, see Fig. 8), from the lateral margin of the pronotum to the opposing margin (Fig. 25f).

Pronotal measurements are useful for taxonomic descriptions. If these measurements are to be included in morphological matrices for phylogenetic analysis, however, it may be useful to measure pronotal width without including the lamellar expansions in order to get comparable data throughout taxa (Wieland 2013).

**Figure 26.**
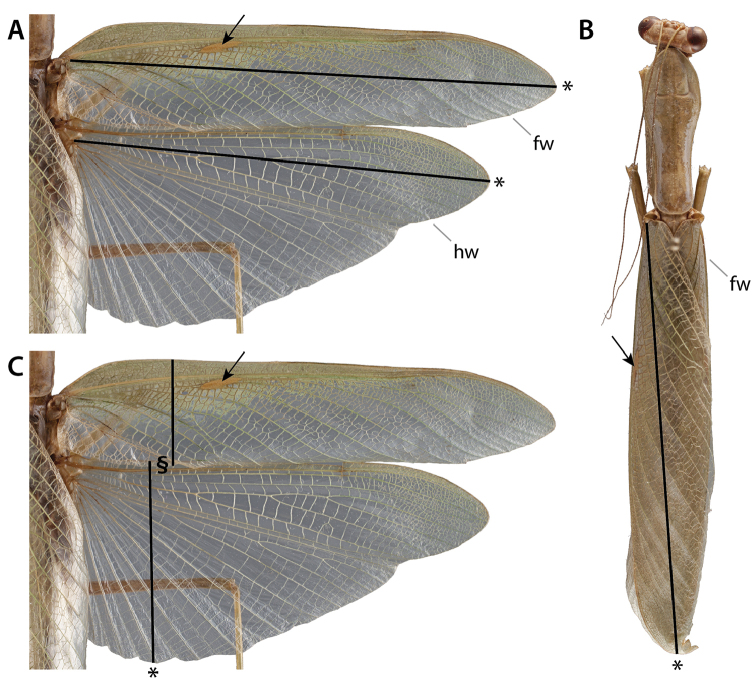
Wing length and width measurement standards. Solid black lines indicate approximate location to take length and width measurements. *Sphodromantis* sp. **A** prepared specimen fore- and hindwing length **B** unprepared specimen forewing length. Note: in unprepared specimens, hindwing length and fore- and hindwing width is impossible to measure without first relaxing and re-mounting the specimen **C** prepared specimen fore- and hindwing width. Asterisks (*) represent potential areas where specimen damage (*e.g.*, wing tip damage) may result in an inability to take an accurate measurement. Sectional sign (§) represents potential area where wing overlap can occur and either great care or re-mounting is needed to take wing width measurements. Black arrows indicate the pterostigma. Abbreviations: **fw** = forewing; **hw** = hindwing.

**Wings.** Mantodea includes species that can be macropterous, mesopterous, brachypterous, micropterous, and apterous. Wing length will be determined by the classification and condition of the wings. Position the specimen with dorsal side up, parallel to the scope bed, without lateral tilting. To ensure consistency, begin wing measurements from the point where the Analis veins converge on both the fore- and hindwings as this is a landmark feature that can be seen in both extended and folded wings (Fig. 26).

*Forewing length and width – Prepared.* To determine wing length on spread forewings, measure from the convergence of the Analis veins to the distal terminus of the forewing (Fig. 26a). For forewing width, measure across the widest point, beginning the measurement at the anterior wing margin and terminating at the distal margin (Fig. 26c).

*Hindwing length and width - Prepared.* Measure from the convergence of the Analis veins to the distal terminus of the hindwing (Fig. 26a). For hindwing width, measure across the widest point, beginning the measurement from the anterior wing margin and terminating at the distal margin (Fig. 26c).

*Forewing length - Unprepared.* Measure from the convergence of the Analis veins to the distal terminus of the wing (Fig. 26b). This measurement might be difficult to accurately obtain due to the curvature of the wing resting upon the abdomen; relaxing and remounting might therefore be necessary.

*Hindwing length - Unprepared.* Without first relaxing and remounting the specimen, hindwing length cannot be accurately obtained.

There are a few exceptional morphologies in mantodean wings, mostly due to cryptic adaptations. Among them are members of Acanthopoidea (e.g., *Acanthops* or *Pseudacanthops* Saussure, 1870; see Roy (2002b) or Lombardo et al. (2013) for examples). In the case of such aberrant morphologies, describing authors should determine how best to proceed in their undertaking of the wing measurements and make sure to describe in detail the measurements taken.

**Figure 27.**
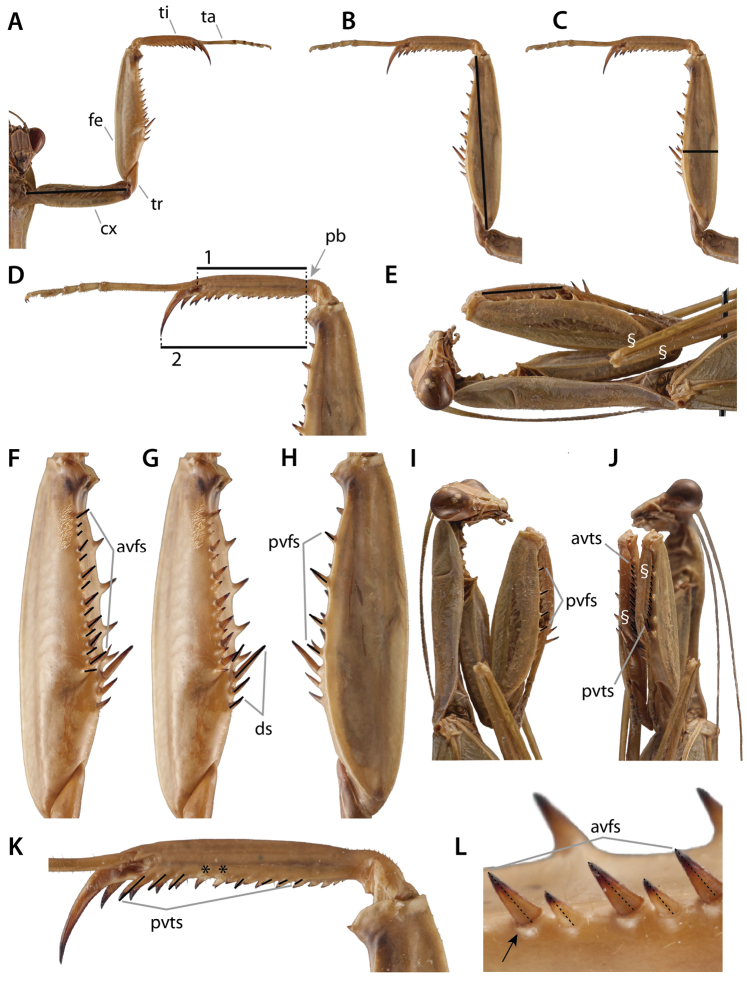
Prothoracic leg and spine measurement standards. Solid black lines indicate approximate location to take length and width measurements. *Sphodromantis* sp. prepared foreleg measurements (**A**–**D**): **A** forecoxal length **B** forefemoral length **C** forefemoral width **D** foretibial length; line 1 measures foretibial length from the proximal bend (**pb**) to the foretarsal insertion site; line 2 accounts for tibial spur length, but does not account for total spur length nor its angle; dashed black lines demonstrate approximate measurement endpoints. *Sphodromantis* sp. **E** unprepared foretibial length measurement (see note). *Sphodromantis* specimens presented in **F**–**L** are positioned for optimal viewing for the reader, not for obtaining accurate measurements. Ensure that structures are arranged appropriately before obtaining measurements. Prepared forefemoral spine length measurements (**F**–**H**): **F** anteroventral femoral spine length **G** discoidal spine length **H** posteroventral femoral spine length. Unprepared spine length measurements (**I**–**J**) (see note): **I** posteroventral spine length **J** anteroventral and posteroventral tibial spine lengths. **K** Prepared posteroventral tibial spine length measurements **L** close up view of approximate endpoints to obtain spine length measurements. Note: in unprepared specimens, obtaining certain measurements might be impossible, necessitating the remounting of such specimens. Asterisks (*) represent areas where specimen damage has occurred and sectional signs (§) represents potential areas where overlap can occur and re-mounting is needed to take measurements. Black arrow indicates cuticular margin abutting spine base. Abbreviations: **avfs** = anteroventral femoral spines; **avts** = anteroventral tibial spines; **ds** = discoidal spines; **pb** = proximal bend in the tibia; **pvfs** = posteroventral femoral spines; **pvts** = posteroventral tibial spines.

**Prothoracic legs.** The raptorial forelegs of the mantis contain extensive morphological information; spines, spurs, and denticulations adorn the forelegs of these hunters. It is important to have access to these features as they are frequently used for specimen identification (Fig. 27). To determine relative forecoxal length, arrange the specimen ventral side up, parallel to the bed of the microscope, without lateral tilting. Positioning the prepared specimen for forefemoral, foretibial and foretarsal length involves arranging the specimen dorsal side up. Position unprepared specimens to clearly expose the segments of the foreleg. With unprepared specimens, accessing the legs and performing consistent measurements presents many difficulties; relaxing and remounting the specimen might be necessary.

*Forecoxal length – Prepared.* Measure from the point of the anterior convergence of the coxal keels to the valley of the coxal lobes, near the coxo-trochanteral hinge (Fig. 27a). If the marginal lobes are not distinctly divergent, measure to the median of the distal terminus of the forecoxa.

*Forefemoral length and width – Prepared.* Measure from the proximal-most point on the forefemur, where the forefemur abuts the trochanter, to the apex of the genicular lobe (Fig. 27b). The width of the forefemur can also be taxonomically useful. To achieve forefemoral width, measure across the widest point, from the lateral margin to the opposing edge (Fig. 27c).

*Foretibial length – Prepared*. Measuring the foretibia involves taking parallel measurements that ultimately account for the total relative length of the structure. As the raptorial and cursorial tibiae serve functionally different roles, it is important to ensure that no information is lost when measuring these seemingly homologous appendages. The success of prey capture being perhaps dependent on the relative length of the foretibia and tibial spur, it is important to attend to both the length from the base of the proximal bend of the foretibia to the tarsal insertion point (Fig. 27d, measurement 1). It is likewise necessary to measure the length from the proximal bend of the foretibia to the tip of the tibial spur (Fig. 27d, measurement 2). These two measurements must be parallel to one another.

**Figure 28.**
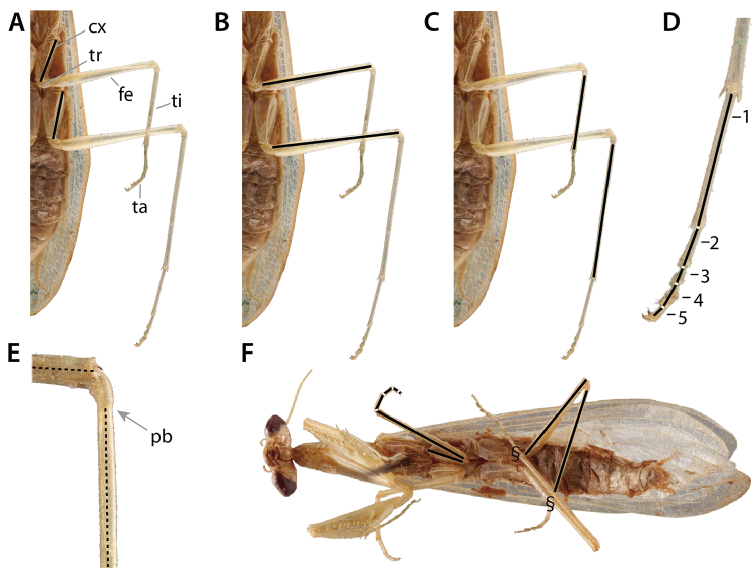
Meso- and metathoracic leg measurement standards. Solid black lines indicate approximate location to take length and width measurements. Dashed black lines demonstrate approximate locations for measurement endpoints. Prepared *Ilomantis ginsburgae* Brannoch & Svenson, 2016 meso- and metathoracic leg length measurements, ventral view (**A**–**E**): **A** meso- and metathoracic coxal length **B** meso- and metathoracic femoral length **C** meso- and metathoracic tibial length **D** tarsal length measurements with lines 1–5 indicating contiguous tarsomere measurements **E** approximate location of endpoints for the meso- and metathoracic femora and tibiae measurements. **F** Unprepared *I. ginsburgae*, ventral view, meso- and metathoracic leg length measurements. Note: in unprepared specimens, obtaining certain measurements might be impossible due to appendage overlap, thus necessitating the relaxing and re-mounting of such specimens. Sectional sign (§) represents potential area where overlap can occur and relaxation and re-mounting is needed to take accurate measurements. Abbreviations: **cx** = coxa; **fe** = femur; **pb** = proximal bend in the tibia **ta** = tarsus; **ti** = tibia; **tr** = trochanter.

*Foretarsal length – Prepared*. The tarsus should be measured using a segmented measuring tool to account for any curvature found in the positioning of the tarsomeres. Measure from the tarsal insertion point on the tibia, concluding at the distal terminus of the final tarsomere, not incorporating the ungues (see Fig. 28d). The measurement should consist of at most five contiguous measurements that begin and end at the segmentation of each tarsomere, which, taken together, will yield the relative length of the tarsus. Measuring tarsomeres can be complicated by the euplantulae, which, when elongate, can obscure the borders of each tarsal segment.

*Foreleg measurements – Unprepared.* Depending on the preservation status of the specimen, obtaining accurate measurements might not be possible without first relaxing and remounting specimens. Often, structural overlap will obscure intended measurements (Fig. 27e, note the sectional symbols § demonstrating structural overlap).

*Forefemoral spines – Prepared.* There are three rows of spines on the forefemur: posteroventral, discoidal, and anteroventral. Posteroventral spines are found on the posteroventral edge of the forefemur; the discoidal spines are found on the ventral surface of the forefemur, between the anteroventral and posteroventral rows of spines; and the anteroventral spines are on the anteroventral edge (some exceptions to this are the posteroventral spines in several species of *Paraoxypilus*, *Hoplocorypha* Stål, 1871, *Eremiaphila* Lefebvre, 1835, and *Blepharopsis* Rehn, 1902, which are also found along the anteroventral edge). There are some less common phenotypes that lack certain rows of forefemoral spines, such as the posteroventral spines in *Perlamantinae* and all of the rows of spines except for one persisting discoidal spine in *Amorphoscelinae*.

*Anteroventral forefemoral and foretibial spines – Prepared.* Arrange the specimen ventral side up, parallel to the scope bed, and without lateral tilting. Determine the relative length of the spines by measuring from the cuticular margin, the externally visible cuticular rim at the base of the spine, to the distal terminus of the spine (Fig. 27f, l). This measurement should be taken medially along the spine.

*Discoidal spines – Prepared.* With the specimen positioned ventral side up, parallel to the scope bed, without lateral tilting, measure from the cuticular margin that runs along the base of the spine to the distal terminus of the spine (Fig. 27g). This measurement should be taken medially along the spine.

*Posteroventral forefemoral and foretibial spines – Prepared.* Arrange the specimen dorsal side up, parallel to the scope bed, and without lateral tilting. To determine the relative length of each spine, measure from the cuticular margin of the spine and conclude the measurement at the distal terminus (Fig. 27h, k). This measurement should be medially centered on the spine.

*Forefemoral Spines – Unprepared.* Accessing the morphological information contained on the forefemur and foretibia of unprepared specimens might be extremely difficult to achieve, if not impossible. Even with visual access to spines, measuring their relative lengths will ultimately lack consistency across taxa because the specimen position needed to view and measure the spines will not be standardized across all specimens. It is highly recommended that the specimen be relaxed and re-mounted with forelegs spread (Fig. 27i–j, note the sectional symbols § demonstrating structural overlap).

**Mesothoracic and metathoracic legs.** Arrange the specimen ventral side up, meso- or metathoracic legs parallel to the bed of the microscope, without lateral tilting. The measurements of the meso- and metathoracic legs involve essentially the same methodology as the forelegs, with just a few slight adjustments. With unprepared specimens, inconsistency and inability to take measurements present a challenge and so it is recommended to relax and remount specimens.

*Meso-, metacoxa – Prepared.* To determine the relative length of the meso- and metacoxa, measure from the anterior convergence of the coxal keels to the distal terminus of the meso- and metacoxa, near the coxo-trochanteral hinge and along the lateral-most side of the segment (Fig. 28a).

*Meso-, metafemur – Prepared.* Measure from the point on the meso- and metafemur most proximal to the body, which abuts the trochanter, to the distal terminus of the femur. This measurement should begin along the lateral margin edge of the femur, concluding at the midline of the femoral terminus (Fig. 28b).

*Meso-, metatibia – Prepared.* Begin the measurement at the proximal bend of the meso- and metatibia (an exoskeletal indentation that serves as an attachment site for the tarsal muscles, see Fig. 28e), at a mediolateral point. Measure to the distal terminus of the meso- and metatibia, concluding the measurement at a point medially positioned between the apical spur and the cuticular outgrowth of the meso- and metatibial apex (Fig. 28c).

*Meso-, metatarsus – Prepared.* The meso- and metatarsus should be measured using a segmented measuring tool to account for any curvature found in the positioning of the tarsomeres. Measure from the tarsal insertion point on the meso- and metatibia, concluding at the distal terminus of the final tarsomere, not incorporating the ungues (Fig. 28d). The measurement should consist of at most five contiguous measurements that begin and end at the segmentation of each tarsomere, which, taken together, will yield the relative length of the tarsus. Measuring tarsomeres can be complicated by the euplantulae, which, when elongate, can obscure the borders of each tarsal segment.

*Meso- and metathoracic leg measurements - Unprepared.* Depending on the preservation status of the specimen, obtaining accurate measurements might not be possible without first relaxing and remounting specimens. Often, overlap of structures will obscure intended measurements (Fig. 28f, note the sectional symbols § demonstrating structural overlap).

**Figure 29.**
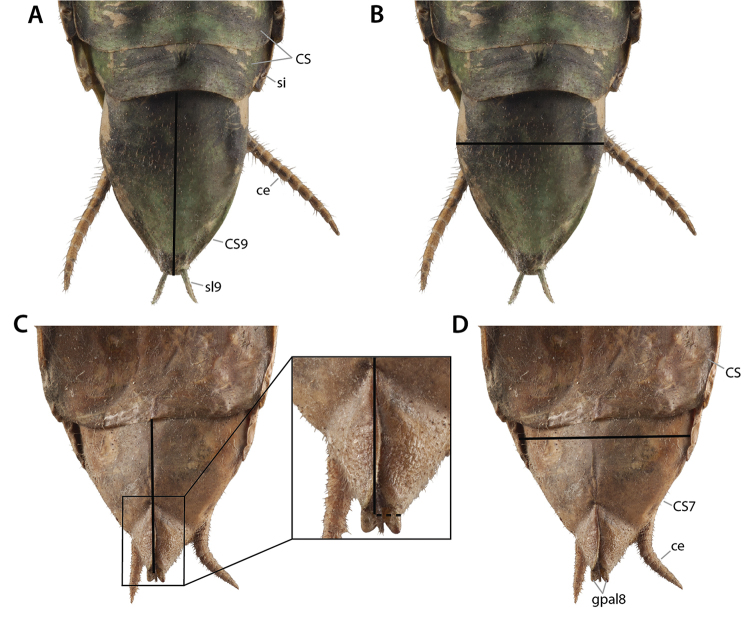
Coxosternite 7/9 (= ♀/♂ subgenital plate) length and width measurement standard. Solid black lines indicate approximate location to take length and width measurements. Dashed black lines demonstrate approximate locations for measurement endpoints. *Omomantis* sp. ♂ **CS9**, ventral view (**A**–**B**): **A** length **B** width. *Sphodromantis* sp. ♀ **CS7**, ventral view (**C**–**D**) **C** length with inset showing approximate location for length measurement endpoint **D** width. Abbreviations: **ce** = cercus; **gpal8** = apical lobe of gonapophysis 8; **CS** = coxosternite; **CS7** = ♀ coxosternite 7; **CS9** = ♂ coxosternite 9; **si** = spiracle; **sl9** = stylus.

**Subgenital plates.** Position the specimen ventral side up to view the coxosternites, specifically coxosternites 7/9 (**CS7/ CS9** = subgenital plate) to determine the sex of the specimen. There are nine coxosternites in male praying mantises, the first of which is strongly reduced and the last being **CS9**, which is usually rounded along the posterior edge and more or less asymmetrical (Fig. 29a–b). The variability of the male **CS9** was diagnosed by Wieland (2013). There are seven coxosternites in female praying mantises, the last being **CS7**, which is bifurcated and depressed near the posteromedial edge (Fig. 29c–d). The apical lobes of gonapophyses 8 (**gpal8**) may be externally visible.

*Male* ***CS9*** *Length.* Measure from the anterior margin of **CS9**, concluding medially along the terminal edge (Fig. 29a).

*Male* ***CS9*** *Width.* Measure across the widest point of **CS9**, concluding at the contralateral margin (Fig. 29b).

*Female* ***CS7*** *Length.* Measure from the anterior margin of **CS7**, concluding medially at the point where the **CS7** bifurcation concludes, not including the exposed apical lobes of **gpal8** (Fig. 29c).

*Female* ***CS7*** *Width.* Measure across the widest point of **CS7**, concluding at the contralateral margin (Fig. 29d).

### 3.13. Glossary

Morphological terms are generally listed in the singular. We indicate whether a term refers to a paired or an unpaired structure, according to the following specifications (note: the following indications refer to the usage of a term rather than to the condition of the structure concerned) – [***paired***]: term referring to a structure that occurs in two exemplars, one on the left and one on the right side of the body, with clear side-homonomy; [***unpaired***]: term referring to a structure that occurs in one exemplar that is present at the midline but may to a varied extent extend from there to the sides of the body; [***one-sided***]: term referring to a structure that occurs in one exemplar either on the left or on the right side of the body and has no or no identified counterpart on the other side; [***paired, counterpart: ...***]: term referring to a structure that occurs in one exemplar either on the left or on the right side of the body but has a differently named homonomous counterpart on the other side; [***unpaired to paired***]: term variously referring to an unpaired or a paired structure in different taxa, while it is unclear which is the plesiomorphic condition.

**Accessory gland(s)** – **ag** [= colleterial gland(s)] [*unpaired*]: ♀; a median cuticular invagination of the posterior part of segment 9, opening anteroventrally of the bases of the gonapophyses 9, consisting of a proximal pouch internally dividing in a right and a left branch, which are structurally and functionally asymmetrical.

**Accessory gland supporting lobe** – **agsl** [*unpaired*]: ♀; a posteriorly directed lobe covering the opening of the accessory glands ventrally; apex bifid, membranous or sclerotized by a sclerite **AG**.

**afa** – [*one-sided*]: ♂; left phallic complex (left phallomere), 1st process from anterior upon the edge **pba** between pouches **pne** and **lve** (with **L1b** or **L1B** sclerotization) (reference: ‘**a**pofisi **f**alloide’).

**age** – [*one-sided*]: ♂; right phallomere, sclerotized groove or deeper infolding along anterior margin of ventral wall, the deeper mesal part often curved (with **R3** sclerotization) (reference: ‘**a**nterior **g**roove’).

**Antenna** – [*paired*]: sensory appendages inserted near the ocelli on the cranium; generally filiform, sometimes moniliform, pectinate, or serrate (plural: antennae).

**Antennal sclerite** – [*paired*]: sclerotized rim around the antennal socket.

**Antennifer** – [*paired*]: the point of articulation in the base of the antennae, externally visible as a sulcus within the circumantennal sclerite (Snodgrass 1935).

**Anterior Analis** – **AA** [paired]: the vein system immediately posterior to CuP; it is composed of a simple first branch, **AA1**, and of second branch generally branched, **AA2**.

**Anterior Cubitus** – **CuA** [*paired*]: the fourth major longitudinal wing vein; the anterior branch of the Cubitus; anteriorly (e.g., *Metallyticus*) or posteriorly (e.g., *Chaeteessa*) branched in Mantodea (Béthoux and Wieland 2009).

**Anterior Radius** – **RA** [*paired*]: the second major longitudinal wing vein; the anterior branch of the Radius.

**Anterior spermathecal bulge fold** – **spba** [*unpaired*]: ♀; an external transverse fold bordering the spermathecal bulge to the anterior.

**Anterior veinal margin** – **avm** [*paired*]: vein-like structure along anterior wing margin; area anterior to the **avm** is the visor.

**Anterior wing margin** – **awm** [*paired*]: membranous area along anterior wing margin; area posterior to the **awm** is the visor.

**Anteroventral forefemoral spine(s)** – **avfs** [*paired*]: a row of spines on the anteroventral edge of the forefemora.

**Anteroventral foretibial spine(s)** – **avts** [*paired*]: a row of spines on the anteroventral edge of the foretibiae.

**Anus** – **re-o** [= **opening of rectum** (**re**)] [*unpaired*]: the posterior opening of the gut (hindgut), forming the morphological posterior tip of the body upon the telson; formed by the inwardly bending central walls of the dorsal fold 11 and the subanal lobes.

**apa** – [*one-sided*]: ♂; left phallic complex (left phallomere), 2nd process from anterior upon the edge **pba** between pouches **pne** and **lve** (with **L1b** or **L1B** sclerotization) (reference: ‘**a**pophysis, **p**osterior [process]’).

**Apex (wing)** – [*paired*]: the distal-most tip of the wing.

**Apical cleft of gonoplac 9** – **glcf9** [*paired*]: ♀; a notch in the wide apical edge of the gonoplac **gl9**, making the **gl9** apex slightly bifid.

**Apical lobe of gonapophysis 8** – **gpal8** [*paired*]: ♀; the lobe-like apex of gonapophysis 8.

**Arculus** – **arc** [*paired*]: hindwing; a strengthened cross-vein connecting media **M** and the anterior cubitus **CuA**.

**Aulax** – **al** [*paired*]: ♀; a longitudinal groove in the middle third of the dorsal wall of gonapophysis 8 that can harbor the rhachis of gonapophysis 9 to form a sliding interlock (a tongue-and-groove system), the olistheter.

**Basitarsus** – [*paired*]: the basal segment of the tarsus (plural: basitarsi).

**bm** – [*one-sided*]: ♂; right phallomere, a dextral extension (with **fda** and **R1** sclerotization) (reference: ‘**b**raccio **m**ediale del fallomero’).

**Cardo** – [*paired*]: proximal subdivision of the maxilla; articulates with the cranium (Snodgrass 1935).

**Caudogyne** – **CG8** [*unpaired*]: ♀; a median sclerite located in the anterior ventral wall of segment 8, between the anterior ends of coxae 8; possibly part of true sternum 8, perhaps the sternite 8.

**Cercus** – **ce** [*paired*]: a long filiform process having its base far laterally posterior to tergite 10 and paraproct; (part of?) the limb of abdominal segment 11.

**Cercomere** (apical cercomere **CEa**; basal cercomere **CEb)** – [*paired*]: any of the cylindrical sclerites following one after the other along a cercus.

**Cervical foramen** – an aperture between the posterior surface of the head and the prothorax, a channel between the head and the thorax for various vessels, tracts, and nerves (Gordh and Headrick 2001).

**Cervix** – region between the head and the prothorax, which features sclerotizations (Wieland 2006).

***Chroicoptera*-type** – **ccsp7** [paired]: ♀; in *Chroicoptera* the coxosternite 7 (= subgenital plate) bears two elongated ventral spines that are distinctly curved laterad; ventromedial ridges present on **cs5** and **cs6** (Wieland 2013).

**Circumantennal sclerite** – [*paired*]: a sclerite that lies between the base of the antenna and the circumantennal sulcus.

**Circumantennal sulcus** – [*paired*]: a sulcus that borders the circumference of each antennal base (plural: circumantennal sulci).

**Circumocular sulcus** – [*paired*]: a sulcus that borders the circumference of the compound eyes (plural: circumocular sulci).

**Clypeus** – [*unpaired*]: located just below the epistomal sulcus, which isolates the lower frons from the clypeus; the clypeus is separated from the labrum by a cuticular infolding; the clypeus serves as a muscular attachment site and covers the mouthparts of the insect; cuticular expansions (crests, ridges) are present in some species.

**Common oviduct** – **oc** [opening = **oc**-**o**] [*unpaired*]: ♀; non-extended, median cuticular outlet duct for eggs, opening on the far posterior ventral wall of segment 7 on the genital papilla, into the space between the papilla lobe and the epigynal lobe.

**Common oviduct opening** – **oc**-**o** [= gonopore] [*unpaired*]: ♀; opening of the common oviduct.

**Compound eyes** – **ey** [*paired*]: usually globular, surfaced with ommatidia, located laterally on the cranium.

**Coronal sulcus** – **cs** [*unpaired*]: a component of the epicranial sulcus; the coronal sulcus runs dorsomedially from the vertex of the cranium towards the ocelli, where it branches off into the postfrontal sulci; the “stem” of the epicranial sulcus complex.

**Coxa** – [*paired*]: the most proximal segment on the leg; articulates with the thorax and trochanter (plural: coxae).

**Coxa 8** – **CX8** [*paired*]: ♀; a large sclerite in the lateral ventral wall of segment 8; it represents the main part of the limb-base sclerotization.

**Coxa 9** = **CX9** [*paired, with median fusion*]: ♀; originally a pair of sclerites in the posterior ventral wall of segment 9, representing the larger posterior part of the limb-base sclerotization of segment 9; located at the anterior, lateral, and posterior base of the gonoplacs **gl9** and also extending into the lateral and mesal walls of the **gl9**, which represent the limb bases or coxal lobes. The anteromesal parts of the left and right **CX9** are medially fused and can be semi-detached by weak sclerotization from the remainder of **CX9**. **CX9** is informally categorized in an unpaired mediocoxal area (**CX9μ**) and paired basicoxal (**CX9β**), mesolobocoxal (**CX9μλ**), and laterolobocoxal (**CX9λλ**) areas; the left and right basicoxal areas **CX9β** are narrowly separated at their posterior ends.

**Coxosternite** – **CS** [= ‘sternite’] [*unpaired, partly fused from paired structures*]: the undivided ventral sclerite plate of an abdominal segment; composed (without recognizable borders) of a median (eu)sternum and paired coxae, antelaterocoxae (uncertain), and postlaterocoxae.

**Coxosternite 7** – **CS7** [= female subgenital plate] [*unpaired, partly fused from paired structures*]: ♀; the seventh abdominal coxosternite; underlies the genital structures.

**Coxosternite 9** – **CS9** [= male subgenital plate] [*unpaired, partly fused from paired structures*]: ♂; the ninth abdominal coxosternite; underlies the genital structures; can be asymmetric, generally bears styli.

**Cubital furrow** – **cf** [*paired*]: forewing; anterior-most fold delimiting the remigulum, parallel to **CuP**.

**Cubitus** – **Cu** [*paired*]: the stem giving rise to **CuA** and **CuP**, visible only at the wing base.

**dee** – [*one-sided*]: ♂; left phallic complex (ventral phallomere), a pouch that receives the opening of the ejaculatory duct, ventral to the pouch **lve**, and more or less formed from part of its ventral wall (membranous) (reference: ‘**d**uctus **e**jaculatorius [pouch]’).

**Digging spines** – [*paired*]: ♀; modified structures on the terminalia of some taxa that aid in the oviposition of oothecae.

**Discoidal spine(s)** – **ds** [*paired*]: a row of spines found mesally on the interior surface of the forefemora (between 1 and 4), between the posteroventral and anteroventral femoral spines.

**DK hearing organ** – [*paired*]: ultrasound-sensitive hearing organ; DK denotes morphology with a **d**eep groove with pronounced **k**nobs (Yager and Svenson 2008).

**DNK hearing organ** – [*paired*]: higher ultrasonic thresholds than in DK form with reduced sensitivity; DNK denotes morphology with a **d**eep groove and **n**o **k**nobs (Yager and Svenson 2008).

**DO hearing organ** – [*paired*]: ultrasound-sensitive hearing organ with reduced sensitivity; DO denotes morphology with a **d**eep **o**pen groove with knobs absent or highly reduced (Yager and Svenson 2008).

**Dorsal carina of ventral fold 7** – **vfdc7** [*unpaired*]: ♀; a low median longitudinal ridge upon the dorsal wall of ventral fold, sclerotized by part of the vestibular sclerite.

**Dorsolateral coxal lobelet** – **cxdl** [*paired*]: ♀; a posteriorly and laterally directed projection from the posterolateral part of coxa 8, located dorsolateral to the ventrolateral coxal lobelet.

**Dorsolateral segmental expansion** – **dlse** [*paired*]: a flat expansion upon the lateral part of a tergite, likely resulting from a strong elevation of (part of) the laterodorsal carina.

**Dorsomedian expansion (of dorsal fold)** – **dfme** [*unpaired to paired*]: a flat expansion resulting from a local lengthening of the dorsal fold of a segment; the transition between unpaired and paired expansions is fluent, due to the degree of development of a median notch.

**Ejaculatory duct** – **dej** [*unpaired*]: ♂; the partly cuticulized (membranous; ectodermal external part) and partly not cuticulized (mesodermal internal part) duct originating from the nymphal median invagination associated with the male genitalia and then contacting the mesodermal internal genitalia; opening upon the dorsal wall of lobe **vla** of left phallic complex, occasionally via a pouch (**dee**) (reference: **d**uctus **ej**aculatorius).

**Epicranial sulcus** – **es** [*unpaired*]: made up of a coronal sulcus and two postfrontal sulci on the insect cranium; looks similar to an inverted Y (Snodgrass 1935) (plural: epicranial sulci).

**Epigynal lobe** – **egl7** [*unpaired*]: ♀; a lobe or fold on the posterior-most part of the ventral wall of segment 7 (in genital fold area); it covers the opening of the common oviduct, located at its ventral base (= dorsal base of papilla lobe), from above; can be sclerotized dorsally by an epigynal sclerite.

**Epigyne** – **EG7** [*unpaired*]: ♀; a median sclerite in the posteriormost ventral wall of segment 7, located in the dorsal wall of the epigynal lobe.

**Epistomal sulcus** – [*unpaired*]: a sulcus that lies between the lower frons and the clypeus, anteriorly connecting the subgenal sulci (plural: epistomal sulci).

**Eremiaphilidae-type** – **vfme6** [paired]: ♀; in *Eremiaphila* and *Heteronutarsus*, coxosternite 6 features two ventral digging spines that are pointed (*Eremiaphila*) or triangular and more plate-like (*Heteronutarsus*); coxosternite 6 mostly covers the coxosternite 7 (= subgenital plate) (Wieland 2013).

**Euplantula** – **epl** [*paired*]: well-developed adhesive pads found on the ventral side of the tarsomeres (plural: euplantulae).

**Fastigial process** – **fp** [*unpaired*]: a process projected from the summit (apex) of the cranial vertex.

**fda** – [*one-sided*]: ♂; right phallomere, the main posterior lobe (with **R1** sclerotization) (reference: ‘**f**allomero di **d**estra’).

**Femoral brush** – **fb** [*paired*]: small patch of setae used for grooming; distally located on the ventral surface of the forefemur.

**Femur** – **fe** [*paired*]: the third and generally largest leg segment; fused to the trochanter at its base, articulates with the tibia; forefemora modified with spines for catching prey and a femoral brush for grooming (plural: femora).

**Flagellum** – the ultimate segment of the antennae; comprised of annuli.

**Forewing** – **fw** [*paired*]: the anterior wings; attached by axillary plates to the mesothorax.

**Furcasternite** – [*unpaired*]: a ventral prothoracic plate that includes the furcal invaginations; posterior to the postcervical plate and forecoxal insertions, anterior to the spinasternite (Levereault 1936).

**Genicular lobe** – **gl** [*paired*]: a lobe located antero- and posteroventrally on the distal-most region of the femur; featuring a spine (i.e., genicular spine) in some species.

**Genicular spine** – **gs** [*paired*]: spine that can project from the pro-, meso-, and/or metathoracic genicular lobes of the femora; not considered posteroventral and anteroventral forefemoral spines (Ehrmann 2002).

**Genital chamber** – **gc** [*unpaired*]: ♀; the space enclosed above the genital fold area of segment 7 and beneath the ventral walls of segment 8; harboring the genital papilla and the opening of the common oviduct.

**Genital pouch** – **vst+gc** [*unpaired*]: ♀; the vestibulum and the genital chamber together, i.e., the entire space enclosed above the ventral fold and the genital fold area.

**Genital fold** – **gf7** [*unpaired*]: ♀; a posteriorly directed transverse fold with a very shallow, slit-like cavity beneath it; located between vestibular sclerite **VS7** and the languette sclerite, and dorsally sclerotized by the latter; **gf7** very short except for its long processes **gfp7**; forming the ventral posterior border of genital chamber.

**Genital papilla** – **gpp7** [*unpaired*]: ♀; the papilla lobe and the epigynal lobe together.

**Glossa** – [*paired, with (partial) median division*]: the medial, terminal lobes of the labium (plural: glossae).

**Gonapophyseal sclerotization 8** – **GP8** [*paired*]: ♀; the sclerotization of gonapophysis 8; it shows a complex distribution over the walls of gonapophysis 8, and its strength varies strongly in different parts.

**Gonapophysis 8** – **gp8** [*paired*]: ♀; a long process originating at the posterior margin of sclerite coxa 8; representing a modified coxal vesicle and thus likely an endite of the limb base of segment 8; in Mantodea the bodies of the left and right gonapophyses 8 are free from each other.

**Gonapophysis 9** – **gp9** [*paired, with partial median fusion*]: ♀; a long process originating (antero)mesally from the gonoplac 9; representing a modified coxal vesicle and thus likely an endite of the limb base of segment 9. The bodies of the left and right gonapophyses 9 are fused at their very bases to form a short common stem.

**Gonoplac 9** – **gl9** [= **coxal lobe 9** = **cx9**] [*paired*]: ♀; a large posteriorly directed projection from the posterior ventral wall of segment 9, in the area sclerotized by coxae 9; representing the projection of the limb base of segment 9.

**Gonoplac basal lobe** – **glbl** [*paired*]: ♀; a membranous anteromesally directed lobe at the dorsal (posterior) base of the gonoplac.

**Hindwing** – **hw** [*paired*]: the posterior wings; attached by an axillary plate to the metathorax.

**Hypopharynx** – **hpx** [*unpaired*]: a medial lobe of the preoral cavity.

**Incisor process** – [*paired*]: sharp, toothed processes on the mandible.

**Interantennal sulcus** – [*paired*]: a sulcus between the antennal bases.

**Intercervical sclerites** – **ics** [*paired (occasionally) with median fusion*]: two well-sclerotized plates positioned at the base of the cervix, posterior to, and abutting, the lateral cervical sclerites.

**Juxtaocular bulges** – [*paired*]: flattened, bulging, or pointed elevations located between each compound eye and the corresponding parietal sulcus (Wieland 2013).

**Labial palpus** – **lbp** [*paired*]: 3-segmented appendages on the labium (plural: labial palpi).

**Labium** – [*unpaired, with median fusion*]: sclerite that forms the base of the insect mouth; bears the labial palpi, the glossae, and the paraglossae.

**Labrum** – [*unpaired*]: located just below a cuticular infolding that separates the labrum from the clypeus; the labrum is a preoral structure that covers the mouthparts of the insect.

**Languette sclerite 7** – **LG7** [*unpaired, occasionally with median division*]: ♀; a plate in the genital fold area (ventral wall of genital chamber); extending on the processes **gfp7** of the genital fold; can be medially divided.

**Lateral cervical sclerite** – **lcs** [*paired*]: two well-sclerotized plates that are positioned laterad on the cervix; they abut the intercervical sclerites at their bases.

**Lateral oviduct** – **ol** [*paired*]: ♀; the pair of outlet ducts for eggs that originate by the internal dichotomy of the common oviduct, usually mesodermal (i.e., removed by maceration via KOH).

**Left phallomere** – [*unpaired*]: ♂; the left-dorsal part of the phallic complex, putatively derived from a left-dorsal primary phallic lobe; including the sclerites **L1**, **L2**, and **L4B**, and processes arising from them.

**L1** – [*one-sided*]: ♂; left phallic complex (left phallomere), a sclerite (or its 2 subdivisions) extending over the walls of pouch **pne** (sclerite area **L1a**) and the anterior part of the edge **pba** bearing processes **afa**, **apa**, and **loa** (sclerite area **L1b**); in case of a subdivision, **L1A** is the sclerite in pouch **pne** and **L1B** is the sclerite on edge **pba** and its processes.

**L2** – [*one-sided*]: ♂; left phallic complex (left phallomere), a sclerite extending over the walls of pouch **lve** (mainly dorsal wall; sclerite area **L2l**) and process **paa** (sclerite area **L2p**).

**L4** – [*one-sided*]: ♂; left phallic complex, a sclerite (or its 2 subdivisions) extending over most of the dorsal and ventral walls; in case of subdivision, **L4A** is the sclerite in the ventral wall, **L4B** is the sclerite in the dorsal wall.

**Laterocoxa 9** – **LC9** [= gonangulum] [*paired*]: ♀; a heavy lateral sclerite in the anteriormost ventral wall of segment 9, forming several distinct articulations with neighboring sclerites (**A1**, **A2**, **A3**, **A4**); representing a small anterolateral part of the limb-base sclerotization of segment 9.

**Left (phallic) complex** – [*paired, counterpart: right phallomere*]: ♂; left phallomere and ventral phallomere together; considered to represent the left part of the phallomere complex.

***Ligaria*-type** – [*paired*]: ♀; in *Ligaria*, *Ligariella* Giglio-Tos, 1915, *Parentella* Giglio-Tos, 1915, and *Entella* Stål, 1877 two dorsally-pointing, bipartite sclerotized hooks originate from gonapophyses VIII (**gp8)** and protrude from the genital chamber (Wieland 2013).

**loa** – [*one-sided*]: ♂; left phallic complex (left phallomere), 3rd process from anterior upon the edge **pba** between pouches **pne** and **lve** (membranous or with **L1b** or **L1B** sclerotization) (reference: ‘**lo**bo membranoso’).

**Lower frons** – [*unpaired*]: a sclerite located on the head, just below the antennae and in between the compound eyes; can exhibit cuticular depressions or expansions (crests, horns) in some species.

**lve** – [*one-sided*]: ♂; left phallic complex (left and ventral phallomere), the ventral pouch invaginated from the right side of the left phallic complex (with **L2** sclerotization; dorsal wall belonging to left phallomere, ventral wall belonging to ventral phallomere) (reference: ‘**l**amina **v**entrale’).

**Mandible** – [*paired*]: strong, gnathal appendages; not bilaterally symmetric.

**Maxilla** – [*paired*]: jaw-like appendages; bears the lacinia, the galea, and the maxillary palpi; the cardo, the basal-most segment of the maxilla articulates with the cranium (plural: maxillae).

**Maxillary glossa** – [*paired*]: the medial lobe of the maxilla; the stipes bears the muscle attachment site for the maxillary paraglossae (Snodgrass 1935) (plural: maxillary glossae).

**Maxillary palpus** – **mxp** [*paired*]: 5-segmented appendages located on the stipes; anterior to the labial palpi (plural: maxillary palpi).

**Maxillary paraglossa** – [*paired*]: the lateral lobe of the maxilla; the stipes bears the muscle attachment site for the maxillary paraglossae (Snodgrass 1935) (plural: maxillary paraglossae).

**Media** – **M** [*paired*]: the third major longitudinal wing vein; extant Mantodea feature a composite posterior radius (**RP**) and media (**M**) stem in the forewing (Béthoux and Wieland 2009).

**Medial ocellar process** – **mop** [*unpaired*]: a medially positioned cuticular projection that originates within the limits of the postfrontal sulci, posterior to the ocelli.

**Medial tine of gonapophysis 9** – **gptm9** [*paired*]: ♀; a posteriorly directed finger-like process formed by the distal end of the rhachis.

**Mediotergite 11** – **TGm11** [= ‘epiproct’] [*unpaired*]: a small, weak median sclerite placed posterior to tergite 10, but more or less overfolded by dorsal fold 10; most likely representing a median fragment of tergite 11.

**Medial outgrowth of gonapophysis 8** – **gpmo8** [*paired*]: ♀; a ventromesal membranous lobe near midlength of gonapophysis 8.

**Mentum** – [*unpaired*]: the distal plate of the labium, divided into anterior and posterior portions.

**Mesal bulge of gonapophysis 8** – **gpmb8** [*paired*]: ♀; a rounded bulge at the mesal base of each gonapophysis 8.

**Mesal coxal lobelet** – **cxml** [*paired*]: ♀; a posteriorly and mesally directed projection from the posteromesal part of a coxa 8.

**MESO hearing organ** – **MESO** [*paired*]: hearing organ in the mesothorax; sensitive to low frequencies but not ultrasound (Yager and Svenson 2008).

**Metazone** – **mz** [*unpaired*]: the posterior division of the pronotum, separated from the prozone by the supracoxal sulcus; generally longer than the prozone, of similar length only in a few Mantodea species (Wieland 2013: p. 55).

**Middorsal carina** – [*unpaired*]: a longitudinal external ridge (= carina) or keel at the dorsal midline of a segment (on the tergite).

**Midventral carina** – [*unpaired*]: a longitudinal external ridge (= carina) or keel at the ventral midline of a segment (on the coxosternite).

**Molar ridge** – [*paired*]: a sharp ridge on the mesal surface of the mandible (Levereault 1936).

**MSMT “hearing organ”** – **MSMT** [*unpaired*]: functionless “hearing organ” with both the meso- and metathoracic segments morphologically similar; no auditory chamber present (Yager and Svenson 2008).

**Occipital foramen** – [*unpaired*]: an opening on the posterior surface of the head capsule, which allows for the ventral nerve cord, dorsal vessel, and tracheal system to extend from the head into the thorax (Gordh and Headrick 2001).

**Occipital sulcus** – [*unpaired*]: a transverse sulcus on the posterior surface of the head capsule that terminates on the posterior articulation of the mandibles (Gordh and Headrick 2001).

**Occiput** – [*unpaired*]: the region between the vertex and the posterior opening (occipital foramen) on the posterior surface of the head capsule (Gordh and Headrick 2001).

**Ocellar process** – [*paired*]: bilateral cuticular projections that originate within the limits of the postfrontal sulci, posterolateral to the ocelli.

**Ocellar tubercle** – [*unpaired*]: an elevated tubercle or knob upon which the ocelli sit; frequently observed in males.

**Ocellus** – [*unpaired*]: three simple eyes located in between the compound eyes, posterior to the antennae; maybe be elevated by an ocellar tubercle (plural: ocelli).

**Non-visual elongations** – **nve** [*paired*]: a spine-like elongation present on the compound eyes of some species; presumably non-visual due to a lack of ommatidia on the surface of the structure (Wieland 2013).

**Olistheter** – **al+rh** [*paired*]: ♀; the sliding tongue-and-groove interlock formed by the dorsal aulax groove on gonapophysis 8 and the ventral rhachis carina on gonapophysis 9.

**Ootheca** – ♀; egg mass that is encapsulated by a hardened protein matrix that insulates, protects, and camouflages the eggs inside (plural: oothecae).

**paa** – [*one-sided*]: ♂; left phallic complex (left phallomere), 4th process from anterior upon the edge **pba** between pouches **pne** and **lve** (with **L2p** sclerotization) (reference: ‘**p**rocesso **a**picale’).

**Papilla lobe** – **ppl7** [*unpaired*]: ♀; a membranous lobe on the far posterior part of the ventral wall of segment 7 (in genital fold area); supports the opening of the common oviduct, located at its dorsal base, from below; the apex can be bilobate.

**Paraglossa** – **pg** [*paired, with (partial) median division*]: the lateral, terminal lobes on the labium (plural: paraglossae).

**Paraproct** – **PP** [*paired*]: a pair of large ventral sclerites located posterior to the male or female genitalia; their posterior parts are located on the subanal lobes; interpretation unresolved.

**Paratergal area** – **TGπ** [*paired*]: the ventrally bent lateral parts of a tergite.

**Parietal sulcus** – [*paired*]: sulcus that runs from the occipital areas toward the frontal sulcus (plural: parietal sulci).

**pba** – [*one-sided*]: ♂; left phallic complex (left phallomere), the four processes **afa**, **apa**, **loa**, and **paa** together plus the edge (between pouches **pne** and **lve**) from which they arise (with **L1** and **L2** sclerotizations) (reference: ‘**p**rocess-**b**earing’).

**pda** – [*one-sided*]: ♂; left phallic complex (ventral phallomere), a process upon the posterior edge of lobe **vla**, can be deeply bifurcate (with **L4** or **L4A** sclerotization) (reference: ‘**p**rocesso **d**istale’).

**Pedicel** – [*paired*]: second segment of the antennae.

**Phallomere complex** – [= **phallic organs** = **male genitalia**] [*unpaired*]: ♂; left phallic complex (including left phallomere and ventral phallomere) and right phallomere together; all elements derived from the nymphal primary phallic lobes around the anlage of the ejaculatory duct (see Snodgrass 1957).

**pia** – [*one-sided*]: ♂; right phallomere, a process (of varied shape) arising from the midlength to posterior right ventral wall, posterolateral to process **pva** (with **R1** sclerotization, area **R1v**) (reference: ‘**pi**astra ventrale’).

**Planta** – small sclerite ventrodistally located on the median flexor plate of the pretarsus (Gordh and Headrick 2001) (plural: plantae).

**Plica prima anterior** – **ppa** [*paired*]: fold in the wing posterior to the cubital fold but anterior to the plica prima posterior; posteriorly delimits the preplicatum; anteriorly delimits the plicatum (reference: ‘anterior primary fold’ in Latin).

**Plica prima posterior** – **ppp** [*paired*]: fold in the wing posterior to the plica prima anterior; posteriorly delimits the plicatum; anteriorly delimits the plicatulum (reference: ‘posterior primary fold’ in Latin).

**Plicatulum** – [*paired*]: area posterior to the cubital fold, delimited anteriorly by the plica prima posterior and posteriorly attached to the thorax; positioned posterior to the plicatum and the plicatulum.

**Plicatum** – [*paired*]: area posterior to the cubital fold, delimited anteriorly by the plica prima anterior and posteriorly by the plica prima posterior, which acts as a concave hinge; positioned posterior to the plicatum and anterior to the plicatulum.

**Prementum** – **prmt** [*unpaired*]: the distal portion of the labium, delimited posteriorly by the mental sulcus, the anterior portion of the prementum bears the glossae, paraglossae, and palpi of the labium.

**Preplicatum** – [*paired*]: area posterior to the cubital fold, delimited anteriorly by the cubital fold and posteriorly by another fold, the plica prima anterior; positioned anterior to the plicatum and the plicatulum.

**pne** – [*one-sided*]: ♂; left phallic complex (left phallomere), the dorsal pouch invaginated from the right side of the left phallic complex (with **L1a** or **L1A** sclerotization; with opening of phallomere gland, see Suppl. material 10: Extended abdominal glossary) (reference: ‘**p**rocesso a**n**teriore,’ but is not a process).

**Post frontal sulcus** – **pfs** [*paired*]: a component of the epicranial sulcus; the two frontal sulci branch off from the coronal sulcus at a point above the ocelli and frame the lower frons; the “arms” of the epicranial sulcus complex (plural: post frontal sulci).

**Postcervical plate** – [*unpaired*]: a plate on the sternum that exhibits great variability in length between genera; anterior to the forecoxal insertions and the furcasternite (Wieland 2013).

**Posterior Cubitus** – **CuP** [*paired*]: the fourth major longitudinal wing vein; the posterior branch of the Cubitus.

**Posterior Radius** – **RP** [*paired*]: the second major longitudinal wing vein; the posterior branch of the Radius.

**Posterior Subcosta** – **ScP** [*paired*]: the first major longitudinal wing vein; the posterior branch of the Subcosta.

**Posterior spermathecal bulge fold** – **spbp** [*unpaired*]: ♀; an external transverse fold bordering the spermathecal bulge **sbu** to the posterior.

**Posterior Subcosta** – **ScP** [*paired*]: the first major longitudinal wing vein; the posterior branch of the Subcosta.

**Posteroventral femoral spine(s)** – **pvfs** [*paired*]: a row of spines on the posteroventral margin on the forefemora.

**Posteroventral tibial spine(s)** – **pvts** [*paired*]: a row of spines on the posteroventral margin of the foretibiae.

**Postgena** – **pge** [*paired*]: the lateral, ventral area located posterior to the gena; flanked by the parietal sulcus, the transverse sulcus, and the postoccipital sulcus (plural: postgenae).

**Postocellar process** – **pop** [*unpaired*]: a cuticular projection arising from the medial region of the cranial vertex.

**Prementum** – **pmtm** [*paired*, *with (partial) median division*]: the plate on the labium that serves as an insertion point for the muscles of the palpi, glossae, and paraglossae (Snodgrass 1935).

**Pretarsus** – [*paired*]: the apical, terminal segment of the tarsus; consists of the unguitractor plate, planta, and ungues in nearly all extant insects (otherwise not discernible as a tarsomere) (pleural: pretarsi).

**Process of genital fold** – **gfp7** [*paired*]: ♀; a process upon the genital fold **gf7**, being part of **gf7**; largely sclerotized by the languette sclerite.

**Processes on the juxtaocular bulges** – **jop** [*paired*]: cuticular expansions that can originate from the juxtaocular bulges on some species.

**Pronotum** – [*unpaired*]: elongated dorsal plate of the prothorax; with or without lateral expansions, tubercles, denticulations, and conical processes (plural: pronota).

**Prozone** – **pz** [*unpaired*]: the anterior division of the pronotum, separated from the metazone by the supracoxal sulcus; generally shorter than the metazone, of similar length only in a few species (Wieland 2013: p. 55).

**Pterostigma** – **sti** [*paired*]: an oft-colored, callused, and ellipsoid region found in forewings. Present in all Mantodea, but not always prominent.

**pva** – [*one-sided*]: ♂; right phallomere, a process (of varied shape) arising from the midlength of the ventral wall, anteromesal to process **pia** (with **R1** sclerotization, area **R1t**) (reference: ‘**p**rocesso **v**entrale sclerificato’).

**R1** – [*one-sided*]: ♂; right phallomere, a sclerite (or its 3 subdivisions) extending over the dorsal and right ventral walls of lobe **fda**, including processes **pia** and **pva** in ventral wall; **R1** includes the regions **R1d** (in dorsal wall of lobe **fda**), **R1v** (ventrally, on process **pia**), **R1t** (ventrally, on process **pva**), and **R1c** (centrally between the other areas, bearing articulation **R1-R3** labeled **A3**); in case of a subdivision, **R1A** is the sclerite in the dorsal wall of lobe **fda**, **R1D** is the sclerite on process **pva**, and **R1C** is the sclerite on process **pia** and in the area anterior of it (including the articulation **R1-R3** labeled **A3**); sclerite **R1B** = **R1C**+**D**; sclerite **R1F** = **R1A**+**C**.

**R3** – [*one-sided*]: ♂; right phallomere, a sclerite extending along the anterior ventral wall, also forming apodeme/groove **age**.

**Radius** – **R** [*paired*]: the stem giving rise to **RA** and **RP**; **RA** and **RP** are distinct from the base (i.e., there is no visible **R**) in forewings of *Metallyticus*, *Chaeteessa*, and *Mantoida* spp.

**Remigulum** – [*paired*]: area anteriorly delimited by the cubital fold, encompassing the preplicatum, plicatum, and plicatulum.

**Rhachis** – **rh** [*paired*]: ♀; a longitudinal external carina in the proximal half of the ventral wall of gonapophysis 9, with an Ω-like cross section; it fits into the aulax groove of gonapophysis 8 to form a sliding interlock, the olistheter; subapical part of rhachis sclerotized by **GPl9** (see Suppl. material 10: Extended abdominal glossary), apical part forming the membranous finger-shaped medial tine.

**Right phallomere** – [*paired, counterpart: left phallic complex*]: ♂; the right part of the phallomere complex, putatively derived from a right-dorsal primary phallic lobe; including the principal sclerites **R1** and **R3** as well as various processes.

***Rivetina*-type** – **ccsp7** [*paired*]: ♀; in *Rivetina* the coxosternite 7 (= subgenital plate) bears two large and strong ventral digging spines (Wieland 2013).

**Scape** – [*paired*]: first segment of the antennae, inserted on the cranium.

**Small slender sclerite** – **sss** [*paired*]: two small sclerites that are underneath the intercervicalia in a cervical fold; articulates with the preepisternite (see Wieland 2006: p. 53).

**Spermatheca** – **sp** [*unpaired*]: ♀; a median cuticular invagination of the posterior part of segment 8, opening upon an elevated area (spermathecal bulge) between the coxae 8, consisting of a slender spermathecal duct and a widened internal spermathecal bulb.

**Spermathecal bulb** – **spb** [*unpaired*]: ♀; the bulb-like most internal part of the spermatheca.

**Spermathecal bulge** – **sbu** [*unpaired*]: ♀; a median elevated area between the coxae 8 that bears the spermathecal opening, with the posterior and/or anterior parts overfolding the neighboring areas (folds **spbp** and **spba**, respectively).

**Spermathecal duct** – **spd** [*unpaired*]: ♀; the tube-like part of the spermatheca between the external opening and the internal spermathecal bulb.

**Spermathecal sclerotization** – **SP** [*unpaired*]: ♀; a median sclerotization located around the spermathecal opening; as currently known, it occurs either on the anterior (**spba**) or on the posterior (**spbp**) spermathecal bulge fold (see Suppl. material 10: Extended abdominal glossary); a sclerite on fold **spba** is called **SPa**, a sclerite on fold **spbp** is **SPp** (a continuous sclerite including both folds would be **SP**); the interpretation of both sclerites and whether they are independent sclerites or parts of the same large sclerite is unclear (the posterior **SPp** could be contributed by the true sternum 9 = **ST9**).

**Spiracle** – **si** [*paired*]: the segmental opening of the tracheal system (in abdominal segments 1–8, located near or behind mid-length upon the paratergal areas).

**Spur groove** – **tsg** [*paired*]: a depression on the posterior edge of the forefemur, which receives the tibial spur (Roy 1999).

**Stipes** – subdivision of the maxillary plate; articulates with the cardo; bears the galea, lacinia, and maxillary palpi (Snodgrass 1935).

**Stylus** – **sl9** [*paired*]: ♂; a basally articulated process (a true stylus) seated upon the hind edge of the ventral fold; representing distal parts of the limbs of segment 9 (plural: styli).

**Subantennal sulcus** – [*paired*]: sulcus that runs from the base of the antennal sclerite to the lower frons (plural: subantennal sulci).

**Subgena** – [*unpaired*]: a narrow, marginal area below the subgenal sulci; articulation point for gnathal appendages (Snodgrass 1935) (plural: subgenae).

**Subgenal sulcus** – [*paired*]: a lateral sulcus that corresponds anteriorly with the epistomal sulcus (plural: subgenal sulci).

**Supracoxal sulcus** – [*unpaired*]: a dorsal furrow that lies immediately above the forecoxae and divides the pronotum into a pro- and metazone; caused by an internal apodeme that supports the musculature of the forelegs (Wieland 2013).

**T-Shaped sclerite** – **tss** [*unpaired*]: a sclerite in the shape of a “T” that lies between the forecoxae in species with a relatively short pronotum; comprised of the basisternite and preepisternites (Wieland 2013: p. 51).

**Tarsus** – the fifth segment of the insect leg; articulates with the tibia; made up of five segments (tarsomeres) in Mantodea with Heteronutarsus, an exception, bearing four, (Wieland 2013: p. 161); the foot of the insect; bears euplantulae (plural: tarsi).

**Telson** – [*unpaired*]: the non-segmental posteroapical part of the body, bearing the anus; in Mantodea not represented by any structure but being a hypothetical membranous area surrounding the anus.

**Tergite** – **TG** [*unpaired*]: the undivided dorsal sclerite plate of an abdominal segment; in contrast to the coxosternite, not a composite sclerite.

**Tergite 10** – **TG10** [= ‘supraanal plate’] [*unpaired*]: the undivided dorsal sclerite plate of abdominal segment 10, whose lateral parts bend ventromesally to contact the paraprocts.

**Tibia** – **ti** [*paired*]: the fourth segment of the insect leg; in the foreleg, modified with spines for catching prey between the tibia and the femur; the foretibia terminates in a large, apical spur (plural: tibiae).

**Tibial spur** – **ts** [*paired*]: a frequently curved apical claw that terminates the tibia distally in all mantises except for *Chaeteessa* in which the spur was possibly secondarily reduced (Wieland 2013).

**Torus intercervicalis** – **tics** [*paired*]: a small, protruding shelf located on the posterior rim of the intercervical sclerite, which may carry setae (Wieland 2006; Wieland 2013).

**Transverse anterior part (of T-shaped sclerite)** – **tap** [*unpaired*]: a transverse sclerotization that forms the anterior part of the T-shaped sclerite; when the transverse anterior part is elongated due to a long prothorax, it forms the postcervical plate (Wieland 2013).

**Transverse carina (of the clypeus)** – [*unpaired*]: dorsal ridge of the clypeus; an outgrowth of the exoskeleton to which muscles attach.

**Transverse carina (of the lower frons)** – [*unpaired*]: dorsal protuberances of the lower frons; composed of frontal apodemes; an outgrowth of the exoskeleton to which muscles attach.

**Trochanter** – **tr** [*paired*]: the second and smallest segment of the praying mantis leg, articulating with the coxa at the coxo-trochanteral hinge; fused to the femur; lends insect leg flexibility (Snodgrass 1935).

**Tubercle** – cuticular bulges, bumps, knobs; can be present on the cranium, the pronotum, the forelegs, etc.

**Unguis** – [*paired*] claws, generally even in length with the only known exception found on the desert-dwelling *Heteronutarsus* (Wieland 2013); originate from the pretarsus (plural: ungues).

**Unguitractor Plate** – [*paired*]: attachment site for the pretarsal depressor muscle; depresses the ungues; located ventroproximally on the pretarsus (Gordh and Headrick 2001).

**Ventral cervical sclerite** – **vcs** [*unpaired, (occasionally) with (partial) median division*]: a narrow sclerite that medially traverses the cervical plate, can be divided.

**Ventral (segmental) fold 7** – **vf7** [*unpaired, fused from pair*]: ♀; the posteriorly directed and usually strongly posteriorly expanded transverse ventral fold of abdominal segment 7, which is ventrally sclerotized by coxosternite 7 and overlaps the ventral sides of segments 8 and 9 and most of the ovipositor ventrally; representing the projection of the limb bases of segment 7 (medially fused coxal lobes **cx7**).

**Ventral phallomere** – [*unpaired*]: ♂; the left-ventral part of the phallic complex, putatively derived from a mid-ventral or right-ventral primary phallic lobe; including the sclerites **L4A** and **L5**, the processes arising from the sclerites, and the opening of the ejaculatory duct.

**Ventrolateral coxal lobelet** – **cxvl** [*paired*]: ♀; a posteriorly and laterally directed projection from the posterolateral part of a coxa 8, located ventromesal to the dorsolateral coxal lobelet.

**Ventrolateral segmental expansion** – **vlse** [*paired*]: a flat expansion upon the lateral part of a coxosternite.

**Ventromedian expansion (of ventral fold)** – **vfme** [*unpaired to paired*]: a flat expansion resulting from a local lengthening of the ventral fold of a segment; the transition between unpaired and paired expansions is fluent, due to the degree of development of a median notch.

**Vertex** – [*unpaired*]: a point located equidistant to the compound eyes and above the ocelli; roughly describes the top of the head capsule.

**Vertical Process** – **vp** [*unpaired*]: a cuticular projection arising from the vertex of the cranium; lies posterior to, but not including, the epicranial sulcus.

**Vestibulum** – **vst** [*unpaired*]: ♀; the space enclosed above the ventral fold and beneath the ventral walls of segments 8 (posterior part) and 9.

**Vestibular Sclerite** – **VS7** [*unpaired, fused from pair*?]: ♀; a sclerite in the dorsal wall of the ventral fold.

**Visor** – **vs** [*paired*]: membranous area between the anterior wing margin and the anterior veinal margin (reference: ‘visor’ in Latin).

## 4. Conclusion

This work has sought to address a general lack of standardization for mantodean morphological terminology, specimen preparation, and linear morphometric measurements, especially as they pertain to taxonomy and systematics. By developing and implementing a standardized approach for Mantodea research, it is our hope that enthusiasts, students, and researchers alike will find collecting and interpreting taxonomic and morphological information on the charismatic praying mantises more accessible.

## Supplementary materials

Supplementary material 1 Head Capsule Terminology Data type: terminology Explanation note: Updated standardized terminology of external head capsule morphology with historical usage of topographically homologous terms. Morphological terminology of the external structures on the praying mantis head capsule are organized alphabetically by the updated standardardized terminology. Historical usage of topographically homologous structures are traced in reverse chronological order in subsequent columns. Plural spellings, abbreviations, and figure references for the standardized terminology are listed to aid in the identification of specific structures. † Levereault (1936: 219) referred to the region of the cranial vertex that contains the juxtaocular bulges as the “temporal sclerites,” but did not provide a name for the juxtaocular bulges themselves. This dataset is made available under the Open Database License (http://opendatacommons.org/licenses/odbl/1.0/). The Open Database License (ODbL) is a license agreement intended to allow users to freely share, modify, and use this Dataset while maintaining this same freedom for others, provided that the original source and author(s) are credited.Sydney K. Brannoch, Frank Wieland, Julio Rivera, Klaus-Dieter Klass, Olivier Béthoux, Gavin J. Svenson

Supplementary material 2 Forewing terminology Data type: terminology Explanation note: Updated standardized terminology of forewing venation and structure with historical usage of topographically homologous terms. Wing venation terminology is organized top-down from anterior to posterior-most structures. It is further organized by the two major wing venation patterns observed in species belonging to *MCM*-type (i.e., *Metallyticus*, *Chaeteessa*, *Mantoida*, and some fossil species; cells shaded blue) and *Mantis*-type (e.g., *Mantis religiosa*; cells shaded orange). Red dashed line indicate the delimitation between the Radius **R** and the Media **M**. Red frames around cells indicate particularities of an author’s morphological interpretation. When multiple species are considered by an author, the first occurrence of a term in a general context is indicated for the first species. This dataset is made available under the Open Database License (http://opendatacommons.org/licenses/odbl/1.0/). The Open Database License (ODbL) is a license agreement intended to allow users to freely share, modify, and use this Dataset while maintaining this same freedom for others, provided that the original source and author(s) are credited.Sydney K. Brannoch, Frank Wieland, Julio Rivera, Klaus-Dieter Klass, Olivier Béthoux, Gavin J. Svenson

Supplementary material 3 Hindwing terminology Data type: terminology Explanation note: Updated standardized terminology of hindwing venation and structure with historical usage of topographically homologous terms. Wing venation terminology is organized top-down from anterior to posterior-most structures. It is further organized by the two major wing venation patterns observed species belonging to *MCM*-type (i.e., *Metallyticus*, *Chaeteessa*, *Mantoida*, and some fossil species) and *Mantis*-type (e.g., *Mantis religiosa*). Red frames around cells indicated particularities of an author’s morphological interpretation. When multiple species are considered by an author, the first occurrence of a term in a general context is indicated for the first species. This dataset is made available under the Open Database License (http://opendatacommons.org/licenses/odbl/1.0/). The Open Database License (ODbL) is a license agreement intended to allow users to freely share, modify, and use this Dataset while maintaining this same freedom for others, provided that the original source and author(s) are credited.Sydney K. Brannoch, Frank Wieland, Julio Rivera, Klaus-Dieter Klass, Olivier Béthoux, Gavin J. Svenson

Supplementary material 4 Leg structure terminology Data type: terminology Explanation note: Updated standardized terminology of fore-, meso-, and metathoracic leg morphology with historical usage of topographically homologous terms. Morphological terminology of the external structures on the praying mantis fore-, meso-, and metathoracic leg are organized alphabetically by the updated standardardized terminology. Historical usage of topographically homologous structures are traced in reverse chronological order in subsequent columns. Plural spellings, abbreviations, and figure references for the standardized terminology are listed to aid in the identification of specific structures. This dataset is made available under the Open Database License (http://opendatacommons.org/licenses/odbl/1.0/). The Open Database License (ODbL) is a license agreement intended to allow users to freely share, modify, and use this Dataset while maintaining this same freedom for others, provided that the original source and author(s) are credited.Sydney K. Brannoch, Frank Wieland, Julio Rivera, Klaus-Dieter Klass, Olivier Béthoux, Gavin J. Svenson

Supplementary material 5 Thoracic structure terminology Data type: terminology Explanation note: Updated standardized terminology of external thoracic morphology with historical usage of topographically homologous terms. Morphological terminology of the external structures on the praying mantis thorax are organized alphabetically by the updated standardardized terminology. Historical usage of topographically homologous structures are traced in reverse chronological order in subsequent columns. Plural spellings, abbreviations, and figure references for the standardized terminology are listed to aid in the identification of specific structures. † The small slender sclerites (**sss**) of the cervix can only be seen in fresh or alcohol-preserved specimens. This dataset is made available under the Open Database License (http://opendatacommons.org/licenses/odbl/1.0/). The Open Database License (ODbL) is a license agreement intended to allow users to freely share, modify, and use this Dataset while maintaining this same freedom for others, provided that the original source and author(s) are credited.Sydney K. Brannoch, Frank Wieland, Julio Rivera, Klaus-Dieter Klass, Olivier Béthoux, Gavin J. Svenson

Supplementary material 6 Abdominal structure terminology Data type: terminology Explanation note: Updated standardized terminology of external abdominal structures with historical usage of topographically homologous terms. Morphological terminology of the external structures on the praying mantis abdomen are organized alphabetically by the updated standardardized terminology. Historical usage of topographically homologous structures are traced in reverse chronological order in subsequent columns. Plural spellings, abbreviations, and figure references for the standardized terminology are listed to aid in the identification of specific structures. This dataset is made available under the Open Database License (http://opendatacommons.org/licenses/odbl/1.0/). The Open Database License (ODbL) is a license agreement intended to allow users to freely share, modify, and use this Dataset while maintaining this same freedom for others, provided that the original source and author(s) are credited.Sydney K. Brannoch, Frank Wieland, Julio Rivera, Klaus-Dieter Klass, Olivier Béthoux, Gavin J. Svenson

Supplementary material 7 Male genital structure terminology Data type: terminology Explanation note: Updated standardized terminology of male genital morphology with historical usage of topographically homologous terms. Morphological terminology of the male praying mantis genital complex are organized alphabetically by the updated standardardized terminology. Historical usage of topographically homologous structures are traced in reverse chronological order in subsequent columns. Plural spellings, abbreviations, and figure references for the standardized terminology are listed to aid in the identification of specific structures. This dataset is made available under the Open Database License (http://opendatacommons.org/licenses/odbl/1.0/). The Open Database License (ODbL) is a license agreement intended to allow users to freely share, modify, and use this Dataset while maintaining this same freedom for others, provided that the original source and author(s) are credited.Sydney K. Brannoch, Frank Wieland, Julio Rivera, Klaus-Dieter Klass, Olivier Béthoux, Gavin J. Svenson

Supplementary material 8 Female genital structure terminology Data type: terminology Explanation note: Updated standardized terminology of female genital morphology with historical usage of topographically homologous terms. Morphological terminology of the female praying mantis genital complex are organized alphabetically by the updated standardardized terminology. Historical usage of topographically homologous structures are traced in reverse chronological order in subsequent columns. Plural spellings, abbreviations, and figure references for the standardized terminology are listed to aid in the identification of specific structures. This dataset is made available under the Open Database License (http://opendatacommons.org/licenses/odbl/1.0/). The Open Database License (ODbL) is a license agreement intended to allow users to freely share, modify, and use this Dataset while maintaining this same freedom for others, provided that the original source and author(s) are credited.Sydney K. Brannoch, Frank Wieland, Julio Rivera, Klaus-Dieter Klass, Olivier Béthoux, Gavin J. Svenson

Supplementary material 9 Abdominal terminology discussion Data type: discussion Explanation note: Continued discussion on the abdominal terminology used within the publication. This dataset is made available under the Open Database License (http://opendatacommons.org/licenses/odbl/1.0/). The Open Database License (ODbL) is a license agreement intended to allow users to freely share, modify, and use this Dataset while maintaining this same freedom for others, provided that the original source and author(s) are credited.Sydney K. Brannoch, Frank Wieland, Julio Rivera, Klaus-Dieter Klass, Olivier Béthoux, Gavin J. Svenson

Supplementary material 10 Extended abdominal glossary Data type: glossary Explanation note: Extended glossary of Mantodea abdominal terminology. This dataset is made available under the Open Database License (http://opendatacommons.org/licenses/odbl/1.0/). The Open Database License (ODbL) is a license agreement intended to allow users to freely share, modify, and use this Dataset while maintaining this same freedom for others, provided that the original source and author(s) are credited.Sydney K. Brannoch, Frank Wieland, Julio Rivera, Klaus-Dieter Klass, Olivier Béthoux, Gavin J. Svenson
